# Metal pre-intercalation promotes water-mediated proton-coupled electron transfer in layered δ-MnO_2_ for aqueous pseudocapacitive energy storage

**DOI:** 10.1038/s41467-026-76022-4

**Published:** 2026-07-28

**Authors:** Huajie Ze, Yongkwon Song, Xijun Wang, Weiyan Ni, Xiaobing Hu, Jianan Erick Huang, Zeyan Liu, Hengzhou Liu, Xiao-Yan Li, Randall Q. Snurr, Mark C. Hersam, Ke Xie, Edward H. Sargent

**Affiliations:** 1 Department of Chemistry, Northwestern University, Evanston, IL USA; 2 Department of Electrical and Computer Engineering, Northwestern University, Evanston, IL USA; 3 Department of Materials Science and Engineering, Northwestern University, Evanston, IL USA; 4 Department of Chemical and Biological Engineering, Northwestern University, Evanston, IL USA

**Keywords:** Electrochemistry, Batteries, Batteries

## Abstract

The charge storage capacitance of δ-MnO_2_-based pseudocapacitors stems from a combination of bulk cation intercalation/deintercalation and surface proton chemisorption/desorption. Here, we investigate the mechanistic origins of the enhanced capacitance in δ-MnO_2_ with pre-intercalated Cu^2+^. To this end, we synthesize Au-core/δ-MnO_2_-shell nanostructures with and without Cu^2+^ pre-intercalation, enabling real-time in situ spectroscopic monitoring of structure-function relationships during electrochemical cycling. Transition metal pre-intercalation preserves interlayer-confined water, which in turn supports proton-coupled charge storage via the reversible reaction of MnO_2_ + H_2_O + e^-^ ⇌ MnOOH + OH^-^. This confined water forms a hydrogen-bonded network that lowers the energy barrier for proton transport within the interlayer space. Similar mechanistic transition is also evident in δ-MnO_2_ systems pre-intercalated with other transition metal ions, such as Co^2+^ and Mg^2+^. By tuning the MnO_2_ shell thickness, we decouple the relative contributions of proton- and cation-driven processes, revealing that proton intercalation delivers a markedly higher specific capacitance than cation intercalation. Electrolyte-dependent studies further reveal that Cu^2+^ pre-intercalation promotes OH^-^ transport within the interlayer space while preserving proton accessibility at active sites. These findings suggest that proton-coupled transport may offer further increases in charge storage performance in pseudocapacitors.

## Abstract (web summary)

Electrochemical charge storage in MnO_2_ is limited by slow ion transport and structural instability. Here, the authors show that metal pre-intercalation enables water-mediated proton-coupled charge storage, enhancing capacitance and reversibility in layered MnO_2_.

## Introduction

Pseudocapacitors store charge via fast and reversible Faradaic reactions that occur at or near an electrode surface, enabling rapid ion transport and high-power applications^1,2^. Layered structures serve as particularly good hosts for redox processes, providing rapid diffusion pathways for ionic species^3^. A high-performing such material is the birnessite phase of MnO_2_ (δ-MnO_2_), in which alkali ions are accommodated within the interlayer galleries formed by stacked sheets of edge-sharing MnO_6_ octahedra^4^. Capacitance in δ-MnO_2_ originates from two principal mechanisms^5,6^: (i) the intercalation/deintercalation of cations (e.g., Li^+^, Na^+^, K^+^) or protons from the electrolyte into the bulk lattice: MnO_2_ + M^+^ + e^-^ ⇌ MnOOM, typically following partial or complete desolvation; (ii) the surface chemisorption and desorption of cations (e.g., Li^+^, Na^+^, K^+^) or protons: (MnO_2_)_surface_ + M^+^ + e^-^ ⇌ (MnOOM)_surface_. Both charge-storage processes primarily occur on or near the surface^7,8^, with recent studies suggesting a dual-mode mechanism in which cation intercalation dominates in the bulk while proton-coupled surface redox prevails at the interface^9^.

However, metal cation intercalation is hindered by sluggish diffusion kinetics, which causes significant lattice strain. During the discharge process, progressive reduction of Mn^4+^ to Mn^3+^ induces Jahn-Teller (J-T) distortion, undermining the MnO_6_ framework and leading to structural degradation and capacitance loss^10,11^. One way to tackle this is the pre-intercalation of guest metal ions, such as Mg^2+^, Cu^2+^, and Co^2+^, in place of customary alkali cations^12–15^. These multivalent ions induce compressive strain within the MnO_6_ lattice, stabilizing the layered structure. The challenges associated with reliance on cation intercalation have motivated interest in proton intercalation: the low mass, high charge-to-mass ratio and rapid kinetics of protons facilitate efficient charge transport, and their small size minimizes lattice strain during insertion and extraction, increasing reversibility and cycle life^16–19^. In neutral to alkaline aqueous media, water molecules form the electric double layer and also serve as the proton donor. When confined within the interlayer galleries of MnO_2_, water molecules adopt structural motifs distinct from those of bulk or interfacial-adsorbed water, as nanoconfinement perturbs its native hydrogen-bonding network^20^.

**Fig. 1:**
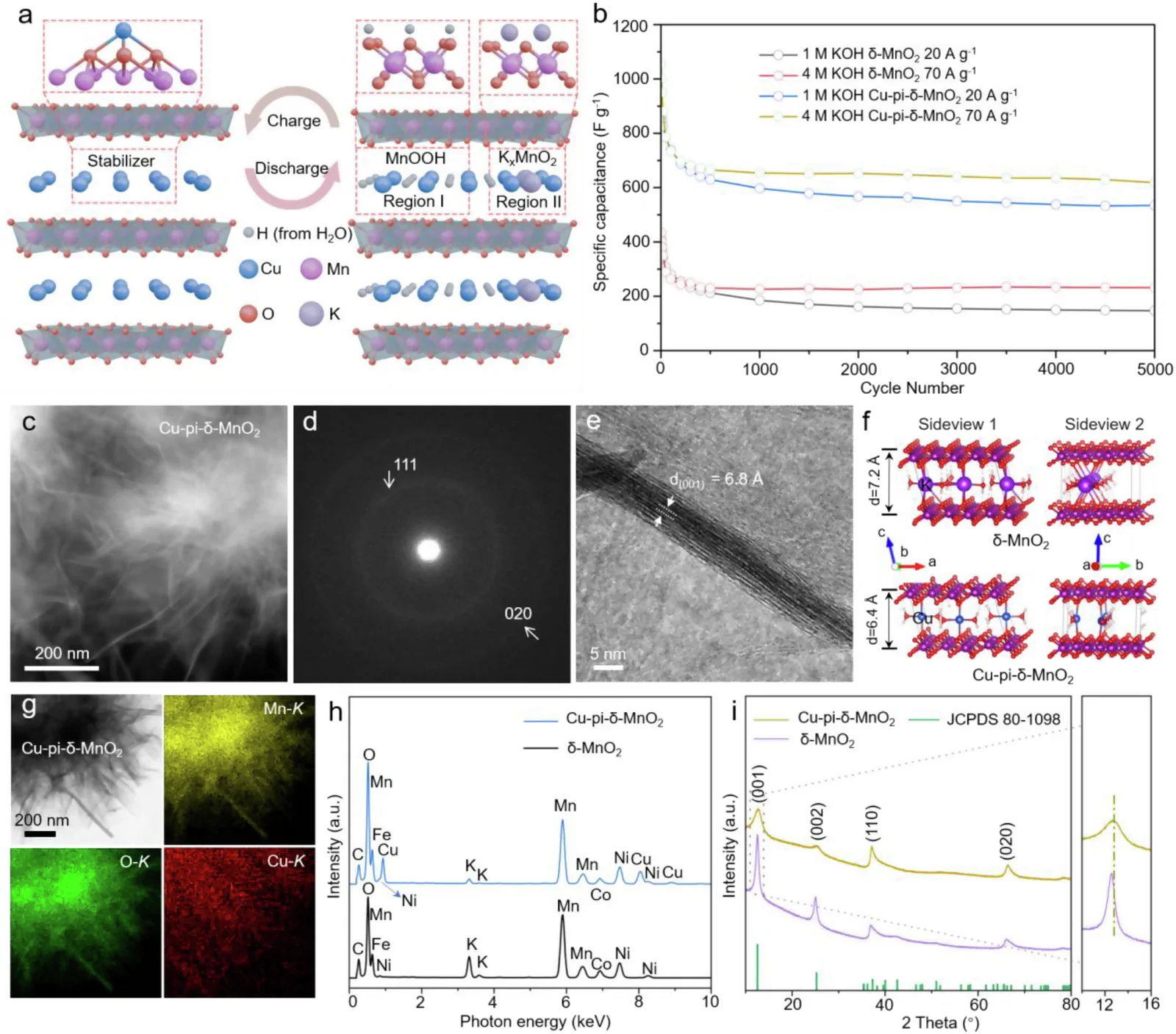
Structure and capacitance performance of δ-MnO_2_ and Cu-pi-δ-MnO_2_. **a** Schematic depicting the enhanced capacitance mechanism in δ-MnO_2_ through Cu^2+^ pre-intercalation, enabling K^+^ and H_2_O co-intercalation for bulk charge storage. **b** Capacitance comparison of δ-MnO_2_ and Cu-pi-δ-MnO_2_ at varied electrolyte concentrations and specific currents within a 1.2 V potential window. **c–e** HAADF, SAED pattern, and HRTEM image of synthesized Cu-pi-δ-MnO_2_. **f** DFT calculations of the interlayer spacing in K_0.125_MnO_2_·0.25H_2_O and Cu_0.125_MnO_2_·0.25 H_2_O systems. Structural models were optimized by explicitly incorporating intercalated water molecules to ensure accurate coordination environments for K^+^ and Cu^2+^ ions within the layered MnO_2_ framework. **g** ABF image of Cu-pi-δ-MnO_2_, and corresponding EDS mapping of Mn, O, and Cu. **h** Quantitative elemental composition (Mn, O, K, and Cu) of δ-MnO_2_ and Cu-pi-δ-MnO_2_ derived from EDS mapping. The Fe, Co, and Ni atoms primarily originate from the microscope pole pieces and are considered artifacts. The presence of Ni is mainly due to the use of a Ni grid. **i** XRD analysis of δ-MnO_2_ and Cu-pi-δ-MnO_2_.

Variations in the local environment of water molecules often result in distinct shifts in O-H vibrational modes, providing unique spectroscopic signatures of nanoconfined and interfacial water and enabling the identification of proton transfer pathways^21–23^. As a result, we herein employ in situ surface-enhanced Raman spectroscopy (SERS), in combination with electrochemical measurements, to elucidate the distinct charge storage behaviors of protons and cations in Cu^2+^-pre-intercalated δ-MnO_2_ (Fig. 1a). Our study aims to differentiate the electrochemical response of δ-MnO_2_ before and after Cu^2+^ pre-intercalation, and links the observed enhancement in capacitance to the emergence of a proton-coupled redox mechanism mediated by interlayer-confined water. By varying the electrolyte composition, we reveal how ionic competition at active sites governs this proton-driven charge storage. Notably, similar mechanistic transitions are observed with Co^2+^ an Mg^2+^ pre-intercalation, suggesting a generalizable proton-coupled pathway across transition metal-intercalated MnO_2_ systems. Overall, the results point to pathways to next-generation pseudocapacitors governed by a highly reversible, proton-coupled mechanism, potentially without the need for synthetically demanding ultrathin architectures.

## Results

### Structure-performance in δ-MnO_2_ and transition-metal-doped δ-MnO_2_

δ-MnO_2_ (birnessite) is synthesized via hydrothermal methods, using KMnO_4_ as the oxidant and MnSO_4_ as the reducing agent. Its layered structure, with an interlayer spacing of ~7 Å, accommodates K^+^ ions as structural pillars within the interlayer galleries. However, this architecture degrades upon electrochemical cycling due to the limited stabilizing effect of K^+^. For these reasons, pre-intercalation of Cu^2+^ ions (Cu-pi-δ-MnO_2_) is employed, replacing K^+^ with Cu^2+^ ions to strengthen electrostatic interactions with interlayer oxygen atoms and reinforce the lattice framework. Cu^2+^ pre-intercalation markedly enhances the capacitance, yielding ~560 F g^−1^ at 20 A g^−1^ in 1 M KOH, compared to ~160 F g^−1^ for pristine δ-MnO_2_ (Fig. 1b). Increasing the electrolyte concentration to 4 M KOH further improves the capacitance of both systems, with Cu-pi-δ-MnO_2_ reaching ~650 F g^−1^ even at a high specific current of 70 A g^−1^^24–26^.

From high-angle annular dark field (HAADF), selected area electron diffraction (SAED), and high-resolution transmission electron microscopy (HRTEM), we observe a decrease in the interlayer spacing from 0.73 to 0.68 nm upon Cu^2+^ pre-insertion (Fig. 1c–e and Supplementary Figs.1–2; a trend also predicted in density functional theory (DFT), with calculated interlayer spacing decreasing from 0.72 to 0.64 nm upon complete cation exchange, Fig. 1f). Annular bright field (ABF) imaging and corresponding energy-dispersive spectroscopy (EDS) mapping (Fig. 1g, h and Supplementary Figs. 3–4) show a uniform elemental distribution and partial substitution of K^+^ by Cu^2+^. X-ray diffraction (XRD) patterns (Fig. 1i) indicate the retention of the birnessite δ-MnO_2_ phase (JCPDS: 80-1098) following Cu^2+^ pre-intercalation. A noticeable shift of the (001) reflection toward higher angles is observed, indicating interlayer contraction, which we attribute to the smaller ionic radius of Cu^2+^ (0.73 Å) relative to K^+^ (1.38 Å). Additionally, the (001) peak of Cu-pi-δ-MnO_2_ exhibits noticeable broadening, likely due to local lattice distortions associated with modified Cu-O-Mn coordination environments. Inductively coupled plasma optical emission spectroscopy (ICP-OES) results (Supplementary Table 1) show an atomic ratio corresponding to K_0.125_MnO_2_ for δ-MnO_2_ and K_0.011_Cu_0.089_MnO_2_ for Cu-pi-δ-MnO_2_. The shorter Cu-O bond length (2.19 Å) compared to Mn-O (2.24 Å) in the Cu-pi-δ-MnO_2_ structure is believed to contribute to reduced lattice strain during electrochemical cycling. X-ray photoelectron spectroscopy (XPS) analysis (Supplementary Figs. 5–6) indicates that Mn^4+^ remains dominant, with partial Mn reduction upon Cu^2+^ pre-intercalation. Concurrently, the O 1 *s* spectrum shows an increased population of Mn-OH/H_2_O species. These changes together compensate for the additional charge introduced by Cu^2+^ pre-intercalation. While Cu^2+^ is clearly present, this structural information does not on its own answer why, or how, the capacitance increased.

### Cu^2+^ Pre-intercalation promotes a proton-intercalation pathway

**Fig. 2:**
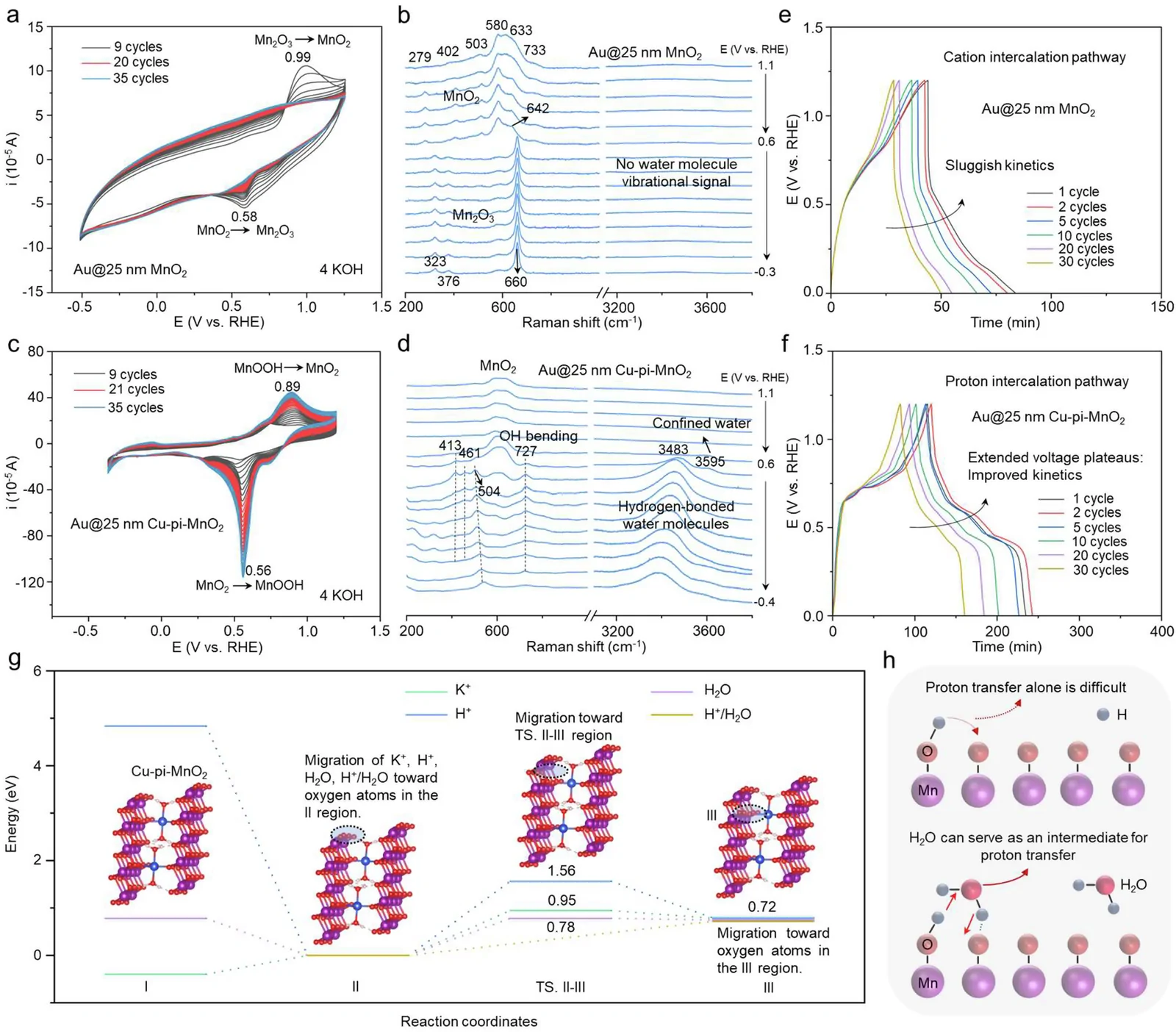
Structural analysis and capacitance performance of δ-MnO_2_ and Cu-pi-δ-MnO_2_. **a** CV curves of Au@25 nm MnO_2_. **b** In situ electrochemical SERS study of the discharge process on Au@25 nm MnO_2_ surfaces. **c** CV tests of Au@25 nm Cu-pi-δ-MnO_2_. **d** In situ electrochemical SERS study of the discharge process on Au@25 nm Cu-pi-δ-MnO_2_ surfaces. GCD profiles of Au@25 nm MnO_2_ (**e**) and Au@25 nm Cu-pi-δ-MnO_2_ (**f**) measured within a potential window of 1.2 V at 20 A g^-1^. **g** DFT-simulated energy profiles for the interlayer migration of H^+^, K^+^, H_2_O, and H^+^/H_2_O in Cu-pi-δ-MnO_2_. **h** Schematic illustration of proton transport within the MnO_2_ lattice. Direct proton migration is energetically unfavorable, whereas water-mediated pathways enable proton hopping through hydrogen-bond networks. All electrochemical measurements in a-f were conducted in 4 M KOH. All CV measurements in this work were performed at a scan rate of 50 mV s^−1^. All potentials are referenced to the reversible hydrogen electrode (RHE). Throughout all figures, E and i represent the applied potential and current, respectively.

We constructed Au@δ-MnO_2_ and Au@Cu-pi-δ-MnO_2_ nanostructures with 25 nm shells (Supplementary Fig. 7), in which the plasmonic Au core provides strong Raman enhancement, enabling us to monitor charge-storage pathways within the inner MnO_2_ layers. In the case of δ-MnO_2_, we confirm six phonon modes at ~733, ~633, ~580, ~503, ~402, and ~279 cm^−1^ (Supplementary Fig. 8). Upon Cu^2+^ pre-intercalation, these exhibit notable broadening, consistent with Cu-O-Mn bond formation and lattice strain, in agreement with the XRD analysis. Cyclic voltammetry (CV) shows that the pure MnO_2_ shell undergoes an irreversible redox process, characterized by a reduction peak at ~0.58 V vs. RHE and an oxidation peak at ~0.99 V vs. RHE, accompanied by rapid dissolution in 4 M KOH (Fig. 2a). Raman spectra also show Mn(III)-O vibrations, characteristic of Mn_2_O_3_ (Fig. 2b and Supplementary Fig. 9), indicating a phase transformation which we account for via K^+^ intercalation. The electrochemical process reduces Mn^4+^ to J-T-active Mn^3+^, known to produce lattice distortion. It is worth noting that the absence of water-related Raman bands indicates that cation intercalation occurs in a dehydrated state.

By contrast, Cu-pi-δ-MnO_2_ exhibits a more reversible redox process, evidenced by the progressive intensification of redox peaks at ~0.55 V vs. RHE during CV cycling (Fig. 2c). At ~0.6 V vs. RHE, a high-frequency O-H stretching band appears at ~3595 cm^-1^ (Fig. 2d), characteristic of interlayer-confined water. Given the rapid decay of Au-core-induced Raman enhancement with increasing MnO_2_ shell thickness^27^, this signal is attributed to confined water within the MnO_2_ interlayers, with the elevated frequency reflecting disruption of the hydrogen-bond network under nanoconfinement. As the potential sweeps negatively to 0.4 V vs. RHE, a new band appears at ~3483 cm^–1^, assigned to hydrogen-bonded water. This shift indicates that deeper polarization drives additional water molecules into the interlayers, facilitating hydrogen-bonding interactions that were suppressed by nanoconfinement in the case of pure MnO_2_. Additionally, in the low-frequency region, vibrational features corresponding to O-H bending ( ~ 727 cm^–1^) and Mn-O lattice modes ( ~ 413, ~461, and ~504 cm^–1^) emerge, consistent with the formation of MnOOH. These spectral assignments are also supported by D_2_O isotope labeling, which shows red-shifted bands for confined water ( ~ 2663 cm^–1^), hydrogen-bonded water ( ~ 2563 cm^–1^), and O-D bending ( ~ 590 cm^−1^) (Supplementary Fig. 10).

These observations of Cu-pi-δ-MnO_2_ indicate a charge storage mechanism governed by the interlayer reaction: MnO_2_ + H_2_O + e^-^ ⇌ MnOOH + OH^-^, mediated by confined water molecules within the layered structure. Galvanostatic charge-discharge (GCD) analysis (Fig. 2e, f) reveals that the Cu-pi-δ-MnO_2_ system exhibits a more pronounced voltage plateau, indicative of a more reversible redox process and enhanced reaction kinetics. This is related to the more dominant contribution from proton-coupled intercalation compared to the cation-intercalation-dominated δ-MnO_2_. From DFT calculations (Supplementary Figs. 11–14 and Fig. 2g), the energy barrier of interlayer migration is significantly higher for bare protons (1.56 eV, H^+^) than for cations (0.95 eV, K^+^), whereas the presence of interlayer water molecules lowers this barrier by mediating proton transport (0.78 eV for pure water, 0.72 eV for H^+^/H_2_O). Optimized structural models and DFT-calculated energy data are provided in Supplementary Data 1–4. Interlayer-confined water thus functions not only as a proton reservoir but also as a dynamic facilitator of proton transfer (Fig. 2h). A clear understanding of proton- and cation-based charge-storage mechanisms is critically important for other electrochemical energy-storage systems, such as Zn/MnO_2_ batteries^28,29^. These findings prompted us to investigate whether similar changes occur with other pre-intercalated metal ions, such as Co^2+^ and Mg^2+^^13–15^. XRD measurements of Co-pi-δ-MnO_2_ and Mg-pi-δ-MnO_2_ similarly reveal a contraction in interlayer spacing, confirming successful pre-intercalation (Supplementary Fig. 15). Our spectroscopic and electrochemical results reveal that pre-intercalation of Co^2+^ and Mg^2+^, which replace native K^+^ ions, facilitates the insertion of interlayer-confined water and similarly promotes proton-coupled intercalation (Supplementary Figs. 16−17). Collectively, these results establish the generality of the proton-coupled charge-storage mechanism enabled by metal pre-intercalation in layered MnO_2_.

### Proton insertion outperforms cation insertion in specific capacitance

**Fig. 3:**
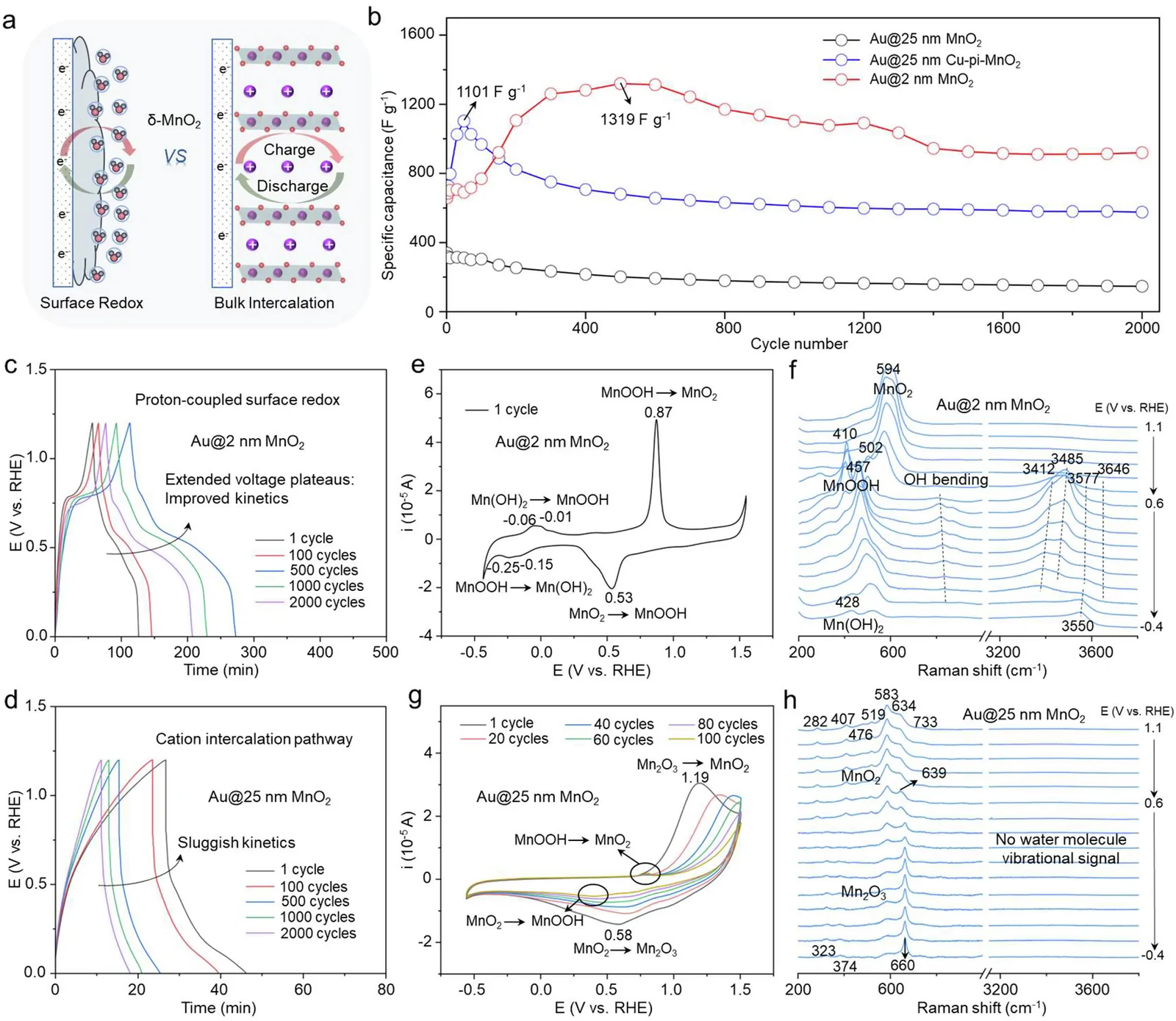
Electrochemical and spectroscopic studies of proton-intercalation-based charge storage vs. cation-intercalation mechanisms. **a** Schematic illustration of the charge storage mechanisms in the inner and outer layers of MnO_2_; **b** Capacitance comparison between Au@2 nm MnO_2_, Au@25 nm MnO_2_ and Au@25 nm Cu-pi-δ-MnO_2_ NPs over 2000 cycles within a potential window of 1.2 V at 20 A g^−1^. The capacitance values were obtained at selected cycles (1, 5, 10, 30, 50, 70, 100, 150, 200, and every 100 cycles thereafter). GCD profiles of Au@2 nm MnO_2_ (**c**) and Au@25 nm δ-MnO_2_ NPs (**d**) measured within a potential window of 1.2 V at 20 A g^–1^. CV cycling and in situ SERS analysis of the discharge process for Au@2 nm MnO_2_ (**e, f**) and Au@25 nm MnO_2_ (**g, h**) in 0.1 M KOH.

Based on our observations and prior reports that pristine birnessite-type MnO_2_ can exhibit enhanced capacitance via proton-associated surface or near-surface faradaic reactions^7,30^, we attribute the capacitance enhancement of Cu-pi-δ-MnO_2_ to promoting proton-coupled charge storage. To test this hypothesis, we engineered Au@MnO_2_ core-shell structures with varied MnO_2_ shell thicknesses to decouple surface-dominated proton-coupled processes from bulk cation intercalation (Fig. 3a)^1,9^. This design serves as a mechanistic proof-of-concept, since the scalable synthesis of ultrathin MnO_2_ remains technically challenging. Given the susceptibility of such thin layers to dissolution in strongly alkaline media (a point we revisit later), we reduced the electrolyte concentration to 0.1 M KOH to prevent degradation. Under these conditions, the Au@2 nmMnO_2_ system exhibits a markedly enhanced specific capacitance, reaching ~1319 F g^−1^ at 20 A g^−1^, exceeding that of its thicker analogue Au@25 nm MnO_2_ (Fig. 3b). To account for the different Au contents at identical MnO_2_ loadings, the corresponding capacitance responses of the Au nanoparticles in both systems are provided in Supplementary Table 2. Consistent with these electrochemical trends, Au@2 nm MnO_2_ exhibits GCD profiles with a more pronounced voltage plateau and highly reversible redox behavior, indicative of improved reaction kinetics (Fig. 3c, d). CV and in situ Raman spectroscopy reveal a proton-coupled electron transfer (PCET) process in the ultrathin 2 nm MnO_2_ system (Fig. 3e, f), as evidenced by the emergence of characteristic Mn-O vibrations ( ~ 502, ~457, and ~410 cm^−1^), O-H bending ( ~ 807 cm^−1^), and O-H stretching ( ~ 3577 cm^−1^) of MnOOH, together with bands associated with interfacial water ( ~ 3412, ~3485, and ~3646 cm^–1^). The vibrational assignments are further corroborated by isotopic (D_2_O) Raman experiments (Supplementary Fig. 18). Notably, the MnOOH species formed during this process is a transient and reversible intermediate that converts back to MnO_2_ upon removal of the applied potential (Supplementary Fig. 19). As an additional control, we also present the CV and in situ SERS results of bare Au nanoparticles, which further confirm that the electrochemical and spectroscopic signals observed in the core-shell electrodes originate predominantly from the MnO_2_ shell rather than the Au core (Supplementary Fig. 20). Au@25 nm MnO_2_ undergoes a cation-driven intercalation process followed by irreversible conversion to Mn_2_O_3_ even in 0.1 M KOH (Fig. 3g, h), similar to the degradation observed in 4 M KOH. Cu^2+^ pre- intercalation within the 25 nm MnO_2_ shell leads to a substantial enhancement in capacitance reaching a maximum value of approximately 1101 F g^–1^ at 20 A g^–1^, demonstrating that Cu incorporation promotes charge storage (Fig. 3b). Consistent with this enhancement, GCD profiles of Au@25 nm Cu-pi-δ-MnO_2_ measured in 0.1 M KOH, which closely resemble those of the 2 nm system and exhibit a more pronounced voltage plateau indicative of a proton-dominated redox process (Supplementary Fig. 21a). In addition, spectroscopic results show that clear signatures associated with confined interlayer water persist even after 2000 charge-discharge cycles (Supplementary Fig. 21b). Further evidence is provided by CV and in situ Raman spectroscopy reveal clear signatures of a PCET process in Au@25 nm Cu-pi-δ-MnO_2_ (Supplementary Fig. 22). Notably, at the lower electrolyte concentration (0.1 M KOH), this PCET process proceeds with slower intercalation kinetics, as evidenced by the delayed appearance of MnOOH-related CV features and the delayed emergence of interfacial water and MnOOH-associated Raman signatures, compared to those observed in 4 M KOH. This contrasts with the behavior at higher concentrations and explains the higher capacitance observed in 4 M KOH compared to 1 M KOH (Fig. 1b). We next investigated the influence of electrolyte cations and anions on the proton intercalation process, with a focus on competitive interfacial ion interactions.

### Roles of OH^-^ and cations in proton-coupled redox processes

**Fig. 4:**
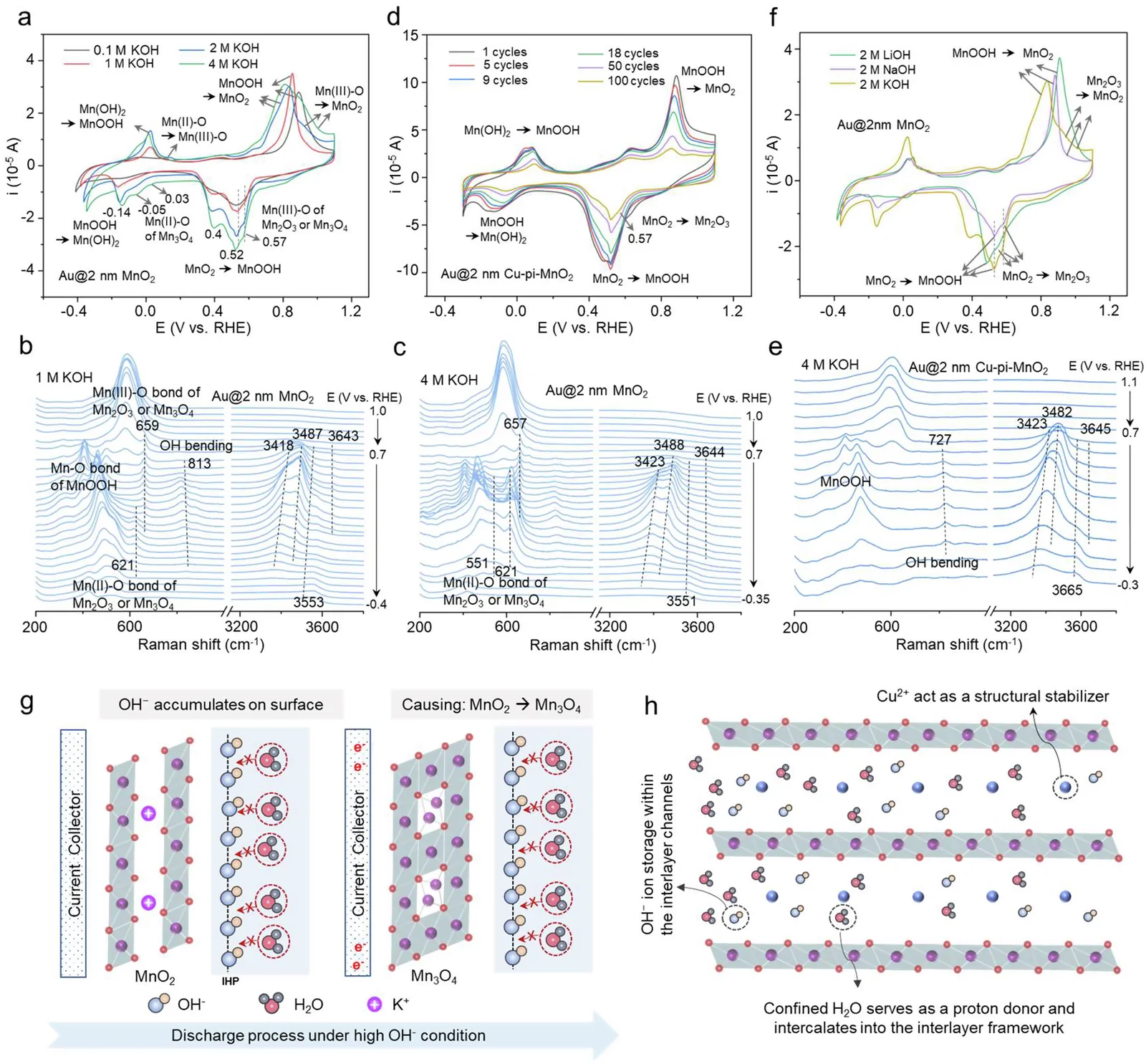
In situ studies of the effects of OH^-^ and cation concentration on proton-involved surface redox processes occurring on the outer layer of MnO_2_. **a** CV analysis of Au@2 nm MnO_2_ in alkaline electrolytes of varying concentrations (0.1-4 M KOH); In situ electrochemical SERS monitoring of the discharge process on Au@2 nm MnO_2_ surfaces in **b** 1 M and **c** 4 M KOH; **d** CV results of Au@2 nm Cu-pi-δ-MnO_2_ with increasing scanning cycles in 4 M KOH; **e** In situ electrochemical SERS monitoring of the discharge process on Au@2 nm Cu-pi-δ-MnO_2_ surfaces in 4 M KOH; **f** CV analysis of Au@2 nm MnO_2_ in 2 M alkaline electrolytes (LiOH, NaOH, KOH). The schematic illustration of the discharge mechanism for the surface redox process of δ-MnO_2_ (**g**) and Cu-pi-δ-MnO_2_ (**h**) in a highly alkaline environment.

To examine how interfacial ion chemistry regulates proton intercalation, we varied the electrolyte concentration while maintaining a fixed ultrathin MnO_2_ shell thickness ( ~ 2 nm), under which proton participation dominates and strong Raman enhancement is achieved. Comparative studies of Au@2 nm MnO_2_ and Au@2 nm Cu-pi-δ-MnO_2_ were conducted across a suite of conditions of alkaline pH. CV and in situ SERS (Fig. 4a, b) reveal new redox features emerging at ~0.57and ~ 0.03 V vs. RHE in 1 M KOH, with corresponding Raman bands at ~659 and ~622 cm^–1^. These signals intensify with increasing concentration (Supplementary Fig. 23), accompanied by a new peak at –0.05 V vs. RHE and a ~ 560 cm^–1^ band in 4 M KOH (Fig. 4c). These bands are attributed to Mn(III)-O ( ~ 659 cm^–1^) and Mn(II)-O ( ~ 622, ~560 cm^–1^) vibrations, consistent with the formation of Mn_2_O_3_ or Mn_3_O_4_ (Supplementary Fig. 9). The absence of isotope shifts in D_2_O (Supplementary Fig. 24) also confirms that these species are not involved in proton-coupled process. The MnO_2_ shell fully dissolves after 100 cycles in 4 M KOH, while remaining intact in 0.1 M KOH after 500 cycles (Supplementary Figs. 25–26), linking these features to structural degradation. In contrast, Cu-pi-δ-MnO_2_ shows no clear Mn(III)/Mn(II) signatures, even in 4 M KOH (Fig. 4d, e and Supplementary Fig. 27), with only weak Mn(III)-O signals after extended cycling (Supplementary Fig. 28), suggesting Cu^2+^ pre-intercalation suppresses irreversible phase transitions.

One prior study found that, at 0.1 M OH^-^, neither varying cation identity nor increasing cation concentration induced these new redox features, indicating that MnO_2_ degradation arises from OH^-^ accumulation rather than from cation effects^7^. Under more alkaline conditions (2 M OH^-^), however, cation identity markedly affects the proton-coupled redox process (Fig. 4f). CV profiles show suppressed proton activity in LiOH and enhanced activity in NaOH, correlating with hydration radii (Li^+^ ( ~ 3.82 Å) > Na^+^ ( ~ 2.76 Å) and associated electric double layer (EDL) thickness. While K^+^ ( ~ 2.32 Å) forms the thinnest EDL and generates the strongest interfacial field, its redox activity is unexpectedly lower than Na⁺. We attribute this to field-enhanced OH^-^ adsorption displacing interfacial water and hindering proton transfer. Consequently, proton-coupled redox processes proceed more slowly in 2 M KOH than in 2 M NaOH, as evidenced by similar MnOOH + OH^-^ + e^-^ ⇌ MnO_2_ + H_2_O features in 2 M NaOH (Fig. 4f) and 1M KOH (Fig. 4a).

To elucidate the role of OH^-^ more directly, we conducted CV and in situ Raman measurements under ultra-high alkalinity (15 M KOH), where δ-MnO_2_ exhibits a sharp reduction peak, transient formation of Mn(III)- O species, and rapid dissolution (Supplementary Figs. 29, 30). No MnOOH-related Raman bands are detected, and the weak water signal closely resembles that observed on bare Au nanoparticles, indicating it originates from the Au substrate (Supplementary Fig. 31). These results suggest that OH^-^ accumulates at the interface, displacing active sites otherwise occupied by interfacial H_2_O and thereby suppressing proton- coupled processes. In addition, hydrogen bonding between OH^-^ and interfacial H_2_O may also impede water mobility and interaction with the MnO_2_ surface. Restricted water accessibility under these conditions facilitates cation intercalation into the MnO_2_ lattice, initiating phase transitions to Mn_2_O_3_ or Mn_3_O_4_ (Fig. 4g). The pronounced dissolution observed under such high alkalinity is attributed to the formation of OH-coordinated Mn^2+^ complexes and structural degradation driven by J-T distortion. Cu^2+^ pre-intercalation stabilizes the system by providing defined channels for OH^-^ sequestration within the interlayer space, thereby limiting surface OH^-^ accumulation (Fig. 4h). Collectively, these findings underscore the critical role of ion pairs in the electrolyte in governing the charge storage process.

## Discussion

In this work, we establish an electrochemical-spectroscopic framework that enables direct, molecular-level interrogation of charge-storage pathway evolution in layered MnO_2_. By integrating in situ SERS with electrochemical measurements, we provide direct spectroscopic evidence that Cu^2+^ pre-intercalation promotes proton-coupled redox processes, which constitute one of the key contributors to the enhanced capacitance of Cu-pi-δ-MnO_2_, given the intrinsically fast kinetics and high charge-storage efficiency of proton-coupled reactions. Moreover, we directly identify, through spectroscopic signatures, nanoconfined interlayer water as an active and persistent participant in charge storage, functioning as an intrinsic proton reservoir. The associated MnO_2_-to-MnOOH transformation confirms a water-mediated PCET pathway enabled by Cu^2+^ pre-intercalation. Extending this electrochemical and spectroscopic framework to other metal-pre-intercalated MnO_2_ systems suggests that this proton-coupled mechanism likely represents a generalizable pathway across transition-metal-intercalated layered oxides. Beyond identifying the dominant charge carrier, this work reveals interfacial ion competition as a critical regulator of proton-coupled charge storage and highlights proton intercalation as a central design lever for achieving enhanced capacitance and reversibility in aqueous pseudocapacitors.

## Methods

### Chemicals and materials

All chemicals were used as received without further purification. Potassium permanganate (KMnO_4_, ACS reagent, ≥99.0%), manganese(II) sulfate monohydrate (MnSO_4_·H_2_O, ACS reagent, ≥98%), copper(II) nitrate hemi(pentahydrate) (Cu(NO_3_)_2_·2.5H_2_O, ACS reagent, 98%), potassium hydroxide (KOH, ACS reagent, ≥85%, pellets), gold(III) chloride trihydrate (HAuCl_4_·3H_2_O, ≥99.9% trace metals basis), and trisodium citrate dihydrate (Na_3_C_6_H_5_O_7_·2H_2_O, ACS reagent grade, ≥99.0%) were obtained from Sigma-Aldrich. Hg/HgO reference electrodes were purchased from CHI Instruments. Ultrahigh-purity Ar gases were supplied by Airgas. Ultra-pure water (resistivity:18.2 MΩ·cm at 25 °C, Milli-Q) was used for all solution and electrolyte preparation.

### Synthesis of pure δ-MnO_2_ and Cu-pi-δ-MnO_2_

All chemicals were purchased from Sigma-Aldrich and used without further purification. For the synthesis of δ- MnO_2_, 1.50 g of KMnO_4_ and 0.28 g of MnSO_4_·H_2_O were dissolved in 80 mL of deionized water under continuous magnetic stirring for 30 minutes. Then, the solution was transferred into a 100 mL Teflon-lined stainless-steel autoclave and heated at 160 °C for 12 h. After naturally cooling to room temperature (25 ± 2 °C), the obtained precipitate was collected by centrifugation at 5500 rpm ( ≈ 3000 g) and repeatedly washed with deionized water to remove residual impurities.

For the preparation of Cu-pi-δ-MnO_2_, the procedure was modified by first dissolving 1.50 g of KMnO_4_ and 0.28 g of MnSO_4_·H_2_O in 80 mL of deionized water under magnetic stirring for approximately 15 minutes to form a homogeneous suspension. Subsequently, 0.363 g of Cu(NO_3_)_2_·2.5H_2_O was introduced, and the mixture was stirred for an additional 15 minutes. All subsequent steps followed the same procedure as described above.

### Synthesis of 55 nm Au nanoparticles (NPs)

First, 200 mL of a 0.01 wt% HAuCl_4_·4H_2_O aqueous solution was placed in a 250 mL round-bottom flask and heated to boiling under continuous magnetic stirring. The reaction was conducted under reflux using a condenser in an oil bath at 140 °C under ambient air conditions, with the system open to the atmosphere. Upon reaching boiling, 1.5 mL of a 1 wt% trisodium citrate dihydrate solution was rapidly injected. The reaction mixture was maintained under reflux and continuous stirring for an additional 30 min, followed by natural cooling to room temperature.

### Synthesis of Core-Shell Au@2 nm δ-MnO_2_, Au@25 nm δ-MnO_2_ and Au@25 nm Cu-pi-δ-MnO_2_ NPs

Mechanistic analysis across different shell thicknesses enables us to decouple proton-dominated and cation-driven intercalation mechanisms

To synthesize Au@2 nm δ**-**MnO_2_, 10 mL of the previously synthesized Au NPs was placed in a 100 mL round-bottom flask. Then, 0.5 mL of 0.01 M KMnO_4_ and 2.5 mL of 0.01 M K_2_C_2_O_4_ or 0.001 M MnSO_4_ were added, and the mixture was stirred continuously while being ice-bathed for 1 h.

For the synthesis of Au@25 nm δ-MnO_2_, the procedure was identical to that for Au@2 nm δ-MnO_2_, with the exception that after the 1-h ice bath, the mixture was heated in a 60 °C water bath for 10 h.

To prepare Au@25 nm Cu-pi-δ-MnO_2_, the steps followed were the same as those for Au@25 nm δ-MnO_2_, but with the addition of 0.083 mL of 0.01 M Cu(NO_3_)_2_ during the synthesis.

All as-synthesized Au@δ-MnO_2_ suspensions were washed with deionized water, centrifuged, and concentrated to 0.5 mL in 1.5 mL centrifuge tubes for Raman and CV tests.

### Materials characterization

Selected area electron diffraction (SAED), high spatial resolution structural and chemical characterizations were performed on the probe-corrected ARM 200CF, which is operated at 200 keV. This microscope was equipped with dual silicon drift detectors (SDDs). For individual SDD, the area is 100 mm^2^ and the total collection solid angle for energy-dispersive spectroscopy (EDS) acquisition is around 1.5 sr. The convergence angle for scanning transmission electron microscopy (STEM) analysis is around 28 mrad. The collection angle for high-angle annular dark field (HAADF) imaging and annular bright field (ABF) imaging is 90-250 mrad and 11-22 mrad. X-ray photoelectron spectroscopy (XPS) measurements were performed using a NEXSA G2 system (Thermo Fisher Scientific). Survey and high-resolution spectra were acquired under ultrahigh vacuum conditions with a base pressure below 1 × 10^–9 ^mbar. The pass energy was set to 400 eV for survey scans and 20 eV for high-resolution spectra. All spectra were calibrated using the C 1 s peak at 284.8 eV as a reference. No background subtraction was applied, and peak positions were determined directly from the measured spectra. Data analysis was performed using Thermo Avantage software. X-ray diffraction (XRD) analysis was carried out using a Smartlab 3 kW Gen2 system. In situ Raman spectroscopy was carried out with a confocal microscope Raman system (RENISHAW).

ICP-OES measurements were performed using a Thermo Scientific iCAP 7400 system. For Au-containing samples, 10 mL of the as-synthesized Au@δ-MnO_2_ or Au@Cu-pi-δ-MnO_2_ nanoparticle suspension was centrifuged and concentrated to approximately 1 mL. The concentrated suspension was digested in freshly prepared aqua regia (HCl:HNO_3_ = 3:1, v/v) at room temperature for 24 h to ensure complete dissolution of both the Au core and the metal oxide shell. The resulting solution was diluted with deionized water to an elemental concentration of approximately 5 mg L^-1^ prior to ICP-OES analysis.

For Au-free samples, approximately 2.0 mg of δ-MnO_2_ or Cu-pi-δ-MnO_2_ powder was digested in 1 mL of freshly prepared aqua regia (HCl:HNO_3_ = 3:1, v/v) until complete dissolution. The digested solution was then diluted with deionized water to a final volume of 250 mL, giving an approximate Mn concentration of 5 mg L^–1^. For Cu-pi-δ-MnO₂ samples, both Mn and Cu contents were quantified from the same diluted solution. Calibration was performed using standard solutions with known concentrations.

### Electrochemical characterization

The electrochemical behavior was studied in a sealed electrolytic cell (100 mL) at room temperature using a three-electrode configuration, with a Pt counter electrode and a Hg/HgO reference electrode (saturated with 1 M KOH). To prepare the working electrode, a 10 µL aliquot of the suspension (in DI water) was drop-cast onto a plasma-treated glass carbon disk electrode (3 mm in diameter, SGC-certified, GDMS-tested) and dried under ambient conditions. The working electrode for cyclic voltammetry (CV) contained only the active material, whereas that for galvanostatic charge-discharge (GCD) additionally incorporated carboxylic acid-functionalized multi-walled CNTs (10 wt%) and poly(acrylic acid) (M_v_ ~450,000, 10 wt%) to enhance operational stability. The temperature during the measurements was maintained at 25 ± 2 °C.

For all working electrodes, the loading mass of active materials (δ-MnO_2_ or Cu-pi-δ-MnO_2_, excluding Au content) was adjusted to 1 µg by controlling the suspension concentration. All potentials were referenced to the reversible hydrogen electrode (RHE), which was calibrated against the hydrogen oxidation/evolution equilibrium in an H_2_-saturated electrolyte using a Pt electrode. The cell contained 60 mL of argon-saturated KOH solution with varying concentrations. CV measurements were carried out at a scan rate of 50 mV s^–1^ within varying potential windows, while GCD measurements were conducted at a specific current of 20 A g^–1^ over the potential range of 0 to 1.2 V vs. RHE.

The specific capacitance (*C*) values were calculated from the GCD curves according to the equation: $C=\left(I\Delta t\right)/\left(m\Delta V\right)$, where I, Δt, m, and ΔV indicate the discharge current (A), discharge time (s), and active material mass (g), and operating potential window (V), respectively. In addition, for electrodes with an Au core (Au@2 nm MnO_2_ and Au@25 nm MnO_2_), the capacitance contribution from the Au core was subtracted to clearly demonstrate the capacitance originating from MnO_2_.

At the same amount of active MnO_2_ (1 µg), the ICP-OES analysis revealed that Au@25 nm MnO_2_ and Au@2 nm MnO_2_ had Mn/Au mass ratios of 1:21 and 1:96, respectively. As a result, the capacitance contributions from the Au core for Au@25 nm MnO_2_ and Au@2 nm MnO_2_ were measured to be 104.6 and 428.3 F g^-1^, respectively, through GCD measurements of Au NP electrodes under identical conditions (i.e., same loading mass of Au core and specific current).

### In situ electrochemical surface-enhanced Raman spectroscopy study

Here, we employ in situ Raman spectroscopy in conjunction with electrochemical measurements to demonstrate that metal pre-intercalation promotes a proton-coupled charge-storage pathway, enabling its direct molecular-level visualization. Electrochemical Raman measurements were performed using a confocal microscope Raman system (RENISHAW) with a 633 nm He-Ne laser as the excitation source. A 50× microscope objective (numerical aperture 0.55) was used for all measurements. To ensure accuracy, Raman frequencies were calibrated using the instrument’s built-in silicon reference.

In situ electrochemical Raman experiments were conducted in a custom-designed spectroelectrochemical cell using a three-electrode configuration, with a glassy carbon working electrode, a Pt counter electrode, and an Hg/HgO reference electrode (1 M KOH). The working electrode was prepared following the same procedure as for the electrochemical measurements. The electrolyte consisted of KOH solutions with varying concentrations, as specified in each experiment. The amount of solution used each time in the self-made cell is approximately 10 mL. The cell was thoroughly cleaned by soaking in concentrated sulfuric acid for 12 h prior to use.

In situ spectroelectrochemical measurements are generally conducted under either potentiostatic or galvanostatic control. In this work, all in situ electrochemical SERS measurements were performed under potentiostatic control at a temperature of 25 ± 2 °C. The potential was stepped from 1.1 V (vs. RHE) in 100 mV intervals, and at each potential, the system was allowed to stabilize for 30 s before spectral acquisition.

Raman spectra were collected using a 633 nm laser at 5% power with an acquisition time of 50 s per spectrum. Each spectrum was recorded three times and averaged to ensure reproducibility.

### Computational details

#### Model construction

The initial structure of K^+^-intercalated MnO_2_ was based on the composition K_0.125_MnO_2_·0.25H_2_O, as reported previously^4^. This model was re-optimized in the present study using the DFT settings outlined below. The resulting interlayer spacing (7.18 Å) is in close agreement with the literature value (7.10 Å). The Cu_0.125_MnO_2_·0.25H_2_O structure was constructed by substituting K^+^ with Cu^2+^, in approximate accordance with the K^+^/Cu^2+^ ratio of 1:0.8 determined from inductively coupled plasma (ICP) analysis.

#### DFT calculations

All structural optimizations and electronic structure calculations were carried out using spin-polarized density functional theory (DFT) as implemented in the Vienna Ab Initio Simulation Package (VASP)^31^. The projector augmented-wave (PAW) method^32^ and the Perdew–Burke–Ernzerhof (PBE) exchange-correlation functional^33^ were employed. A plane-wave energy cutoff of 500 eV was applied, and the convergence criteria were set to 0.01 eV·Å^–1^ for forces and 10^–5 ^eV for total energy. Brillouin zone sampling was performed using a Γ-centered 1×2×2 k-point mesh for the K_0.125_MnO_2_·0.25H_2_O and Cu_0.125_MnO_2_·0.25H_2_O models. Long-range van der Waals interactions were accounted for using the DFT-D3(BJ) dispersion correction^34^. Transition-state searches were conducted using the climbing-image nudged elastic band (CI-NEB) method^35^. The Vaspsol implicit solvation model was employed to determine the energy profiles for cation and water intercalation processes^36,37^.

## Supplementary information


Supplementary Information
Description of Additional Supplementary Files
Supplementary Data 1
Supplementary Data 2
Supplementary Data 3
Supplementary Data 4
Transparent Peer Review file


## Source data


Source Data


## Data Availability

The data that support the findings of this study are available in the paper and its Supplementary Information. Source data are provided with this paper.
