# Supplementary material for: Metal pre-intercalation promotes water-mediated proton-coupled electron transfer in layered δ-MnO2 for aqueous pseudocapacitive energy storage
Source: Nat Commun. 2026 Jul 28;17:9168. doi: 10.1038/s41467-026-76022-4 (PMC13522339; doi:10.1038/s41467-026-76022-4)
Supplement: Supplementary file 1 [file 41467_2026_76022_MOESM1_ESM.pdf]

Metal Pre-Intercalation Promotes Water-Mediated Proton-Coupled Electron Transfer in Layered δ-MnO_2 for Aqueous Pseudocapacitive Energy Storage

Huajie Ze[1,2†], Yongkwon Song[3†], Xijun Wang[4], Weiyan Ni[1,2], Xiaobing Hu[3], Jianan Erick Huang[1,2], Zeyan Liu[1,2], Hengzhou Liu[1,2], Xiao-Yan Li[1,2], Randall Q. Snurr[4], Mark C. Hersam[1,2,3*], Ke Xie[1,2*], Edward H. Sargent[1,2,3*]

Affiliations:

[1]Department of Chemistry, Northwestern University, 2145 Sheridan Road, Evanston, IL 60208, USA

[2]Department of Electrical and Computer Engineering, Northwestern University, 2145 Sheridan Rd, Evanston, IL 60208, USA

[3]Department of Materials Science and Engineering, Northwestern University, Evanston, IL 60208, USA

[4]Department of Chemical and Biological Engineering, Northwestern University, Evanston, IL 60208, USA

[†]These authors contributed equally.

Corresponding authors: ted.sargent@northwestern.edu; ke-xie@northwestern.edu;

m-hersam@northwestern.edu

Table of Contents

Supplementary Table 1 4
Supplementary Table 2 4
Supplementary Figure 1 5
Supplementary Figure 2 5
Supplementary Figure 3 6
Supplementary Figure 4 6
Supplementary Figure 5 7
Supplementary Figure 6 8
Supplementary Figure 7 9
Supplementary Figure 8 10
Supplementary Figure 9 11
Supplementary Figure 10 12
Supplementary Figure 11 13
Supplementary Figure 12 14
Supplementary Figure 13 15
Supplementary Figure 14 16
Supplementary Figure 15 17
Supplementary Figure 16 18
Supplementary Figure 17 19
Supplementary Figure 18 20
Supplementary Figure 19 21
Supplementary Figure 20 22
Supplementary Figure 21 23
Supplementary Figure 22 24
Supplementary Figure 23 25
Supplementary Figure 24 26
Supplementary Figure 25 27

Supplementary Figure 26 28
Supplementary Figure 27 29
Supplementary Figure 28 30
Supplementary Figure 29 31
Supplementary Figure 30 32
Supplementary Figure 31 33
Reference 34

Supplementary Table 1. Inductively coupled plasma-optical emission spectroscopy (ICP-OES) analysis of δ-MnO_2 and Cu-pi-δ-MnO_2.

	K/Mn Ratio	Cu/Mn Ratio
δ-MnO_2	~0.125	0
Cu-pi-δ-MnO_2	~0.009	~0.089

Supplementary Table 2. Capacitance contribution of Au NPs in Au@2 nm MnO_2 and Au@25 nm MnO_2 when measuring the discharge/charge capacity of Au@25 nm MnO_2 and Au@25 nm Cu-pi-MnO_2 with the same Mn mass.

	ICP-OES Results: Mn/Au Mass Ratio	Capacitance contribution of Au NPs
Au@2 nm MnO_2	~1:96	~428.3 F/g
Au@25 nm MnO_2	~1:21	~104.6 F/g
Au@25 nm Cu-pi-MnO_2	~1:21	~104.6 F/g

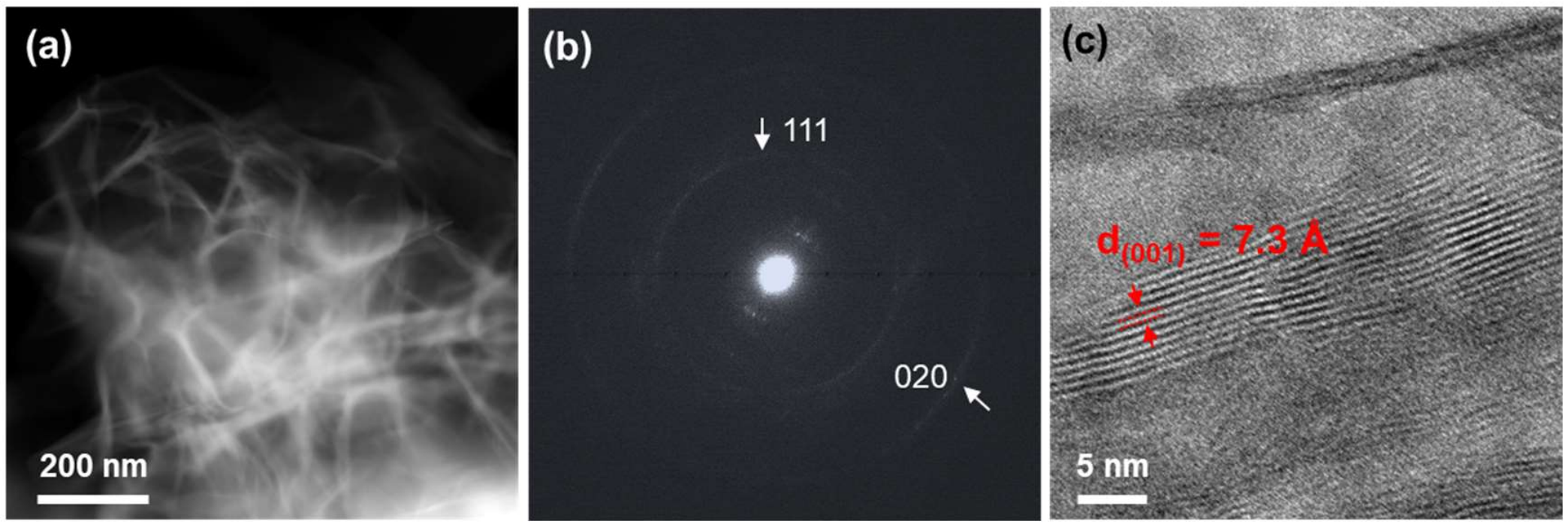

Supplementary Figure 1| a-c, HAADF, SAED patterns and HRTEM image of synthesized δ-MnO_2

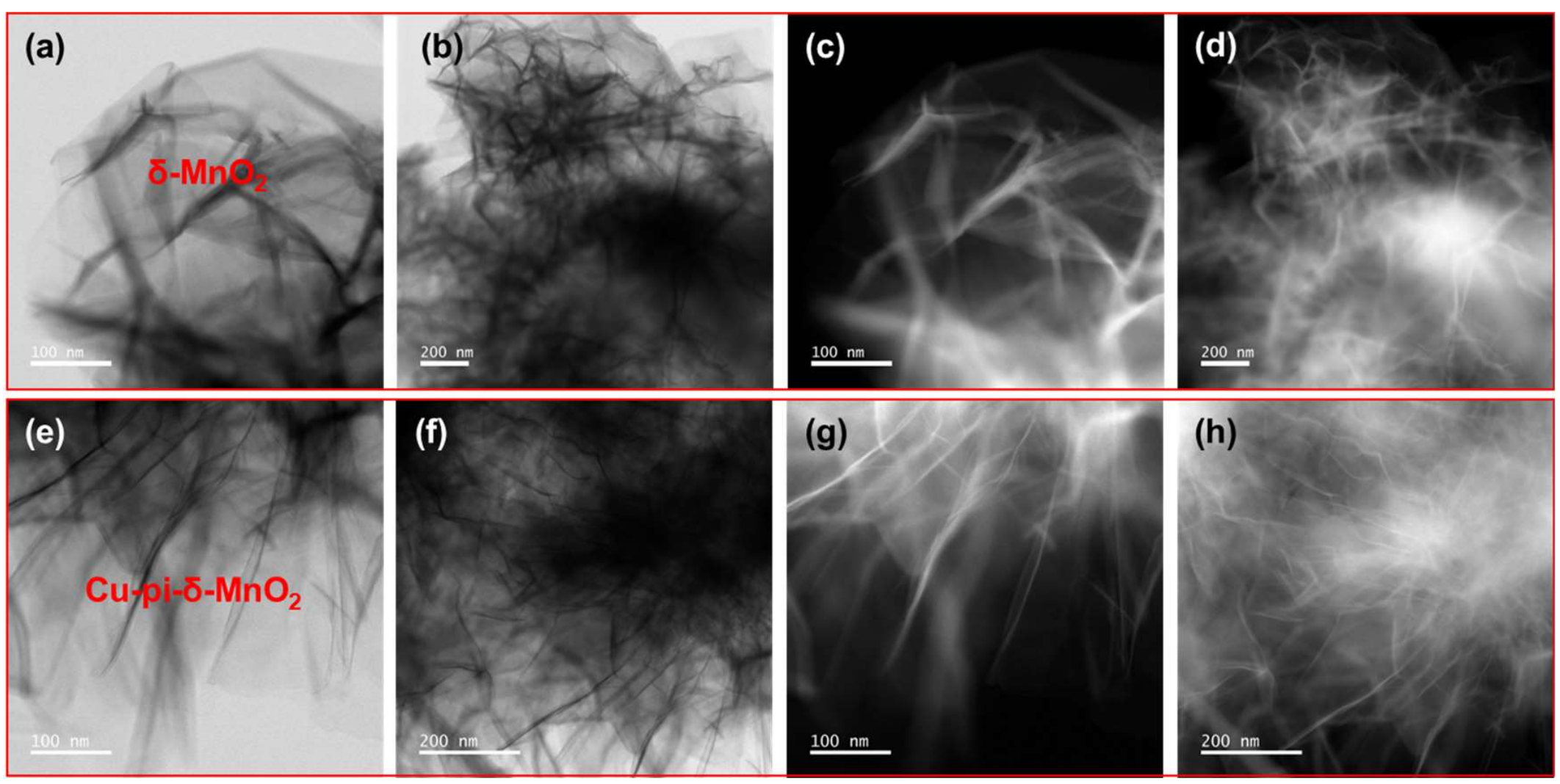

Supplementary Figure 2| a-d, Annular bright field (ABF) images (a,b) and the corresponding high-angle annular dark field (HAADF) images (c,d) of synthesized δ-MnO_2; **e-h**, Annular bright field (ABF) images (e,f) and the corresponding high-angle annular dark field (HAADF) images (g,h) of synthesized Cu-pi-δ-MnO_2;

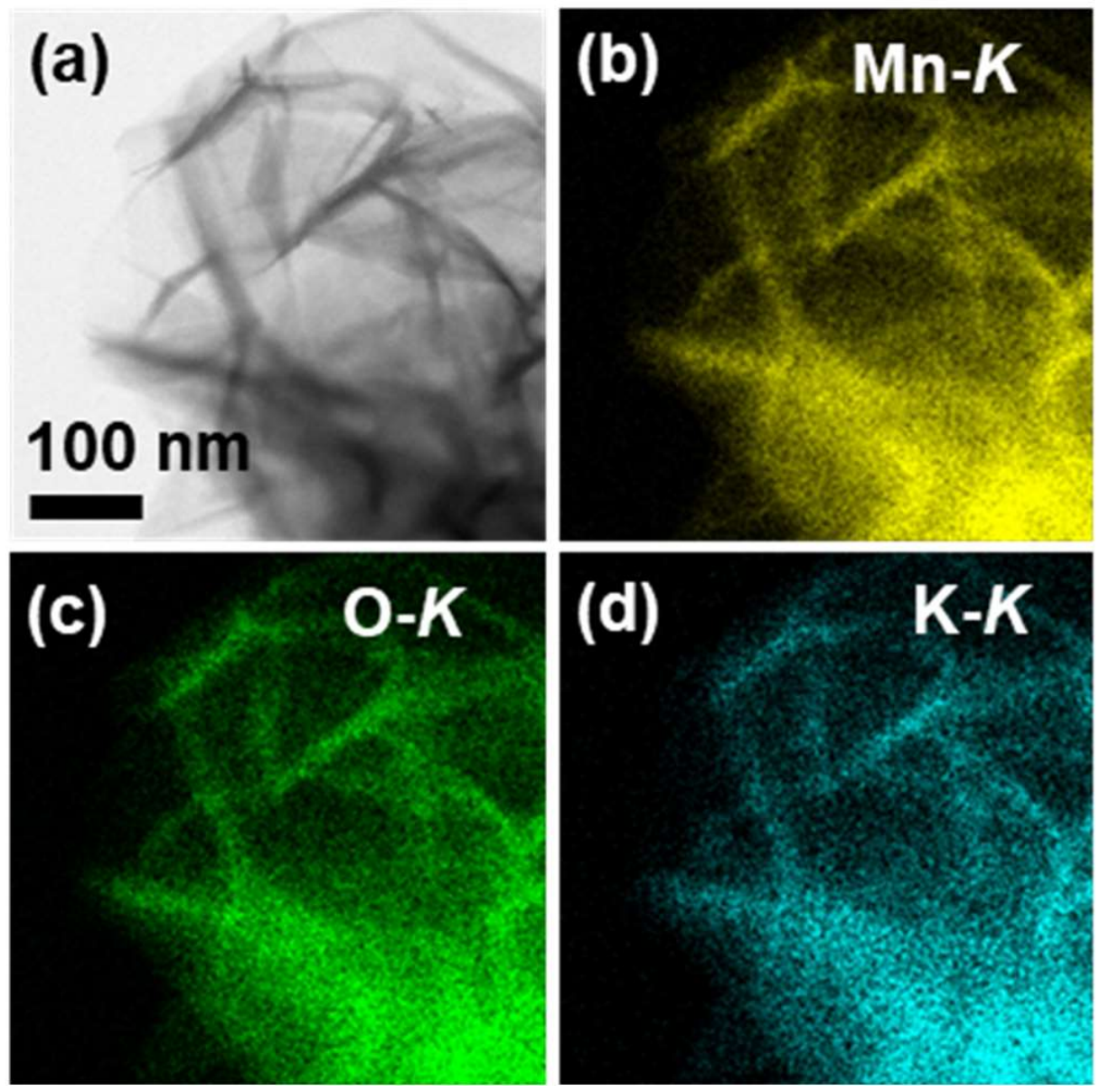

Supplementary Figure 3| a, ABF image of Cu-pi-δ-MnO_2. **b-d**, the corresponding EDS mapping of Mn (b), O (c), K (d).

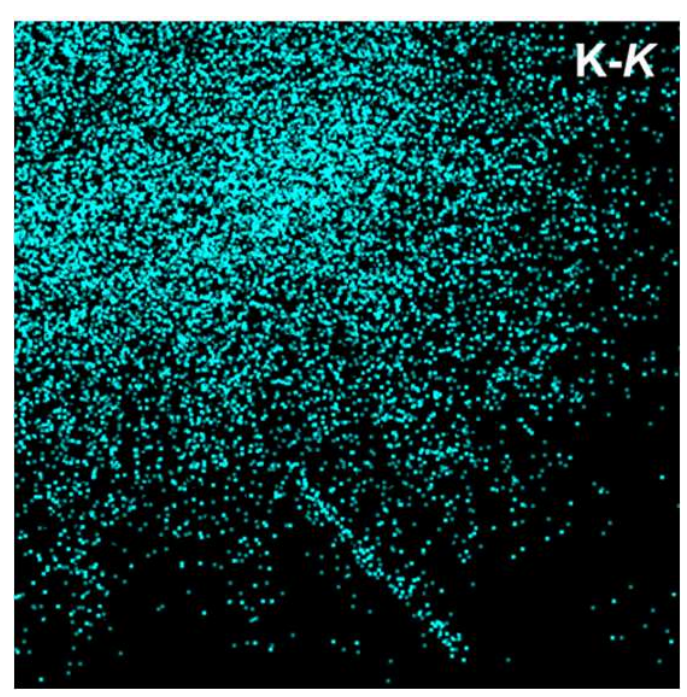

Supplementary Figure 4| Energy-dispersive spectroscopy (EDS) elemental map of K in Cu-pi-δ-MnO_2. The results reveal the presence of K in the Cu-pi-δ-MnO_2 sample, indicating the coexistence of K and Cu within the structure.

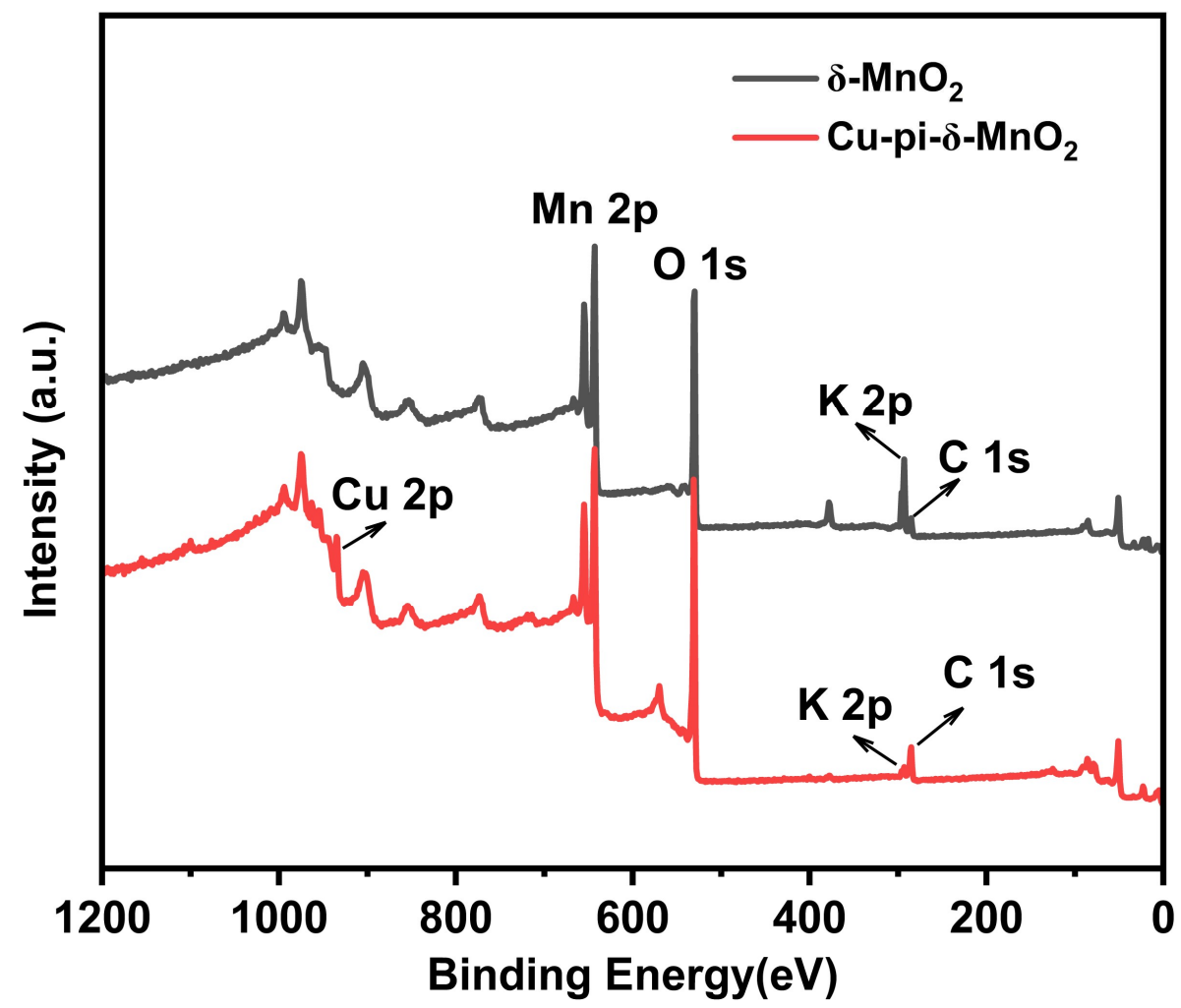

Supplementary Figure 5| XPS fully scanned spectrum of δ-MnO_2 and Cu-pi-δ-MnO_2.

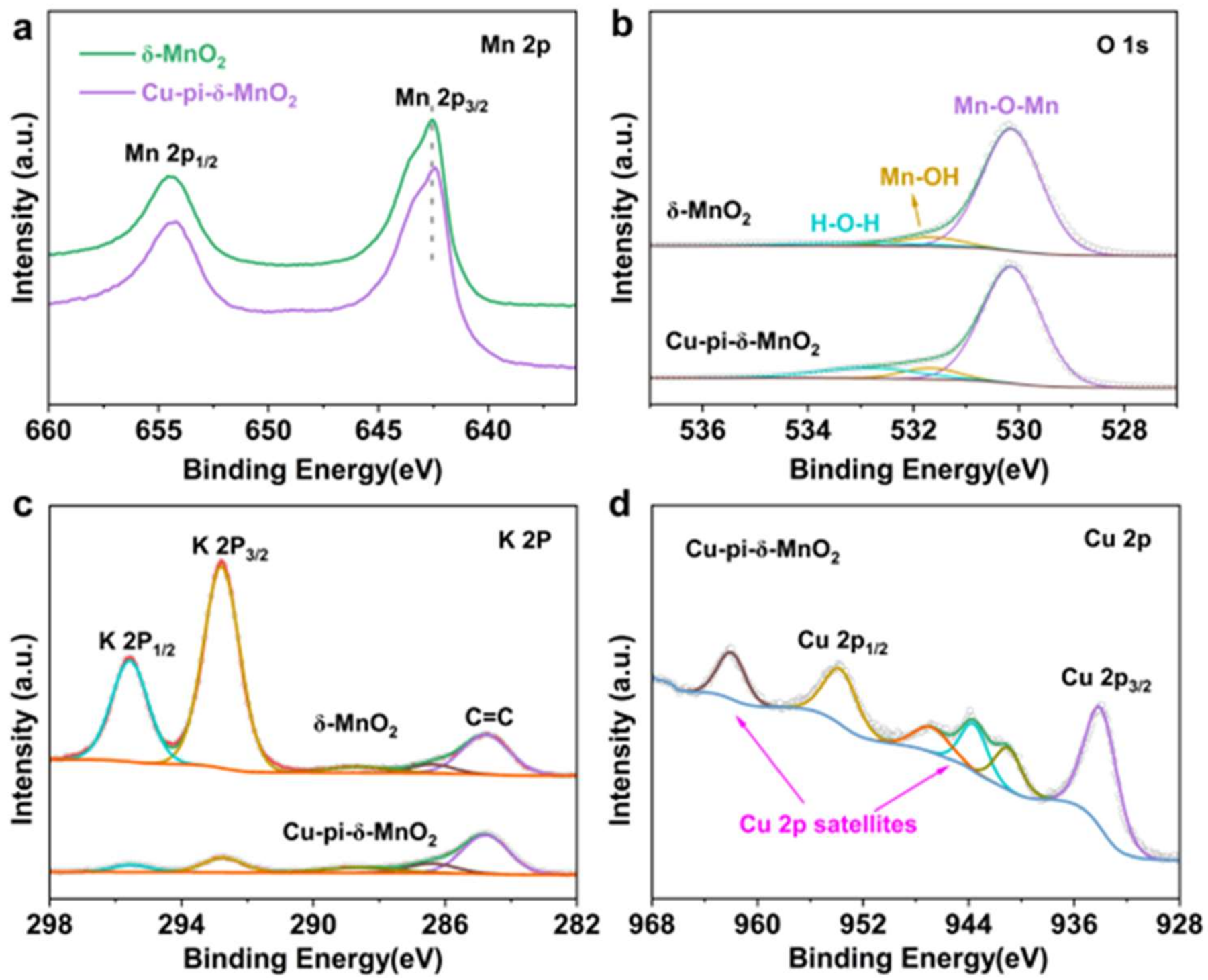

Supplementary Figure 6| XPS spectra of Mn 2*p* (a), O 1*s* (b), K 2*p* (c) and Cu 2*p* (d) for δ-MnO_2 and Cu-pi-δ-MnO_2. The Mn 2*p* spectra confirm Mn^{4+} as the dominant oxidation state in both samples, while Cu-pi-δ-MnO_2 exhibits a slight shift toward lower binding energy, indicating a partial reduction of Mn^{4+} upon Cu^{2+} pre-intercalation.[1] This valence adjustment provides an effective pathway for compensating the excess positive charge introduced by divalent Cu^{2+} ions. The O 1*s* XPS spectra can be deconvoluted into three peaks located at 530.2, 531.7, and 532.9 eV, corresponding to Mn-O-Mn, Mn-OH, and H-O-H bonds, respectively.[1-4] These peaks reflect distinct oxygen species: lattice oxygen (Mn-O-Mn and Mn-OH) at lower binding energies, and adsorbed oxygen (H-O-H) at higher binding energy. Compared to pristine δ-MnO_2, Cu-pi-δ-MnO_2 shows an increased fraction of Mn-OH and H-O-H components, indicative of enhanced interlayer water retention and hydroxyl formation.

The partial reduction of Mn^{4+} and the increased population of interlayer OH/H_2O species cooperatively compensate for the additional charge associated with Cu^{2+} pre-intercalation, ensuring overall charge neutrality of the δ-MnO_2 lattice without requiring anion incorporation or defect formation.

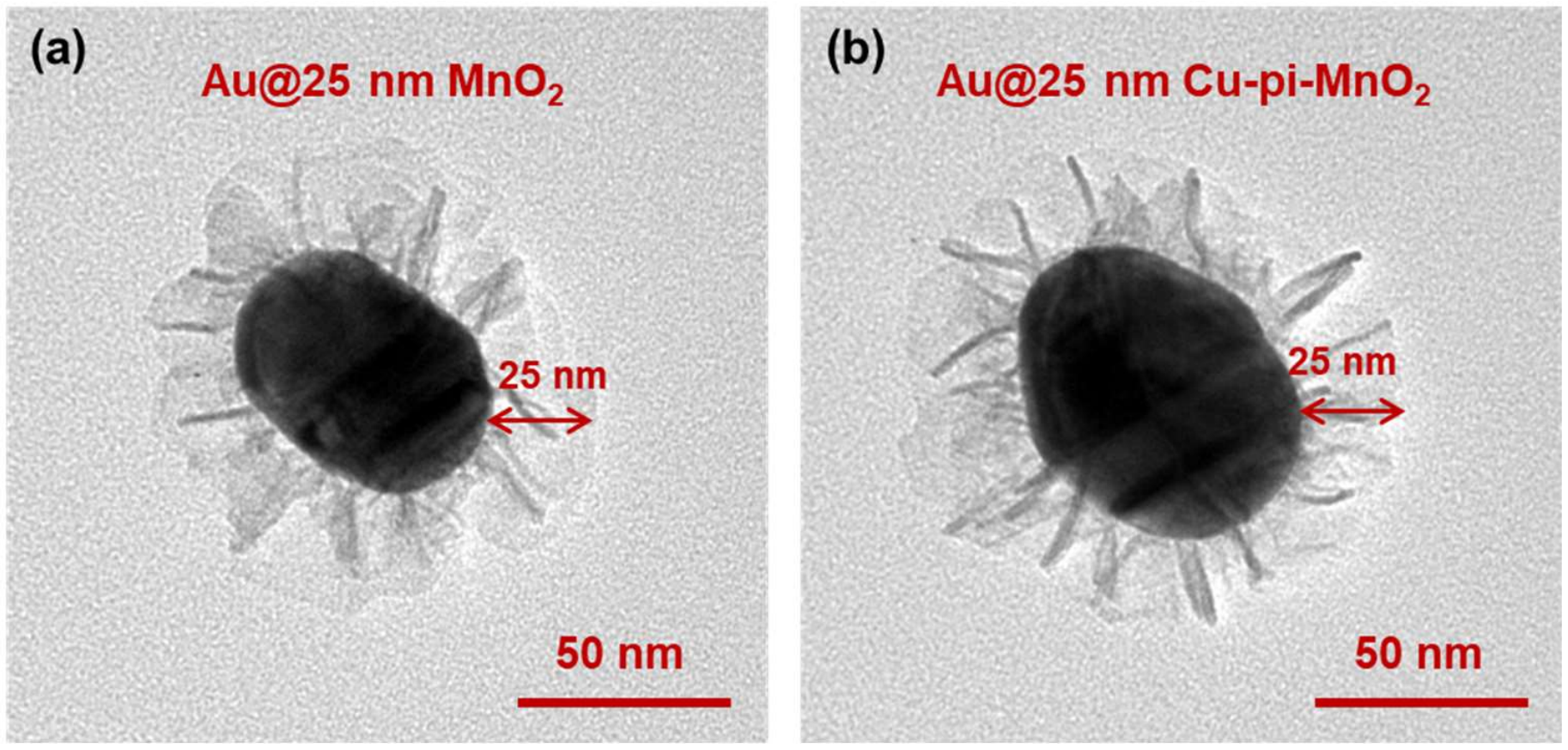

Supplementary Figure 7| a-b, Transmission electron microscopy (TEM) images of Au@25 nm MnO_2 (a) and Au@25 nm Cu-pi-MnO_2 (b) nanoparticles.

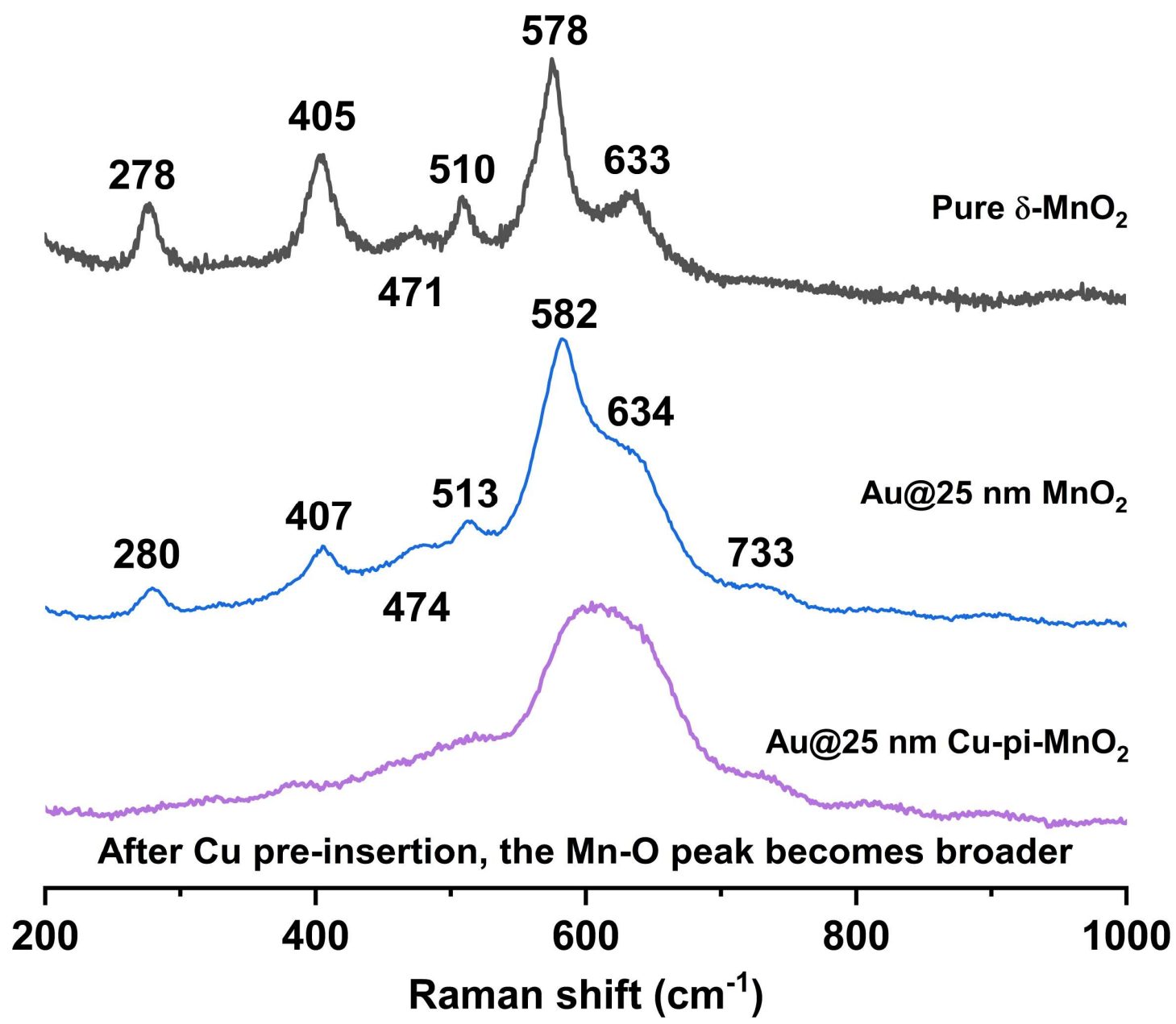

Supplementary Figure 8| Raman spectra acquired from pure δ-MnO_2, Au@25 nm MnO_2, and Au@25 nm Cu-pi-MnO_2 samples shows characteristic vibrational signatures of the MnO_2 lattice. The spectral region spanning 200-700 cm^{-1} encompasses the fundamental lattice modes of MnO_2. In particular, the ν_1 mode appearing at 620-650 cm^{-1} arises from the symmetric stretching of Mn-O bonds within MnO_6 octahedra, whereas the ν_2 mode, located between 570 and 590 cm^{-1}, corresponds to Mn-O vibrations along the interconnected octahedral chains that constitute the MnO_2 framework.[5-7]

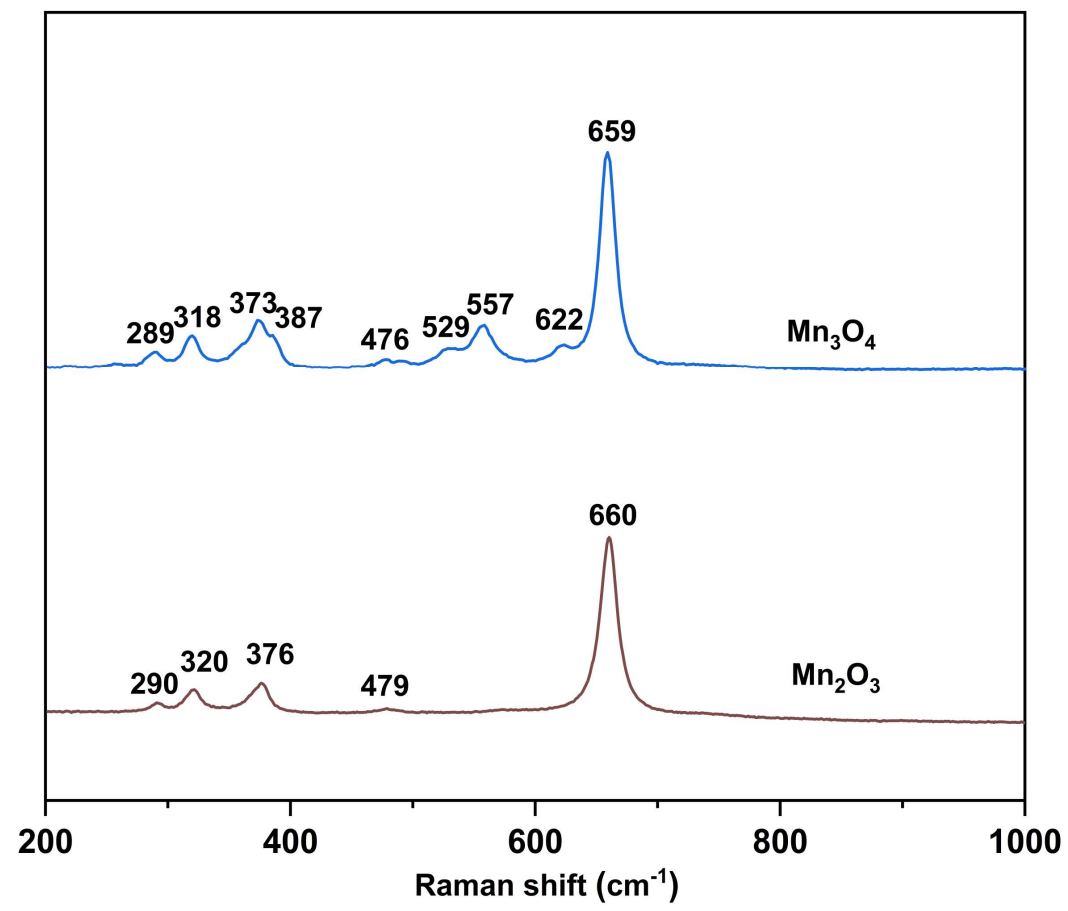

Supplementary Figure 9| Raman spectra of commercial Mn_2O_3 and Mn_3O_4 powders reveal characteristic features of their respective manganese oxidation states. For Mn_3O_4, which contains both Mn^{3+} and Mn^{2+} species, the prominent peak at ~659-660 cm^{-1} is attributed to Mn(III)-O vibrations, while the bands observed around ~622 and ~557 cm^{-1} in Fig. 4b, c correspond to Mn(II)-O bonding modes.

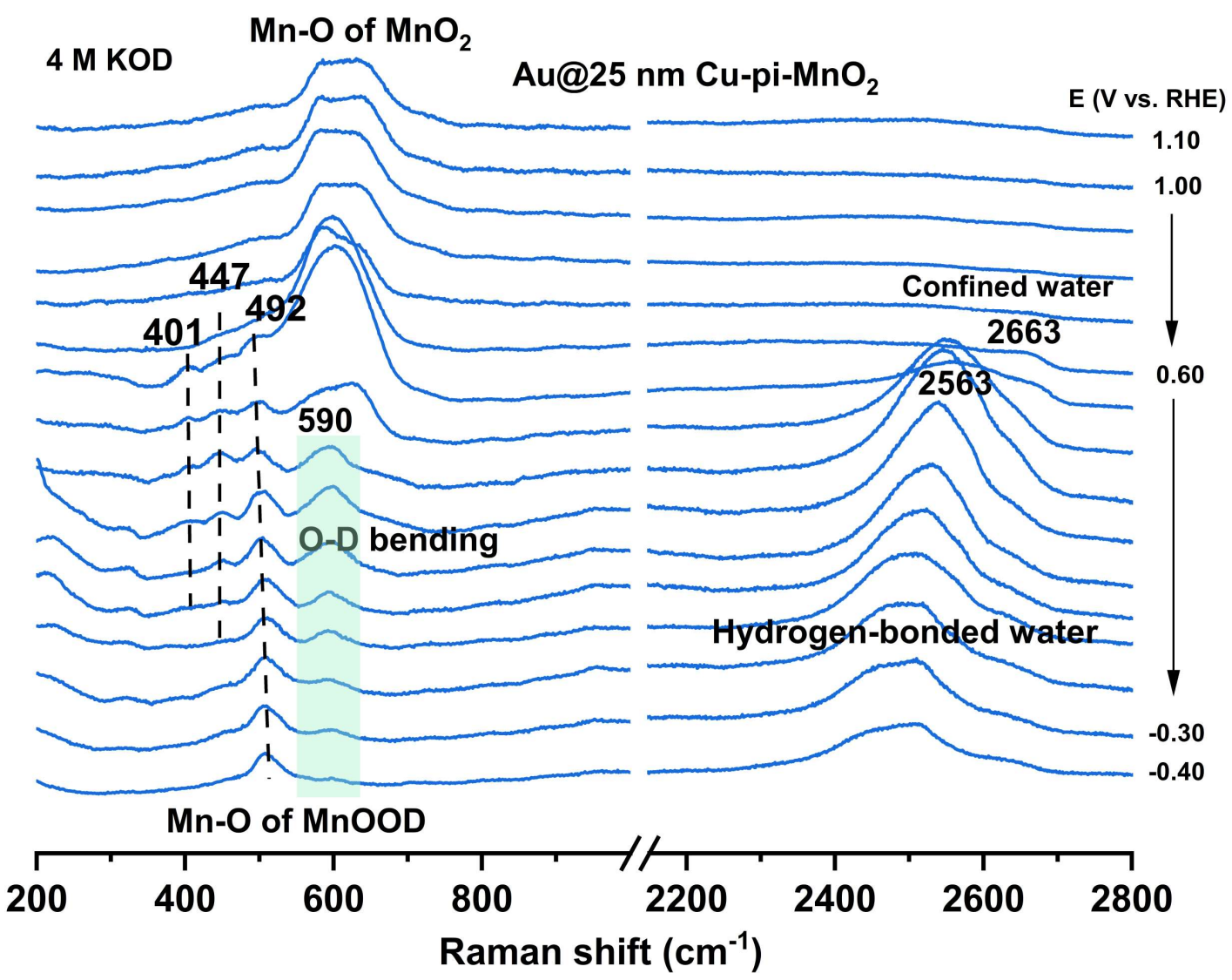

Supplementary Figure 10| In situ electrochemical-SERS investigation of the discharge process on Au@25 nm Cu-pi-MnO_2 in 4 M KOD electrolyte. The Mn-O bonds (~413, ~461, ~504 cm^{-1}) in MnOOH exhibit slight shifts (~401, ~447, ~492 cm^{-1}) in D_2O experiment. This shift arises from the structural transformation from H-O-H···O=Mn-O-H in regular water to D-O-D···O=Mn-O-D in the D_2O, which modifies the Mn-O bonding environment.

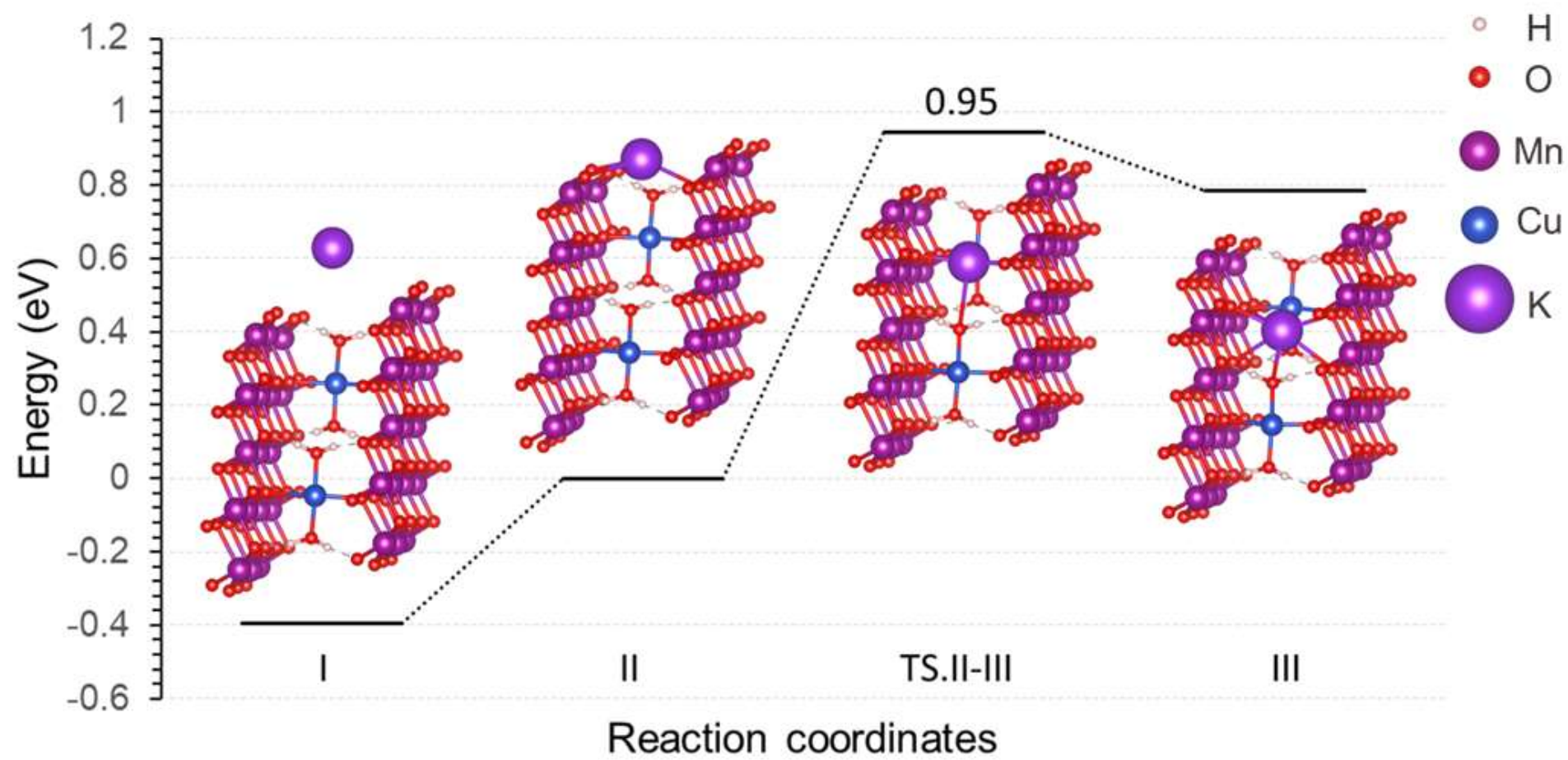

Supplementary Figure 11| DFT-simulated energy profiles for the interlayer migration of K^+ in Cu-pi-MnO_2.

As shown in **Supplementary Figure 11**, the migration pathway of K^+ from the external solution into the Cu-pi-MnO_2 interlayer involves two distinct steps. First, K^+ binds to an edge O site on the MnO_2 framework (I → II), which is an endothermic process with an energy increase of 0.40 eV. Subsequently, K^+ migrates from this edge site to a nearby inner site, forming additional coordination bonds with surrounding O atoms (II → III). This step proceeds through a transition state (TS) with an energy barrier of 0.95 eV.

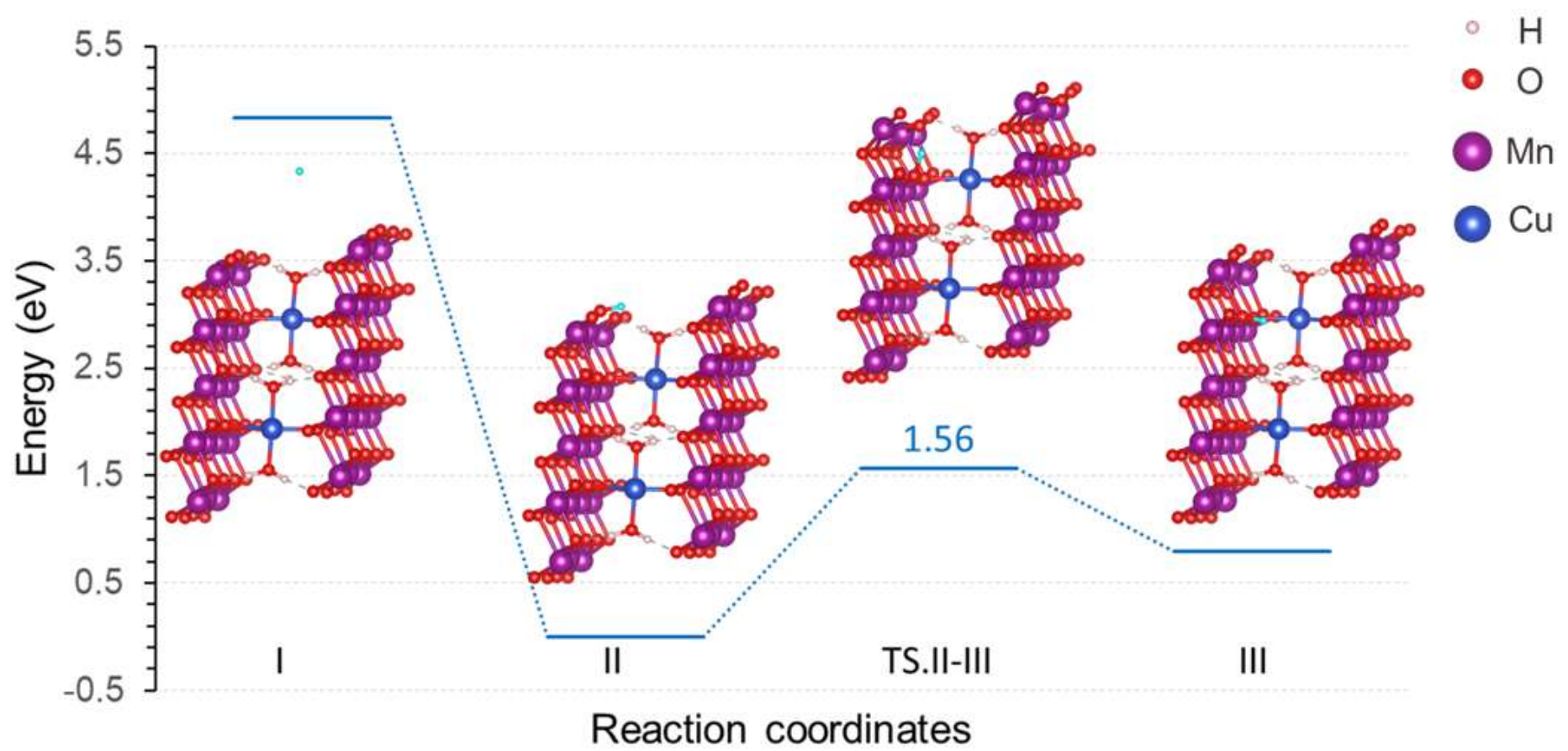

Supplementary Figure 12| DFT-simulated energy profiles for the interlayer migration of H^+ in Cu-pi-MnO_2.

Similarly, the proton migration pathway was examined, as depicted in **Supplementary Figure 12**. The initial adsorption of H^+ onto an edge O site from the bulk solution (I → II) is found to be highly exothermic (-4.83 eV). This large exothermicity likely arises from the artificial destabilization of a free proton in the implicit solvent model; notably, if an explicit solvent model were employed, wherein H^+ is stabilized by a hydrogen-bonding network of surrounding H_2O molecules, we expect the proton would be more stabilized in the solution phase, rendering its adsorption onto the MnO_2 edge O site less exothermic. Once bound to the edge O site, direct proton hopping to an adjacent lattice O site requires overcoming a prohibitive activation barrier of 1.564 eV, likely due to the strength of the O-H bond that must be broken.

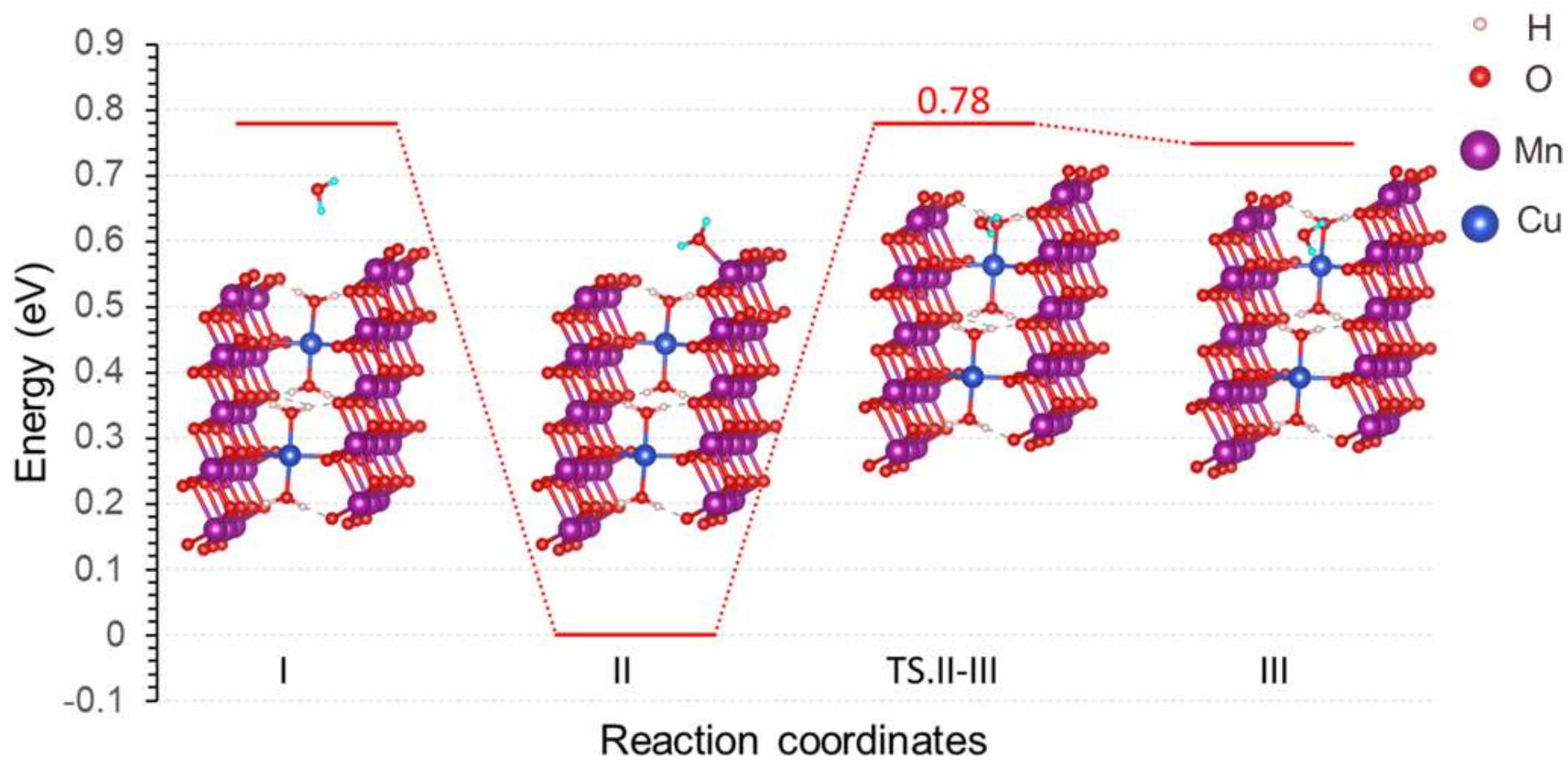

Supplementary Figure 13| DFT-simulated energy profiles for the interlayer migration of H_2O in Cu-pi-MnO_2.

Additionally, the intercalation of water molecules themselves was explored (**Supplementary Figure 13**). H_2O readily adsorbs at open Mn sites at the MnO_2 edge (I → II), stabilizing the system by 0.78 eV. Further migration into the deeper interlayer region requires breaking the H_2O-Mn coordination bond and overcoming an activation barrier of 0.78 eV to proceed inward.

These results collectively demonstrate that while bare proton migration faces substantial kinetic barriers, interlayer water molecules can effectively mediate proton transport, lowering the barrier and facilitating proton conduction within Cu-pi-MnO_2.

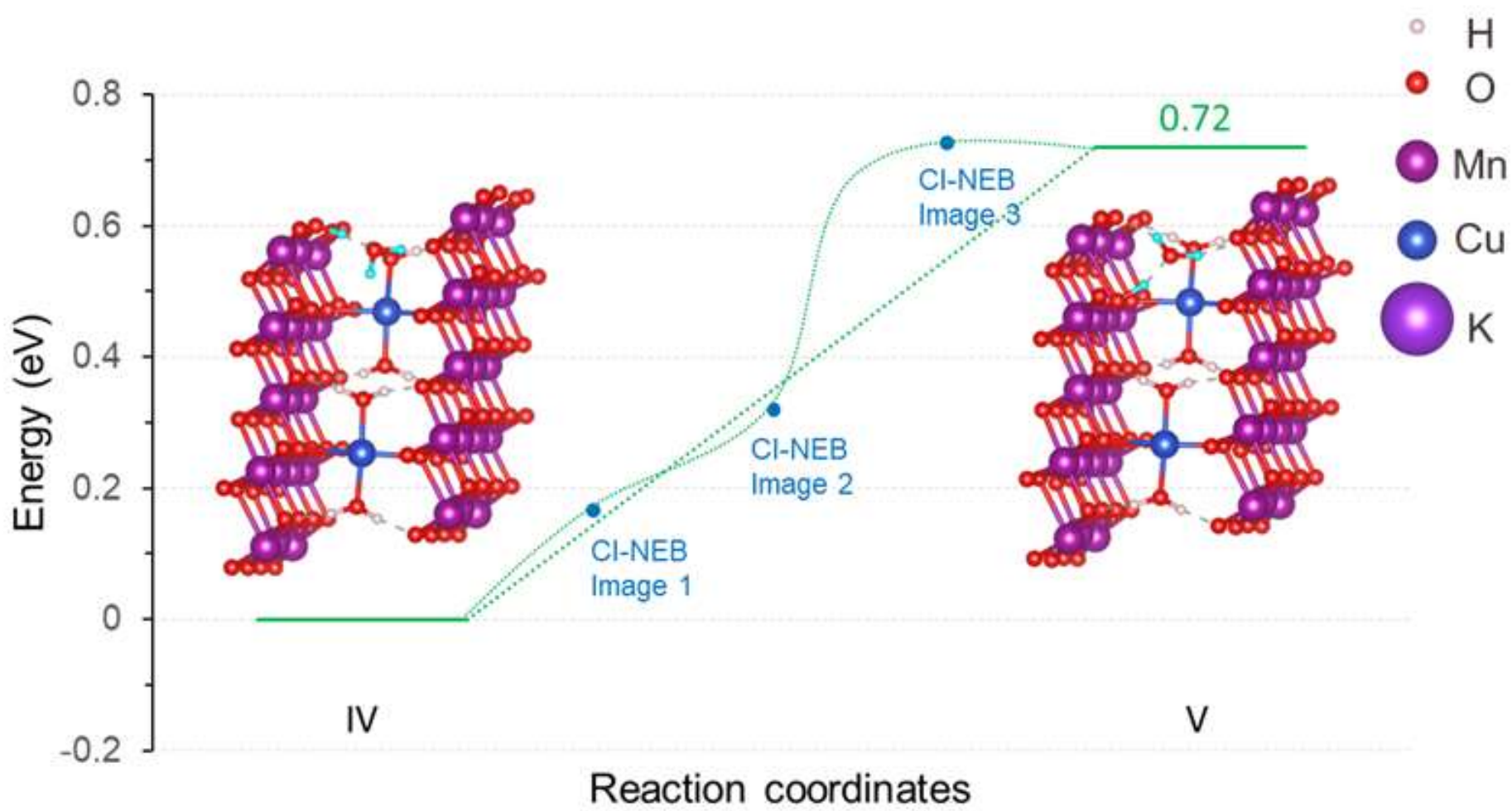

Supplementary Figure 14| DFT-simulated energy profiles for the interlayer migration of H^+ in Cu-pi-MnO_2, illustrating the effect of a neighboring H_2O molecule in facilitating proton transfer.

To assess the role of water-mediated proton transport, a proton relay mechanism was modeled, as illustrated in **Supplementary Figure 14** and Figure 2h of the main text. In this pathway, a proton in MnOOH attacks a neighboring absorbed water molecule, inducing proton transfer through the water network to a nearby lattice O site. This relay significantly lowers the effective migration barrier. Specifically, the energy required for proton transfer from an edge O site to a neighboring site (IV → V) drops to 0.72 eV. Climbing-image nudged elastic band (CI-NEB) calculations were employed but did not locate a distinct transition state, suggesting a low-barrier concerted mechanism.

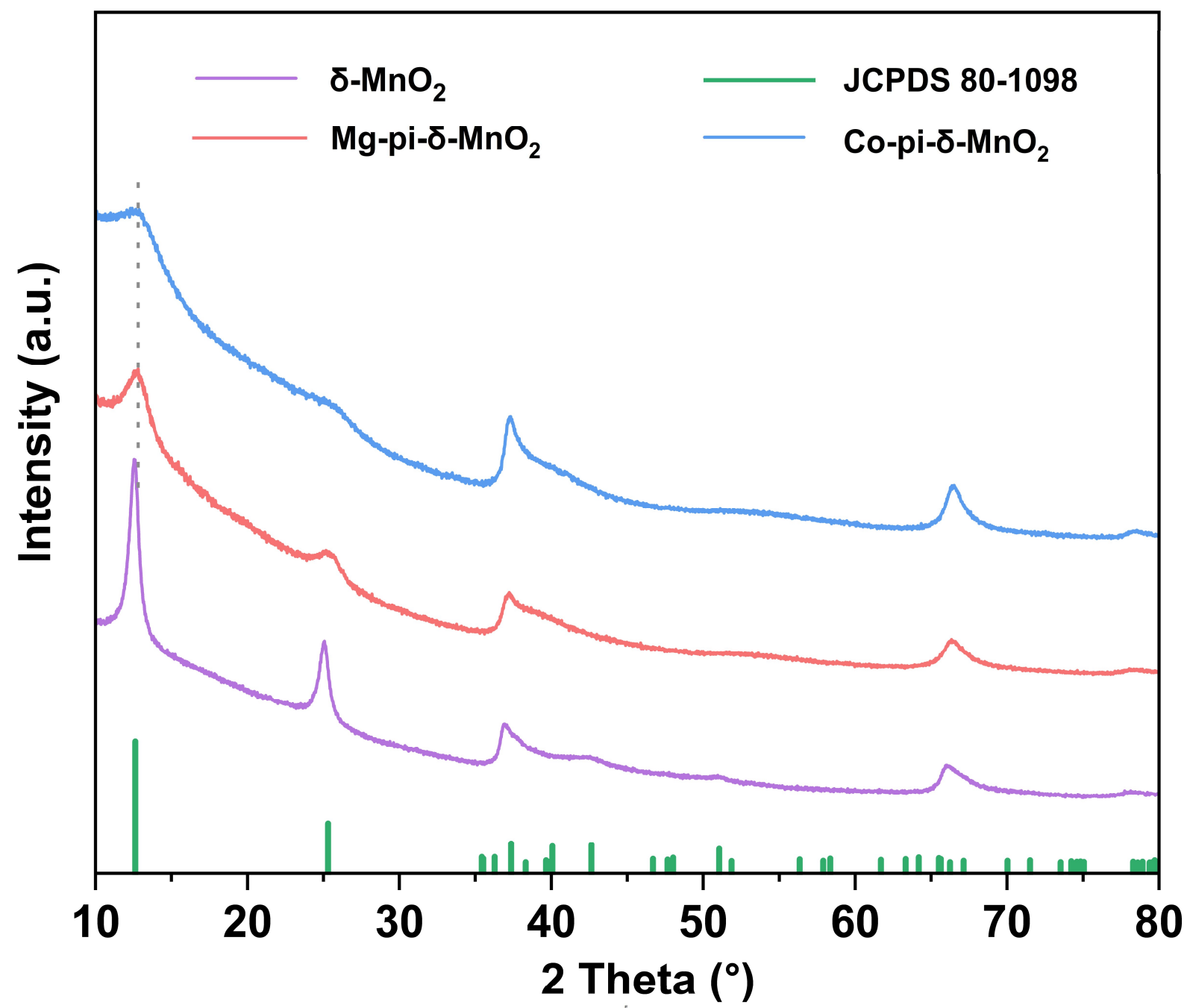

Supplementary Figure 15| XRD analysis of δ-MnO_2, Mg-pi-δ-MnO_2 and Co-pi-δ-MnO_2.

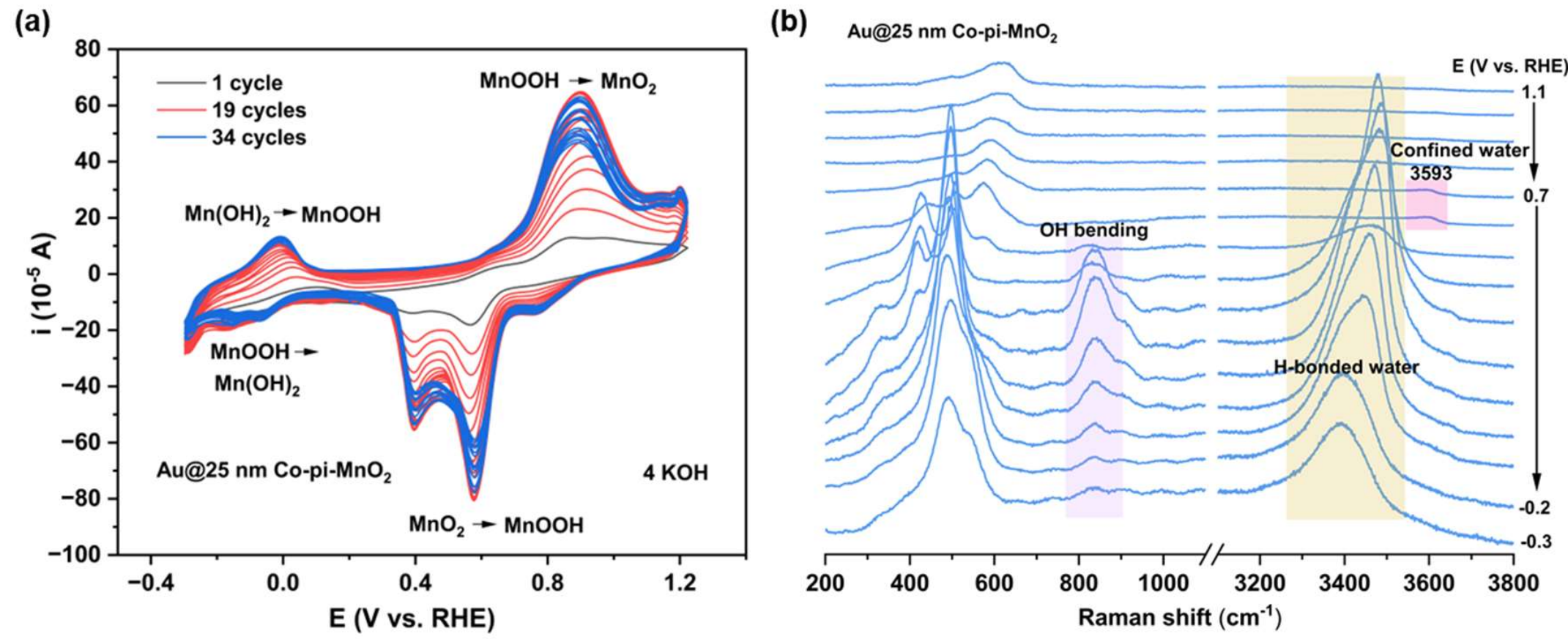

Supplementary Figure 16| a, Cyclic voltammetry (CV) curves of Au@25 nm Co-pi-MnO_2 collected in 4 M KOH at a scan rate of 50 mV s^{-1} within a potential window of -0.40 to 1.20 V vs. RHE over different cycles. **b**, Corresponding in situ electrochemical SERS investigation of the discharge process on Au@25 nm Co-pi-MnO_2 in 4 M KOH electrolyte.

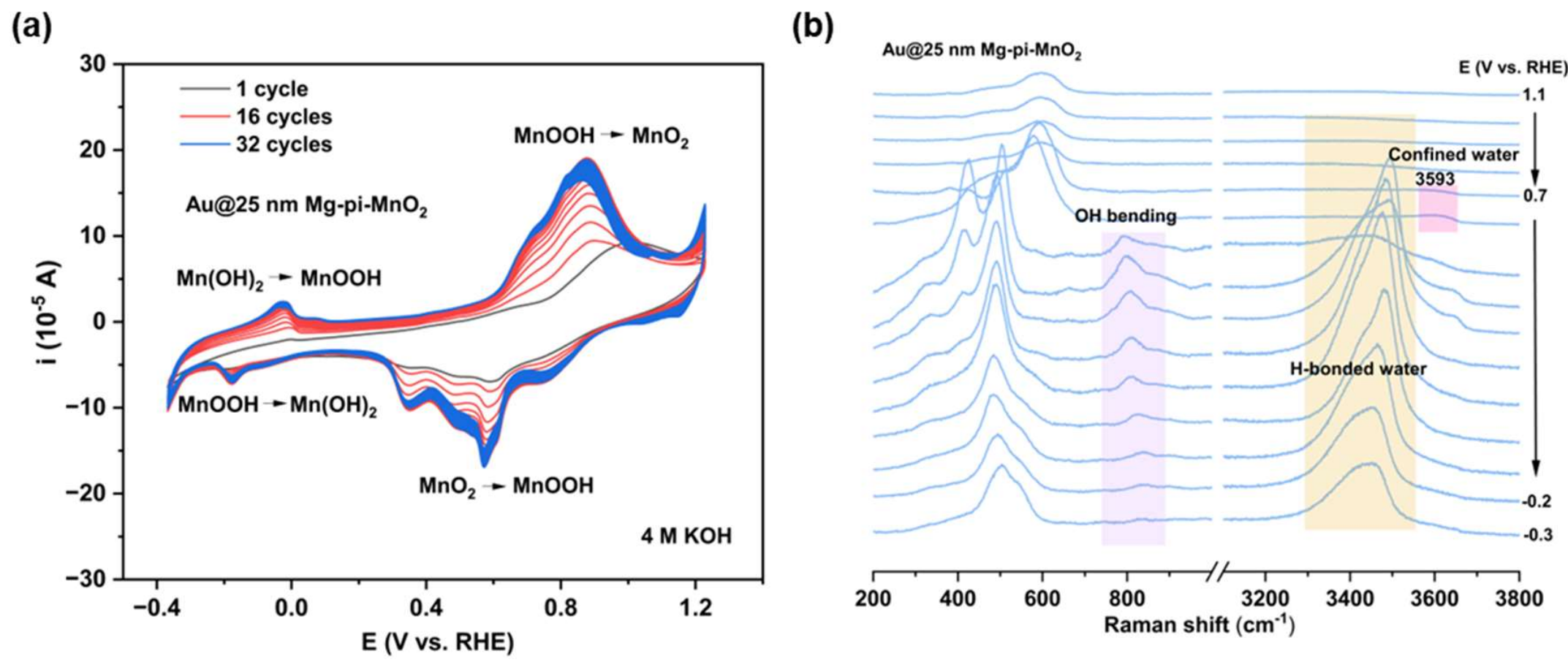

Supplementary Figure 17| a, Cyclic voltammetry (CV) curves of Au@25 nm Mg-pi-MnO_2 collected in 4 M KOH at a scan rate of 50 mV s^{-1} within a potential window of -0.40 to 1.20 V vs. RHE over different cycles. **b**, Corresponding in situ electrochemical SERS investigation of the discharge process on Au@25 nm Mg-pi-MnO_2 in 4 M KOH electrolyte.

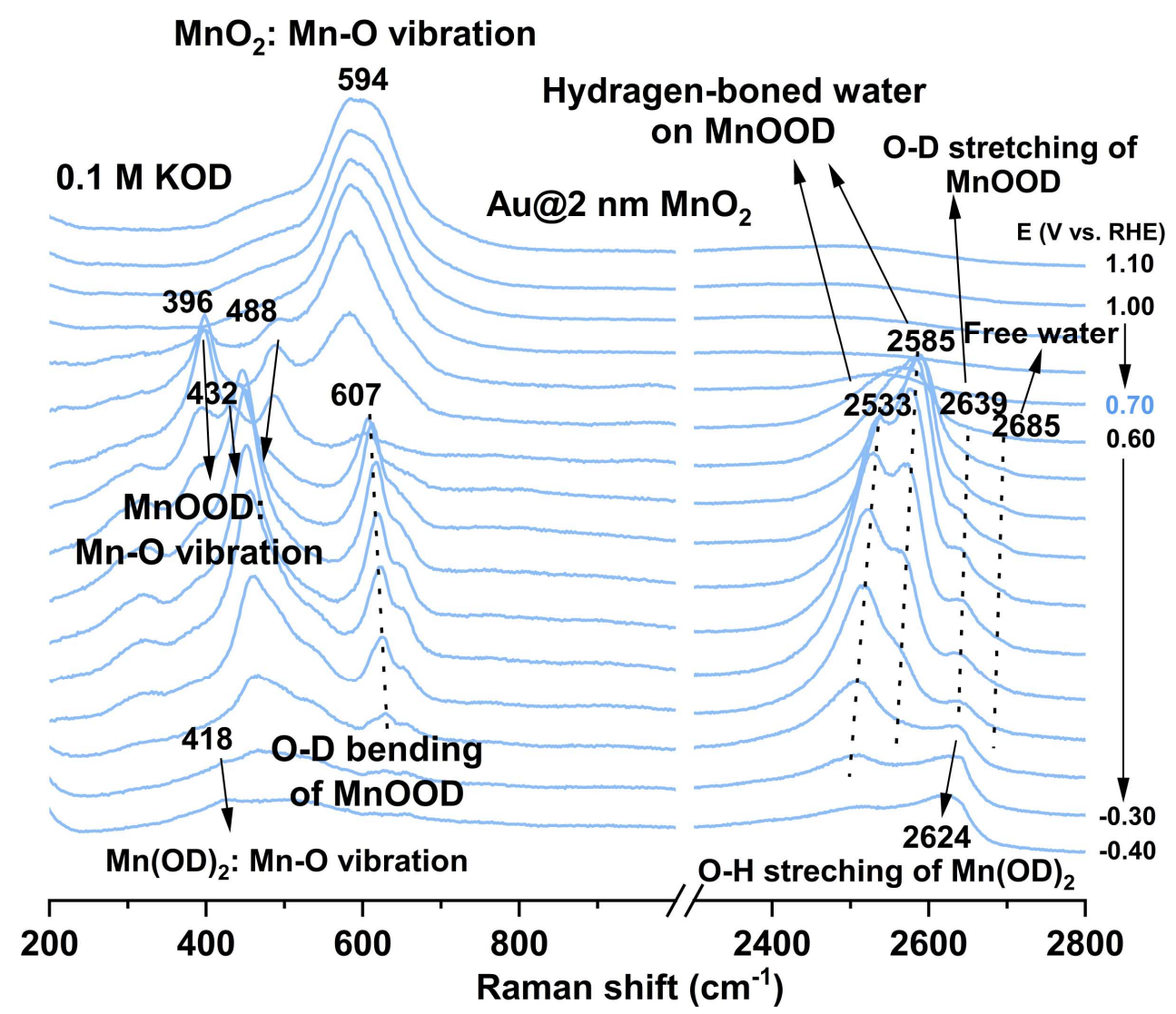

Supplementary Figure 18| In situ SERS investigation of the discharge process on Au@2 nm MnO_2 in 0.1 M KOD electrolyte.

At potentials near 0.7 V, water-related bands at ~3646, ~3485, and ~3412 cm^{-1} red-shift to ~2685, ~2585, and ~2533 cm^{-1} in D_2O isotopic experiment. The potential-dependent evolution of these features confirms the strong interaction of interfacial water with the MnO_2 surface and its participation in the PCET process: $MnO_2 + H_2O + e^- \rightarrow MnOOH + OH^-$. As the potential decreases below approximately 0.6 V vs. RHE, the characteristic Mn-O vibrational modes of MnO_2 progressively diminish, indicating a potential-driven transformation of MnO_2 into MnOOH. A prominent band appears at approximately 807 cm^{-1}, which red-shifts to ~607 cm^{-1} in D_2O, consistent with the O-H bending mode of an MnOOH-like intermediate. Additional bands at approximately 502, 457, and 410 cm^{-1}, assigned to Mn-O lattice vibrations, also exhibit modest downshifts to ~488, 432, and 396 cm^{-1}, respectively, upon isotopic substitution. These shifts arise from the adsorption of water molecules on the Mn-O framework of the MnOOH surface, forming a hydrogen-bonded configuration (H-O-H···O-Mn-O-H). Replacement of H_2O by D_2O alters the vibrational coupling within this hydrogen-bonded network (D-O-D···O-Mn-O-D), leading to the observed isotopic frequency shifts. Because all lattice oxygen atoms are bonded to hydrogen, substitution with deuterium leads to corresponding shifts in all Mn-O vibrational modes. The peak at ~3577 cm^{-1} is assigned to the O-H stretching vibration of MnOOH, which has shifted to 2639 cm^{-1} in D_2O isotopic experiment. As the potential decreases, this feature

intensifies, consistent with the further reduction of MnOOH to $Mn(OH)_2$ according to: $MnOOH + H_2O + e^- \rightarrow Mn(OH)_2 + OH^-$.

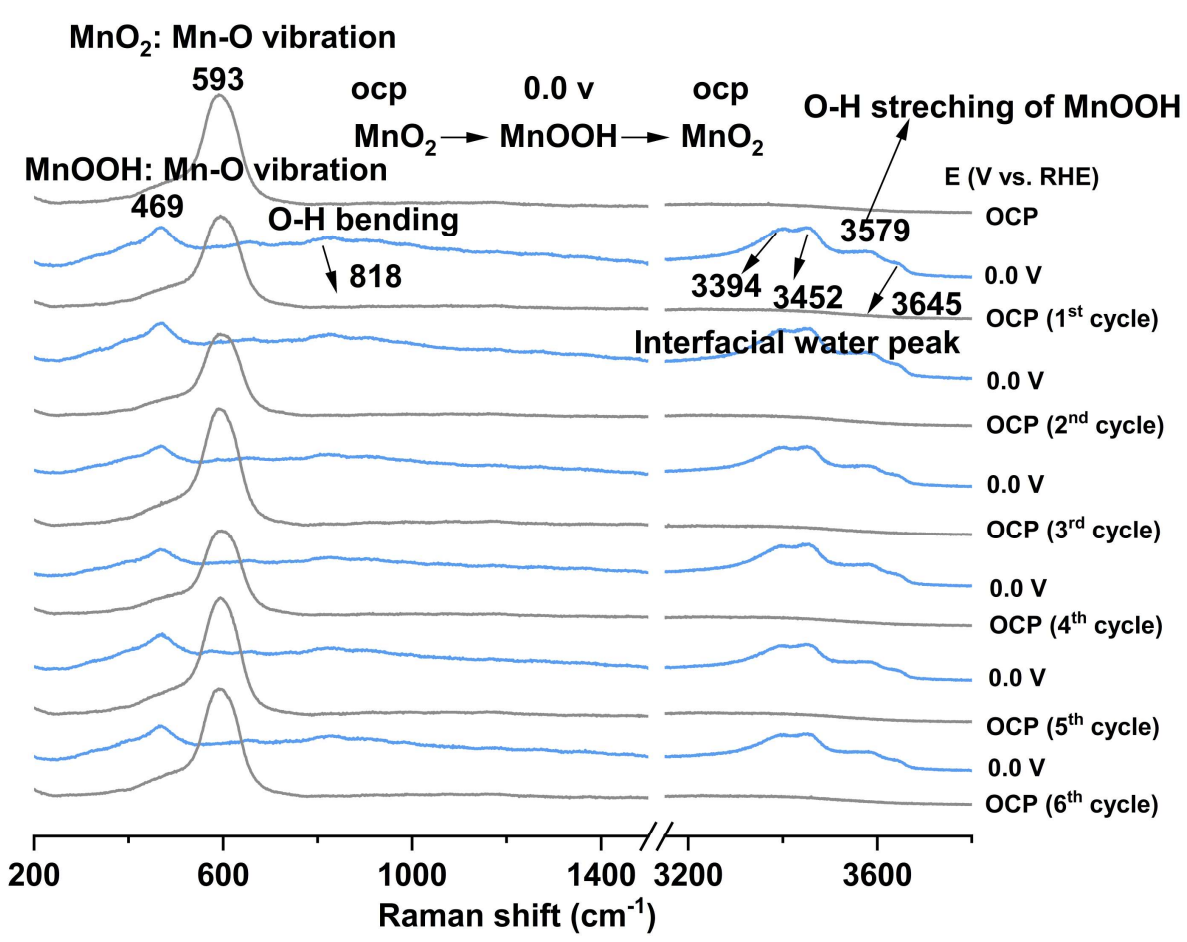

Supplementary Figure 19| Time-resolved operando Raman spectroscopy acquired by applying 0.0 V vs. RHE for 5 min, followed by instantaneous return to open-circuit potential (OCP) while continuously collecting spectra. Across all six repeated cycles, the characteristic MnO_2 Raman features reappear immediately upon removal of the applied potential, demonstrating that the MnOOH-like species forms exclusively under electrochemical bias. These observations indicate that the detected MnOOH is an in-situ- generated, short-lived intermediate, rather than a synthetically prepared or structurally stable MnOOH phase.

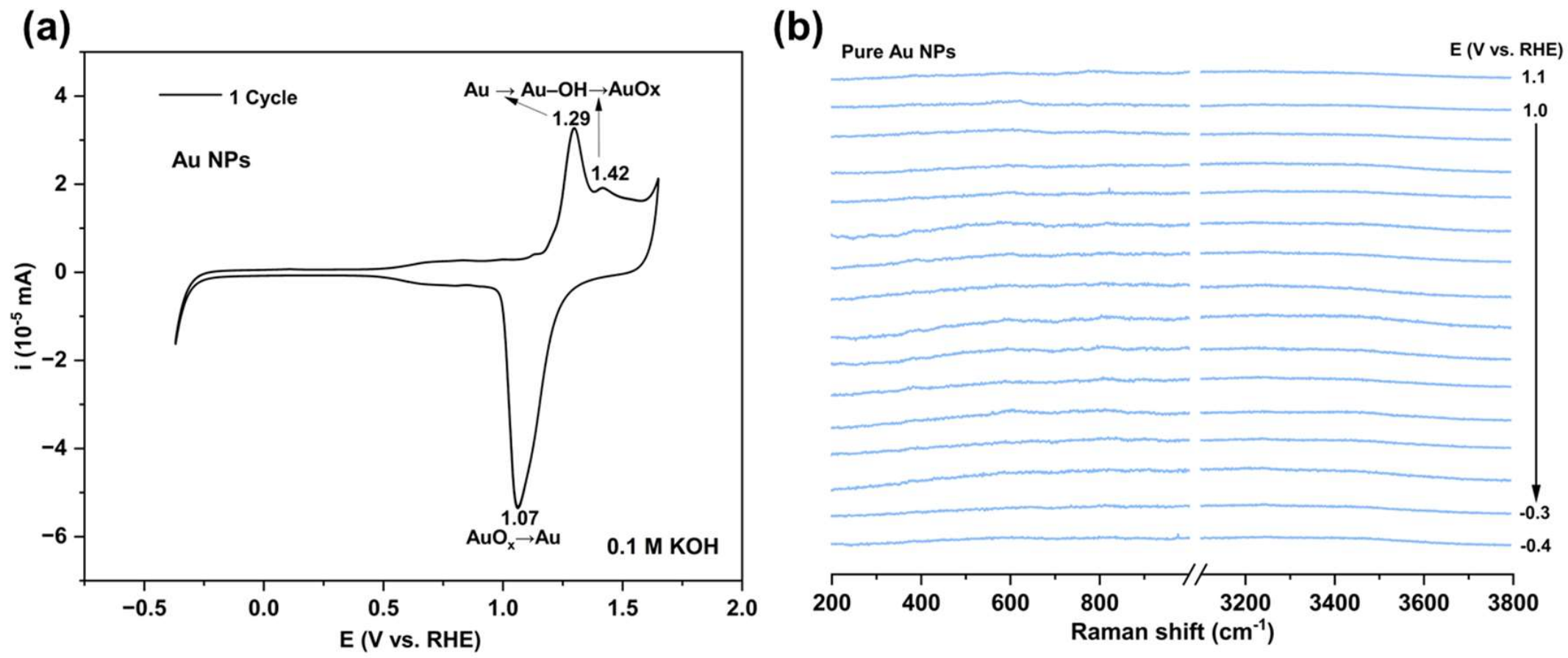

Supplementary Figure 20| a, CV curves of bare Au nanoparticles (Au NPs) recorded in 0.1 M KOH at a scan rate of 50 mV s^{-1}, within a potential window of -0.50 to 1.70 V vs RHE. **b**, Corresponding in situ Raman spectroscopy measurements of bare Au NPs collected in 0.1 M KOH over a potential range from 1.10 to -0.40 V vs. RHE vs RHE. As evidenced by the cyclic voltammetry, oxidation of Au occurs only at potentials above approximately 1.29 V vs RHE. Accordingly, within the potential range of 1.10 to -0.40 V vs. RHE, no discernible Raman features associated with Au-O species are observed. These results confirm that the electrochemical and spectroscopic signals discussed in the main text originate predominantly from the MnO_2 shell rather than the Au core.

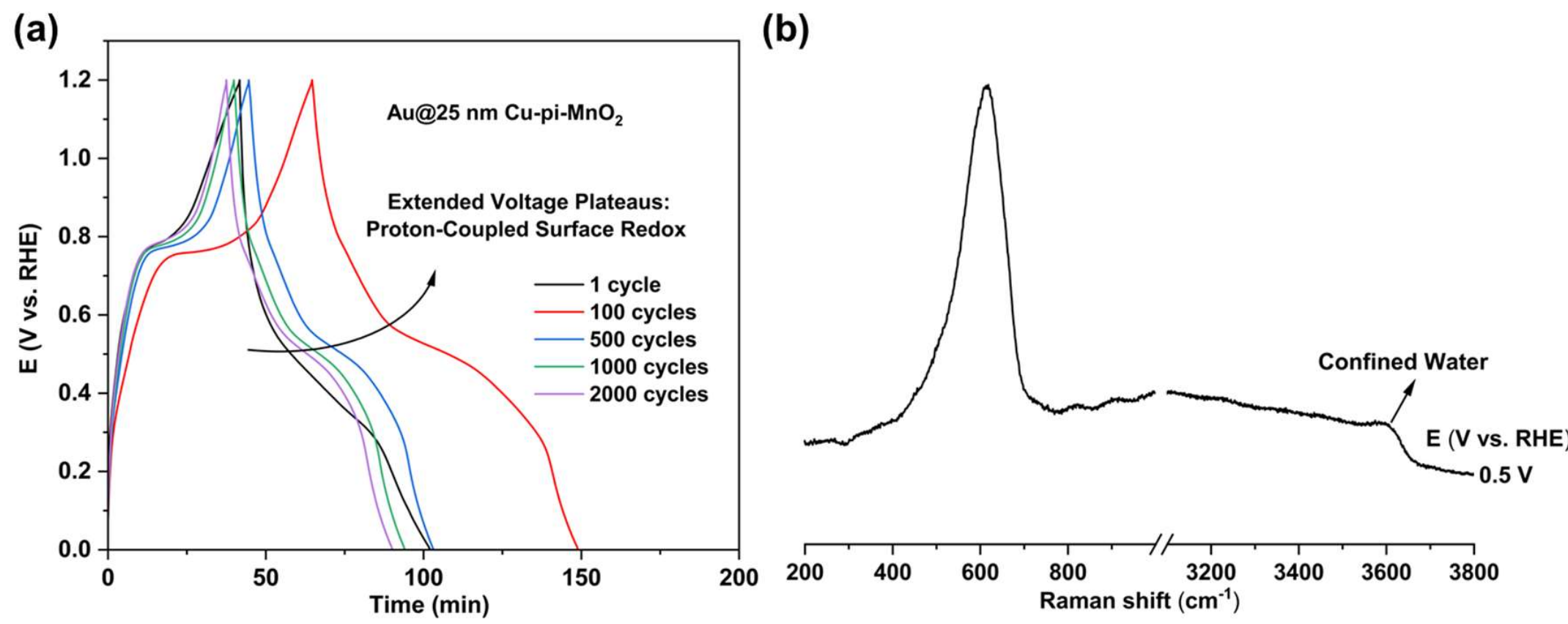

Supplementary Figure 21| a, GCD profiles of Au@25 nm Cu-pi-MnO_2 NPs measured within a potential window of 1.2 V vs. RHE at 20 A g^{-1}. **b**, Raman spectra collected at 0.5 V vs. RHE after the stability test.

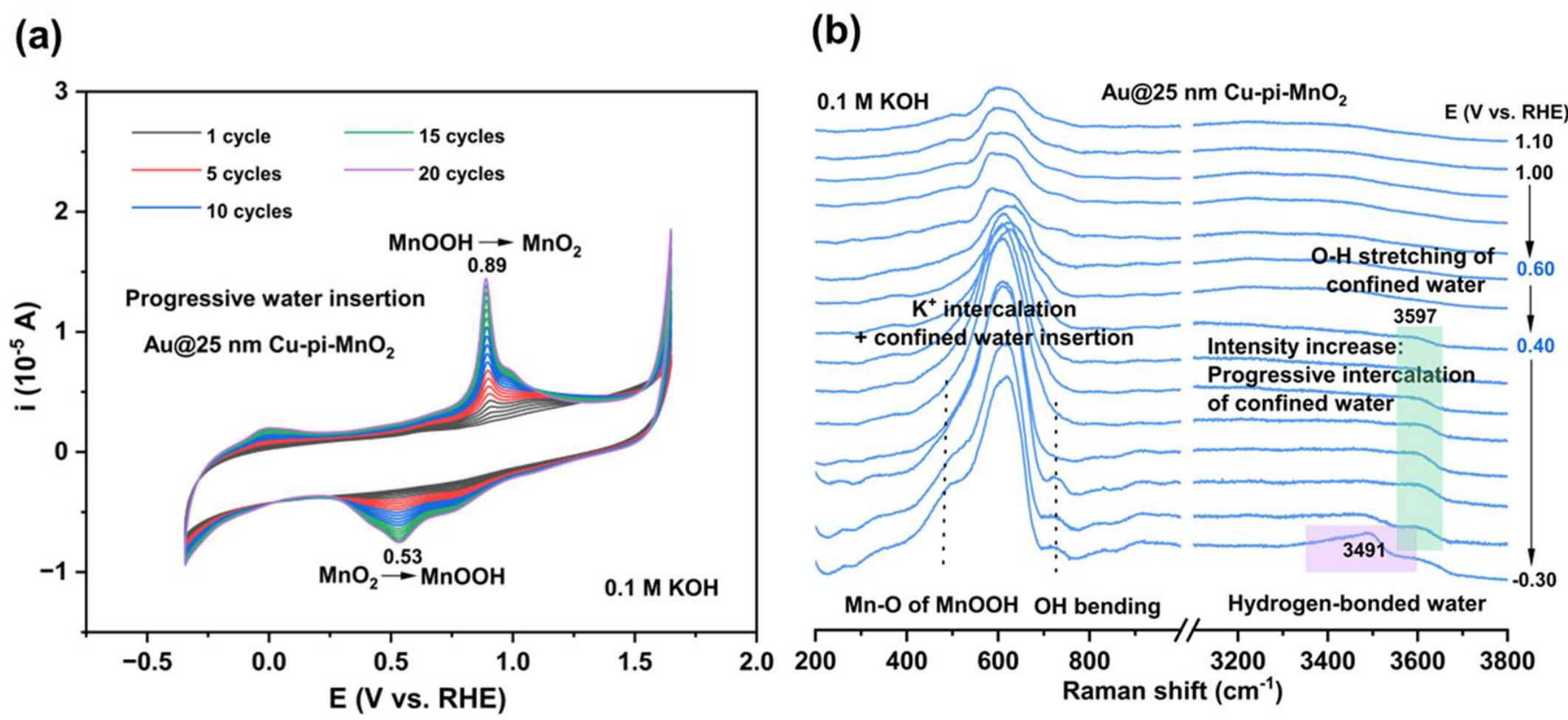

Supplementary Figure 22| Proton Intercalation Governed by Electrolyte Concentration-Controlled Nanoconfined Water Insertion Dynamics. a, CV analysis of Au@25 nm Cu-pi-MnO_2 nanoparticles in 0.1 M KOH at a scan rate of 50 mV s^{-1}. **b**, In situ electrochemical-SERS investigation of the discharge process on Au@25 nm Cu- pi-MnO_2 in 0.1 M KOH electrolyte.

Au@25 nm Cu-pi-MnO_2 exhibits a gradual emergence of distinct redox peaks, closely mirroring those of the Au@2 nm MnO_2 system in 0.1 M KOH. This time-dependent behavior reflects the slower intercalation of water into the interlayer space of Cu-pi-MnO_2, in contrast to the immediate water accessibility at the ultrathin interface. In situ Raman spectroscopy reveals increased Mn-O vibrational intensity at 0.6 V vs. RHE and the appearance of high-frequency O-H stretching bands at 0.4 V vs. RHE, indicating the insertion of cations and nanoconfined water. The progressive growth of these bands with continued polarization suggests ongoing intercalation of both cations and water. Concurrently, the emergence of a low-frequency MnOOH mode signals the activation of a proton-coupled redox process, enabled by the presence of intercalated water. At -0.3 V vs. RHE, a distinct Raman feature associated with hydrogen-bonded interlayer water becomes apparent, indicating the accumulation of nanoconfined water and the formation of hydrogen-bonded networks. Compared to the 4 M KOH system, where this feature appears much earlier, its delayed onset in 0.1 M KOH highlights significantly slower water insertion kinetics under dilute conditions.

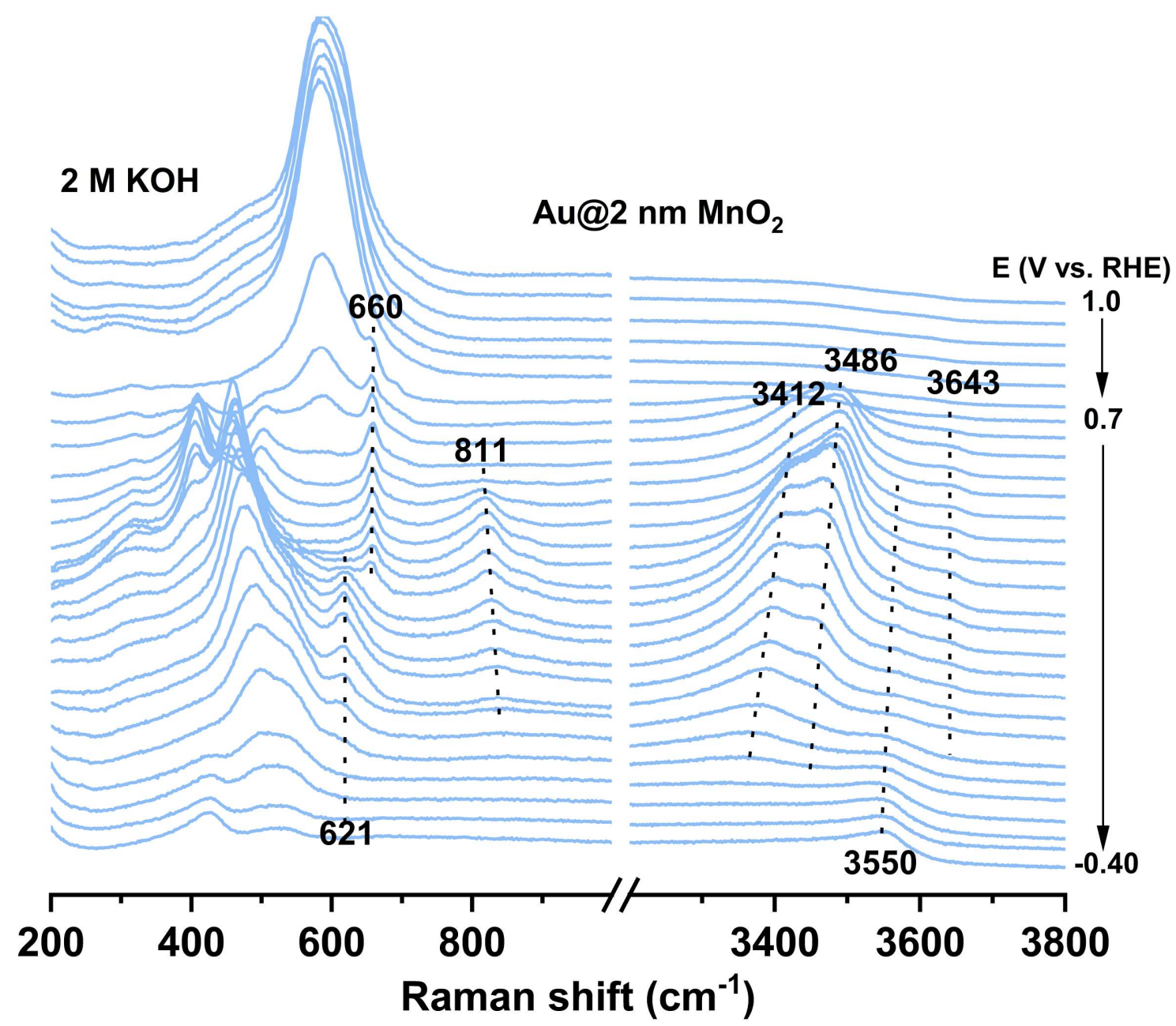

Supplementary Figure 23| In situ electrochemical SERS study of the discharge process on Au@2 nm MnO_2 in 2 M KOH solution.

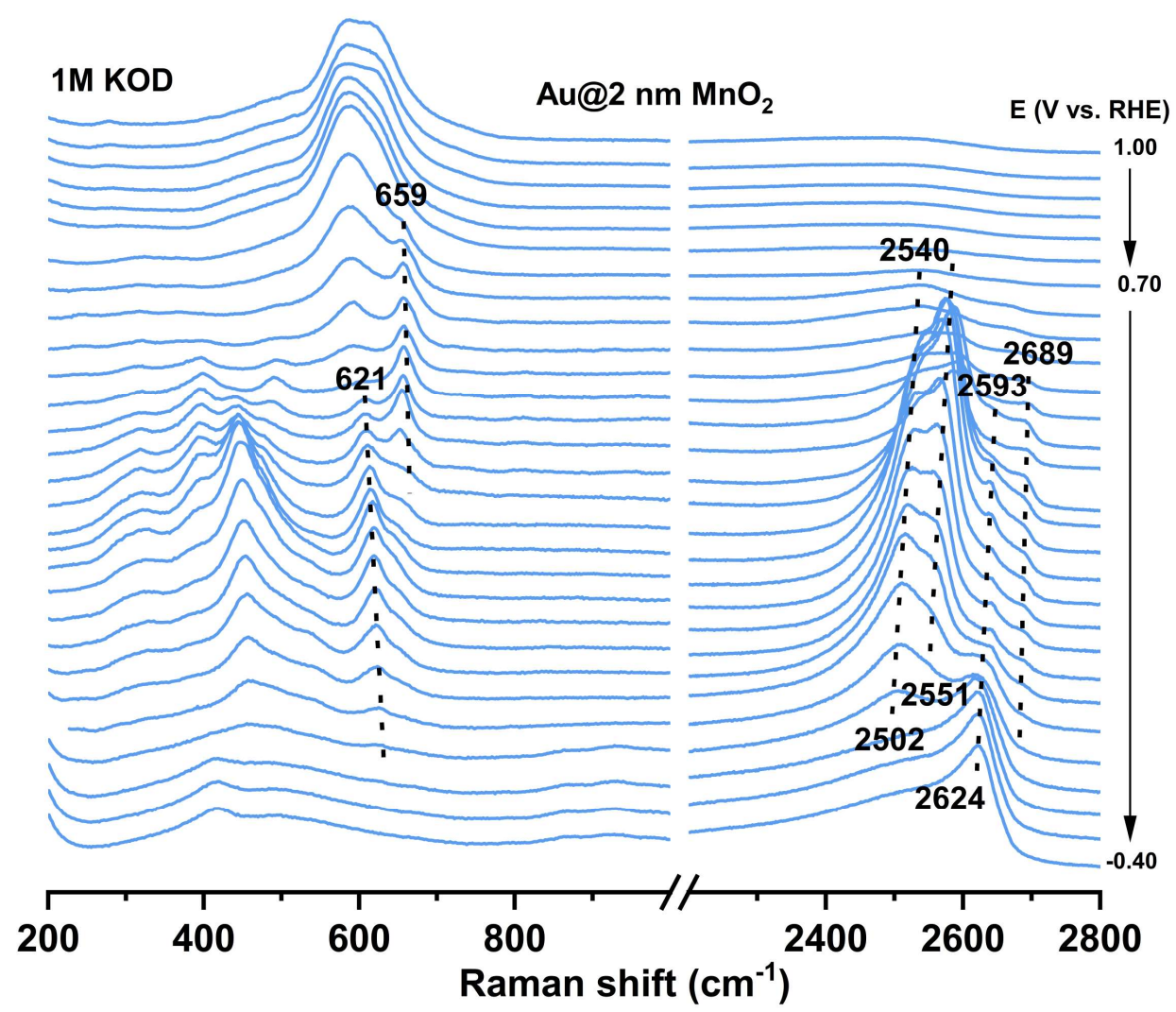

Supplementary Figure 24 | In situ electrochemical SERS study of the discharge process on Au@2 nm MnO_2 in 1 M KOD solution.

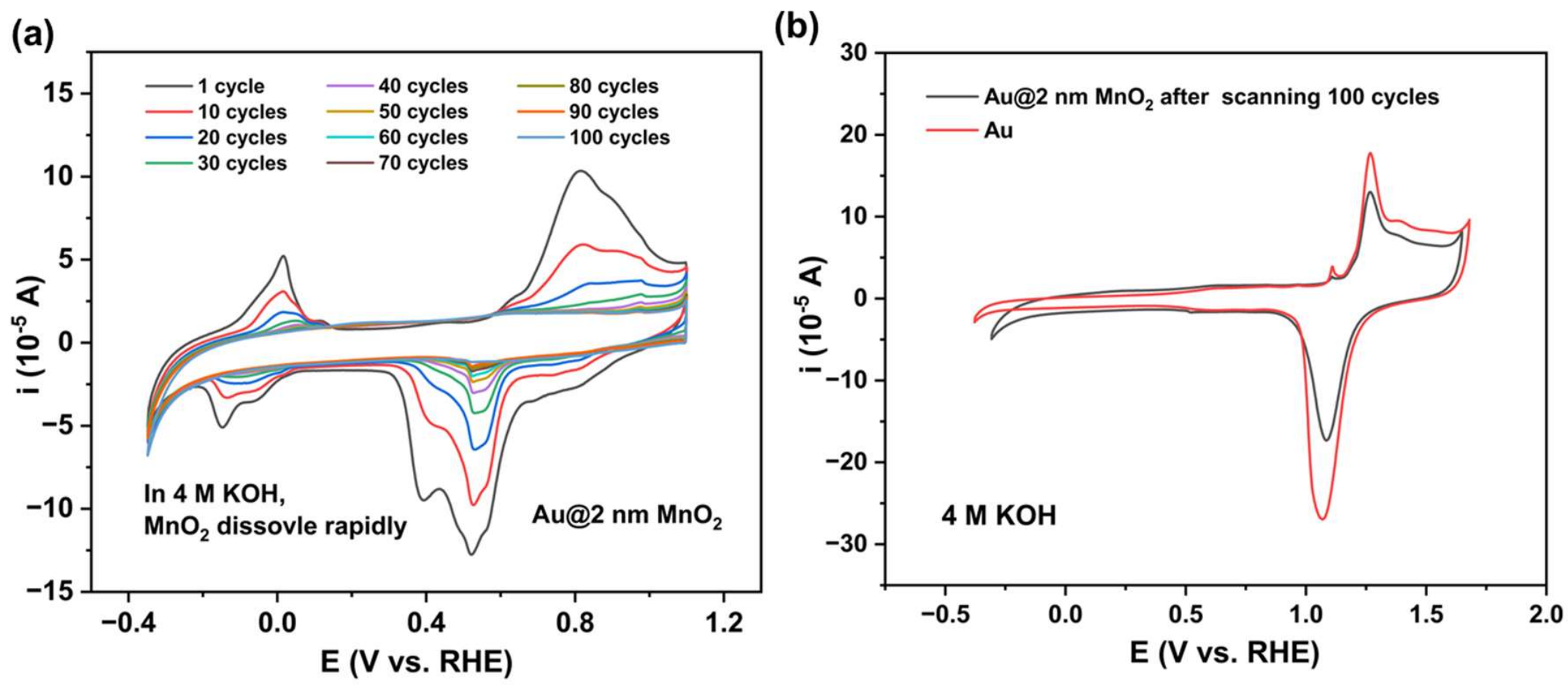

Supplementary Figure 25 | a, CV results of Au@2 nm MnO_2 with increasing scanning cycles at a scanning rate of 50 mV s^{-1} in 4 M KOH; **b**, CV testing of Au@2 nm MnO_2 NPs over 110 cycles in 4 M KOH electrolyte at 50 mV s^{-1}, and comparative CV profiles of pure Au NPs under identical conditions; After 110 cycles of CV testing in a 4 M KOH solution, the MnO_2 shell of Au@2 nm MnO_2 dissolves, resulting in CV profiles that are identical to those of pure Au NPs.

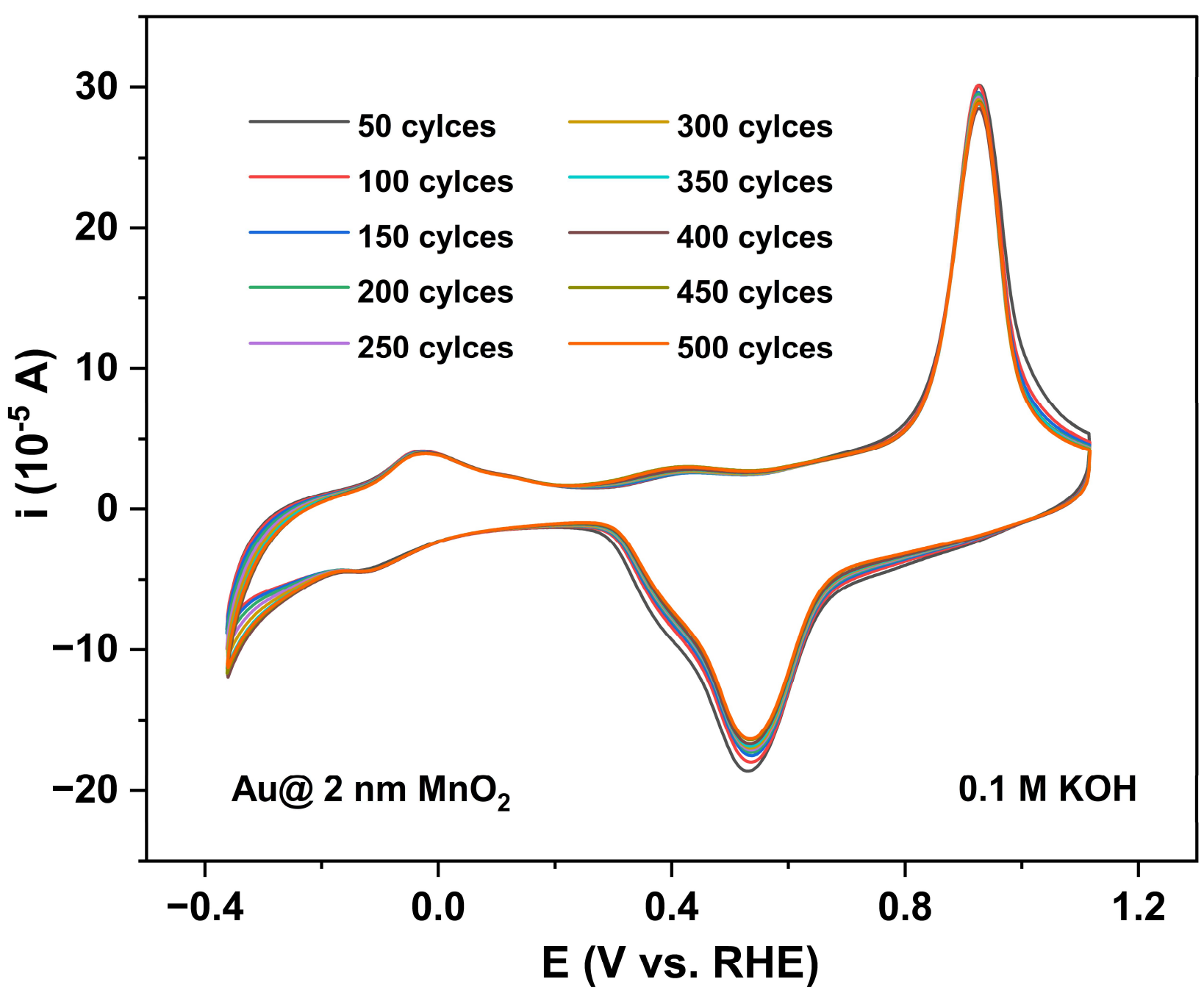

Supplementary Figure 26 | CV testing of Au@2 nm MnO_2 NPs in 0.1 M KOH solution at a scan rate of 50 mV s^{-1}.

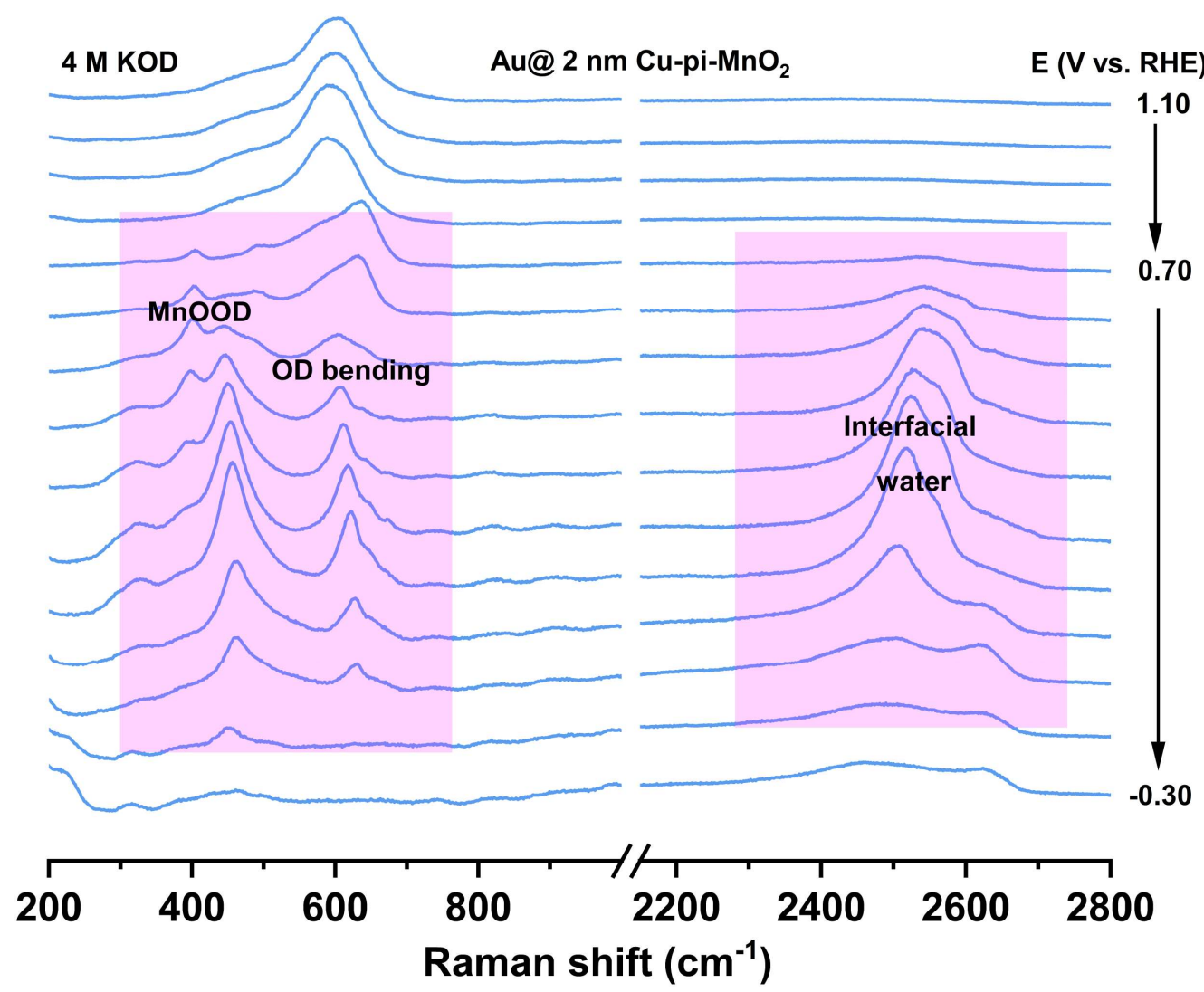

Supplementary Figure 27 | In situ electrochemical SERS study of the discharge process on Au@2 nm Cu-pi-MnO_2 in 4 M KOD solution.

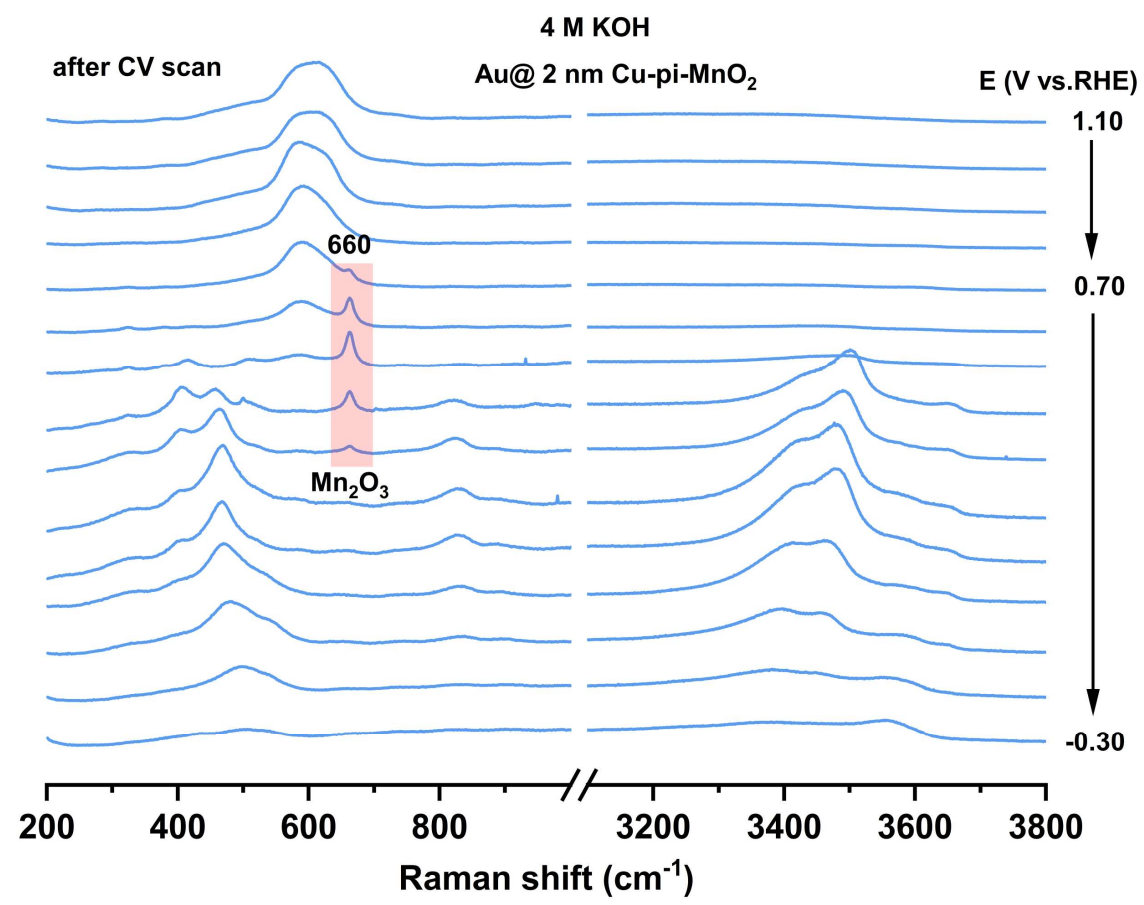

Supplementary Figure 28 | In situ electrochemical SERS study of the discharge process on Au@2 nm Cu-pi-MnO_2 in 4 M KOD solution after 100 CV Scanning.

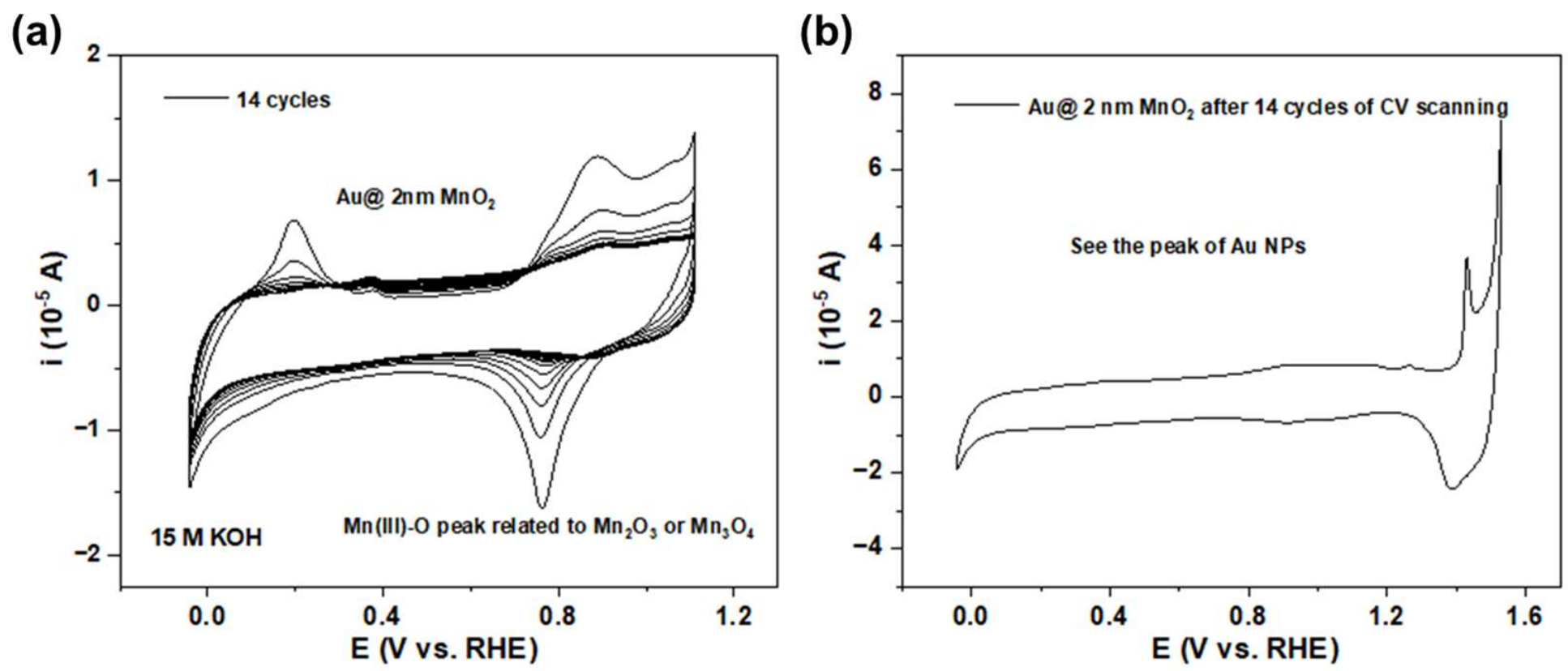

Supplementary Figure 29 | a, CV testing of Au@2 nm MnO_2 NPs in 15 M KOH electrolyte at 50 mV s^{-1} scan rate; **b,** Potential window expansion to around -0.04 to 1.53 V vs. RHE observed during 14 CV cycles in 15 M KOH, accompanied by dissolution of the MnO_2 shell in Au@2 nm MnO_2 NPs.

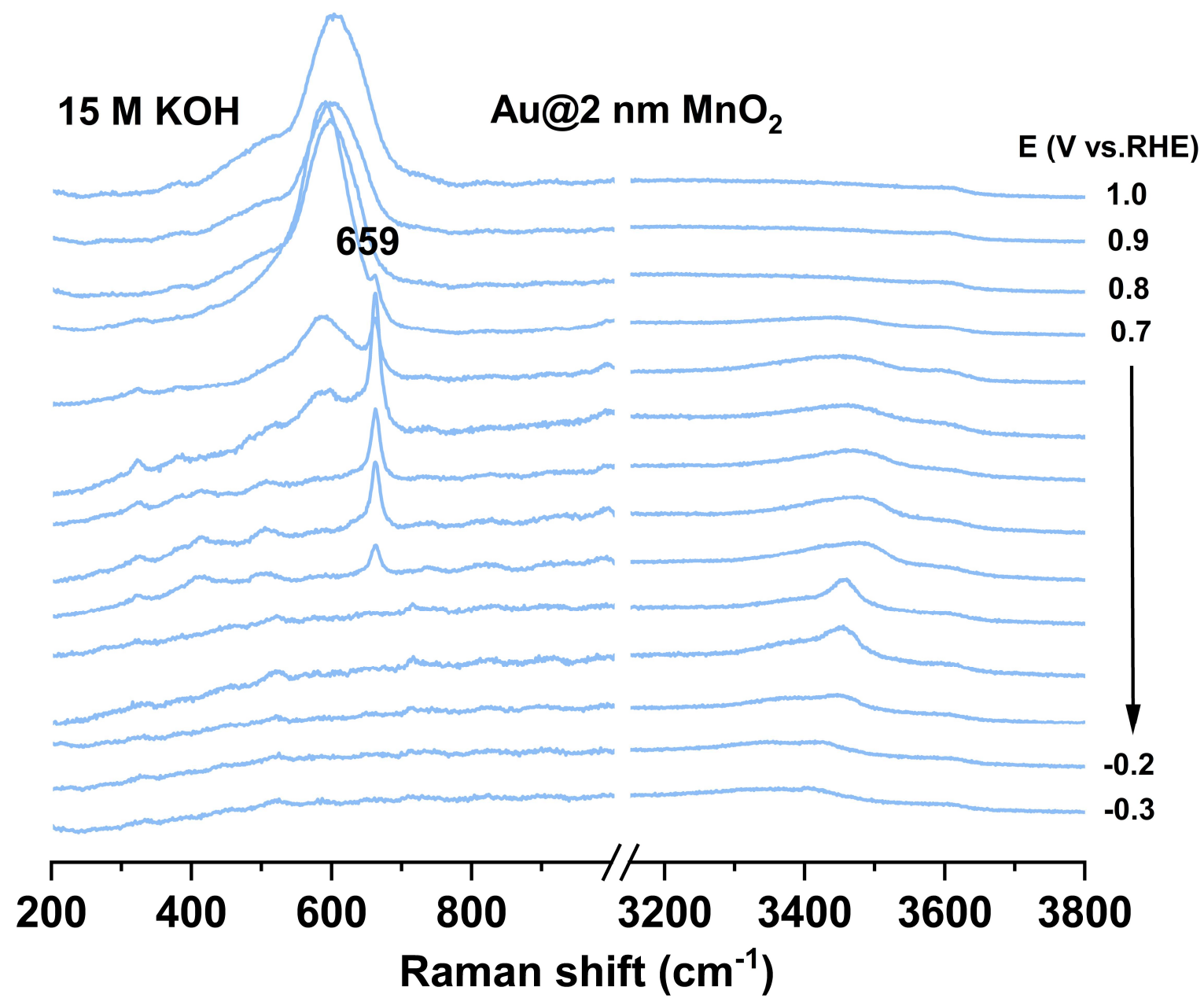

Supplementary Figure 30| In situ electrochemical SERS study of the discharge process on Au@2 nm MnO_2 surface in 15 M KOH solution.

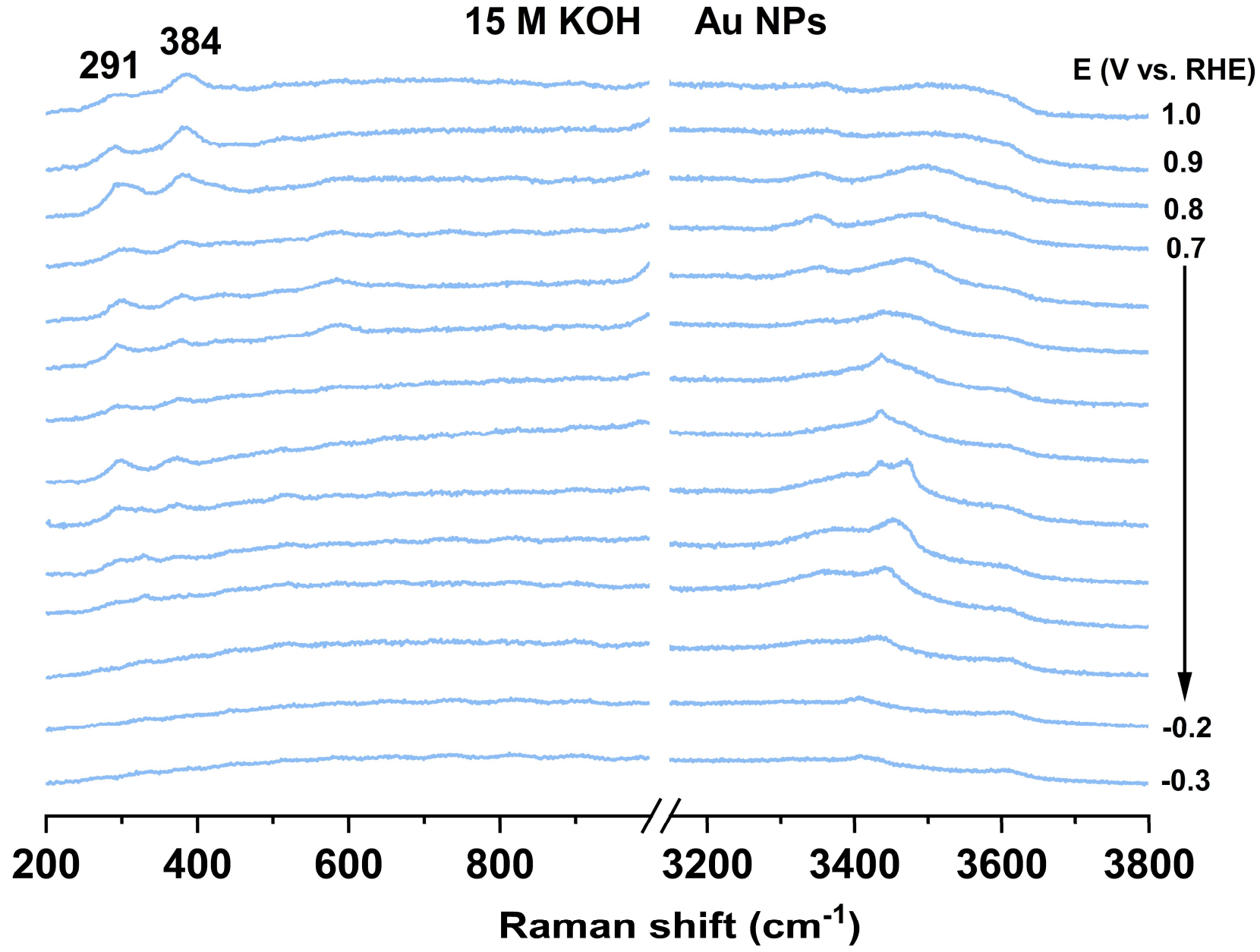

Supplementary Figure 31 | In situ electrochemical SERS study of the discharge process on pure Au surface in 15 M KOH solution.

References

1. Vasquez, R. P. CuO by XPS. *Surface Science Spectra*, **5**, 262-266 (1998)..
2. Lopez de Mishima, B. A. *et al*. XPS study of the MnO_2 electrode in borate solution during the discharge process. *Electrochim. Acta.* **36**, 1485–1489 (1991).
3. Stranick, M. A. MnO_2 by XPS. *Surf. Sci. Spectra* **6**, 31–38 (1999).
4. Julien, C. M. *et al*. Electrochemical oxidation of Mn/MnO films: formation of an electrochemical capacitor. *Spectrochim. Acta.* **60**, 689 (2004).
5. Chen, D. *et al*. Probing the charge storage mechanism of a pseudocapacitive MnO_2 electrode using in operando Raman spectroscopy. *Chem. Mater.* **27**, 6608–6619 (2015).
6. Yang, L. *et al*. Investigation into the origin of high stability of δ-MnO_2 pseudo-capacitive electrode using operando Raman spectroscopy. *Nano Energy* **30**, 293–302 (2016).
7. Ze, H. *et al*. Deciphering the competitive charge storage chemistry of metal cations and protons in aqueous MnO_2-based supercapacitors. *J. Am. Chem. Soc.* **147**, 9620-9628 (2025).
