# Supplementary material for: Metal pre-intercalation promotes water-mediated proton-coupled electron transfer in layered δ-MnO2 for aqueous pseudocapacitive energy storage
Source: Nat Commun. 2026 Jul 28;17:9168. doi: 10.1038/s41467-026-76022-4 (PMC13522339; doi:10.1038/s41467-026-76022-4)
Supplement: Supplementary file 2 [file 41467_2026_76022_MOESM2_ESM.pdf]

Description of Additional Supplementary Files

File Name: Supplementary Data 1
Description: Supplementary Data 1 contains optimized structural models and DFT-calculated energy data for the interlayer migration of K^+ in Cu-pi-MnO_2.

File Name: Supplementary Data 2
Description: Supplementary Data 2 contains optimized structural models and DFT-calculated energy data for the interlayer migration of H^+ in Cu-pi-MnO_2.

File Name: Supplementary Data 3
Description: Supplementary Data 3 contains optimized structural models and DFT-calculated energy data for the interlayer migration of H_2O in Cu-pi-MnO_2.

File Name: Supplementary Data 4
Description: Supplementary Data 4 contains optimized structural models and DFT-calculated energy data for the water-assisted interlayer migration of H^+ in Cu-pi-MnO_2.
