# Supplementary material for: Metal pre-intercalation promotes water-mediated proton-coupled electron transfer in layered δ-MnO2 for aqueous pseudocapacitive energy storage
Source: Nat Commun. 2026 Jul 28;17:9168. doi: 10.1038/s41467-026-76022-4 (PMC13522339; doi:10.1038/s41467-026-76022-4)
Supplement: Supplementary file 3 [file 41467_2026_76022_MOESM7_ESM.pdf]

nature portfolio

Peer Review File

Metal Pre-Intercalation Promotes Water-Mediated Proton-Coupled Electron Transfer in Layered δ-MnO2 for Supercapacitor Energy Storage

Corresponding Author: Dr Edward Sargent

This file contains all reviewer reports in order by version, followed by all author rebuttals in order by version.

Version 0:

Reviewer comments:

Reviewer #1

(Remarks to the Author)
In this manuscript, the authors systematically investigated the role of Cu2+ pre-intercalation in modulating the charge storage mechanism of δ-MnO2 and proposed a transition from cation-dominated to proton-dominated pathways supported by various in situ spectroscopic techniques and theoretical calculations. It represents an interesting study with a rigorous experimental design and substantial supporting data, however, several aspects require further clarification. Here are some points the authors should address.
1. The manuscript suggests that the pre-intercalation of Co2+ and Mg2+ can also induce a proton-dominated charge storage mechanism. Could the authors elaborate on the structural similarities between Co2+/Mg2+ and Cu2+ pre-intercalation in facilitating this mechanism?
2. While the Au core is rational for SERS enhancement, its potential influence on the charge storage behavior of MnO2 (e.g., on electronic conduction or the interfacial electric field) requires assessment.
3. The confined water and its hydrogen-bonded network are key to the proposed mechanism. Could the authors provide evidence that this network is stable after prolonged electrochemical cycling ?
4. Although it is suggested that Ni2+ substitutes Mn sites instead of occupying interlayer sites, this conclusion would be strengthened by direct structural characterization (e.g., XANES) of the Ni2+ local coordination environment.
5. Please check the format problem further. For example, the label for Figure 3d is given as "Au@2 nm MnO2 NPs", which seems to be a duplicate of Figure 3c. Please check.
6. Regarding methods, it's not clear what has been utilized from the existing work and what is new. It should be clearly discussed. Some important refs may be cited to enrich reference part of the revised manuscript, such as, Electrochem. Energy Rev. 2023, 6(1), 5; Electrochem. Energy Rev. 2023, 6, 33.

Reviewer #2

(Remarks to the Author)
The charge storage capacitance of delta-MnO_2 pseudocapacitors originates from two mechanism, the intercalation of cations and protons as well as their adsorption on the surface. The introduction of Cu ions suppresses structural changes of the layered phase and enhances the capacitor performance. In this study, the authors investigated the role of Cu ions by combining electrochemical measurements with in situ surface-enhanced Raman spectroscopy under various conditions such as pH, film thickness, and electrolyte cation species. They demonstrated a correlation between surface structural changes and film performance, namely the emergence of a proton-coupled redox mechanism mediated by interlayer water molecules and the observed enhancement in capacitor performance.
The synthesis of the samples and their physicochemical characterization were adequately carried out and explained in a logical manner (Fig. 1). The enhancement of capacitance upon Cu pre-intercalation was also experimentally demonstrated (Figs. 2e and 2f). However, there remain concerns regarding the novelty of the spectroscopic methodology, the interpretation of the interfacial processes, the analysis of the data, and the consistency of the explanations. For these reasons, I do not consider the work to meet the standards required for publication in Nature Communications. I therefore cannot recommend the manuscript for further consideration for publication in this journal.

Major Points
Use of Raman Enhancement Substrates
Gold nanoparticles coated with Mn oxide layers of different thicknesses were synthesized and employed as Raman enhancement elements. However, this approach may lead to the observation of surface information based on fundamentally different principles depending on the coating thickness. The SHINERS method (Li et al., Nature, 2010 and subsequent works) has already clarified the relation between oxide shell thickness and Raman enhancement, showing that the signal decays exponentially with increasing thickness. The enhancement is effective only within a few nanometers and is severely attenuated beyond ~10 nm. In this study, Mn oxide coatings of 2 nm and 25 nm were prepared and used. At 2 nm, selective enhancement of interfacial Raman signals can be expected, whereas at 25 nm, little enhancement should remain, and the measurement essentially corresponds to normal Raman spectroscopy. Indeed, in the thin-shell case, detailed potential dependence was observed even in the low-frequency region, while in the thick-shell case, the spectra were weaker, broader, and lacked clear potential dependence. Thus, the former likely provides selective surface information, whereas the latter reflects bulk-averaged information. Direct comparison of the two cases may lead to misinterpretation, and this requires careful consideration.
Nevertheless, the 2 nm case seems appropriate for the authors' intended purpose of probing surface behavior in situ condition. If one intends to obtain molecular information from the surface of a 25-nm Mn Oxide film using Raman spectroscopy for comparison purpose with that of 2-nm shell, this can be achieved by employing the SHINERS method, which is applicable even under strong alkaline and electrochemical conditions. In practice, this would involve placing shell-isolated nano-amplifiers, resistant to concentrated alkali, on the Mn oxide surface. Recently, it was reported that the zirconia shell-isolated nano-amplifiers enable electrochemical in situ Raman measurement in 1 M KOH solution.

Assignment of Raman Bands
The manuscript assigns Raman bands at 727, 504, 461, and 413 cm^{-1} (or 808, 503, 457, 410 cm^{-1}) to MnOOH, but no supporting references or computational evidence are provided. Previous reports consistently assign MnOOH signals around 625, 560, and 530 cm^{-1}, which differ markedly from the present results. While the emergence of new Raman bands concomitant with redox processes in CV is understandable, this does not constitute proof of MnOOH formation. The basis for the current assignment must be clarified.
Furthermore, the manuscript states that these Raman bands shift to 492, 447, and 401 cm^{-1} upon using D_2O, attributed to an isotope effect. However, if these bands corresponded to OH-related vibrations, both stretching and bending modes should shift to ~0.71-fold of the original wavenumber. In contrast, the observed shifts correspond to ~0.97-fold, suggesting instead that these are modes such as O–Mn–O bending or lattice vibrations in which hydrogen (or deuterium) contributes minimally. The authors should discuss this possibility explicitly and provide a consistent explanation for the assignments, isotope shifts, and surface structures inferred from the spectra.

Experimental Parameters and Comparisons
The study varies several parameters simultaneously (electrolyte concentration, MnO_2 film thickness, presence/absence of Cu, cation species), but comparisons based on multiple changing parameters do not allow for clear conclusions. Only one parameter should be varied at a time. While a broad range of observations is presented, the discussion becomes complex and does not arrive at a comprehensive understanding.

Minor Points
The synthesis of gold nanoparticles is described using the traditional Frens method with Na citrate reported in Nature 1973. However, the original procedure uses a gold salt concentration of 0.01%; this should be checked for consistency.

The reported maximum capacitance values should be compared against benchmark values from previous studies on similar materials; otherwise, their significance remains unclear.

The authors suggest that Cu ions substitute for K ions. However, the EDX mapping shown in Fig. 1g and Supplementary Fig. 4 does not support Cu distribution in the spine-like structures visible at the lower center of the image. Is there direct evidence that Cu ions are incorporated into the crystal interior rather than merely adsorbed at the surface other than XRD data?

In Fig. 1b, it would be informative to include capacitance data for MnO_2 without Cu ions in 4 M KOH electrolyte. This would allow for a direct comparison of the effects of electrolyte concentration and Cu incorporation.

Overall Assessment

While the manuscript demonstrates careful experimental work and highlights an important problem, the spectroscopic assignments and data interpretation lack sufficient rigor and novelty for Nature Communications. Significant revisions and more conclusive evidence would be required before further consideration.

Reviewer #3

(Remarks to the Author)
This work investigates the mechanistic origins of the enhanced capacitance in δ-MnO2 with pre-intercalated Cu2+. Transition metal pre-intercalation promotes the retention of interlayer-confined water, and this supports proton-coupled charge storage. Similar mechanistic transition is also evident in δ-MnO2 systems pre-intercalated with other transition metal ions, such as Co2+ and Mg2+. This work offers a route to design high-performance MnO2 electrodes. I recommend its

publication after major revision

1. In this work, Au@δ-MnO_2 and Au@Cu-pi-δ-MnO_2 core-shell nanostructures were prepared for SERS. The introduction of Au core will change the electrical conductivity and transmission path of cation to proton. They would influence charge storage. Related experiments and discussion could be included in this work.
2. In some reference, the MnO_2 without pre-intercalated metal ions also display proton-coupled charge storage. Authors could further clarify the conclusion of this work.
3. In this work, SERS was used to investigate proton-coupled charge storage. More characterizations could be carried out to understand the proton-coupled charge storage.
4. The size of δ-MnO_2 would also influence charge storage. Related experiments and discussion could be included in this work.
5. To illustrate the feasibility of pre-intercalated Cu^{2+}, large-capacity full batteries could be assembled and measured.

Reviewer #4

(Remarks to the Author)
The authors investigate the regulation of the charge-storage mechanism in δ-MnO_2 through metal-ion pre-intercalation. The study presents a conceptually interesting idea and attempts to correlate structural, electrochemical, and spectroscopic observations to the behavior of interlayer water and proton transport. However, the current experimental evidence is insufficient to support the proposed mechanism, and critical structural information remains unclear. There are still several fundamental issues and mechanistic details that need to be resolved; therefore, the manuscript is not yet suitable for publication in Nature Communications.

1. Regarding the "water-retention effect," the authors mainly rely on Raman peak shifts without providing quantitative or direct evidence to verify the presence and variation of interlayer water.

2. The discussion of Ni-ion doping is mainly based on references concerning α-MnO_2, whereas the material studied here is δ-MnO_2 with a different framework structure. The authors should clarify how such structural differences affect the behavior of Cu^{2+}, and further distinguish between the mechanisms of lattice doping and interlayer intercalation, as well as explain why Cu^{2+} tends to remain interlayered rather than incorporated into the lattice.

3. The high specific capacitance value obtained after "subtracting the Au contribution" may be overestimated, as interfacial coupling effects are generally not linearly separable. Such a correction method could misrepresent the intrinsic performance of the active material.

4. The current characterization results only confirm the presence of Cu in the samples but are insufficient to demonstrate that Cu ions are indeed located within the interlayer region and remain stable during cycling. It is recommended to examine the electrochemical performance of Cu-pi-MnO_2 electrodes without the Au core to verify whether the proposed mechanism holds in a practical configuration.

5. Since Cu^{2+} carries a higher charge than K^+, charge compensation through valence adjustment or anion incorporation should occur; however, this aspect has not been addressed or discussed in the manuscript.

6. The manuscript presents electrochemical data for Co^{2+}- and Mg^{2+}-intercalated samples. Given the significant differences in ionic radius, charge density, and coordination characteristics among these cations, additional structural and spectroscopic analyses are required to determine whether these ions act via similar mechanisms.

7. The Au@MnO_2 core–shell structure (~1 µg active material) differs markedly from practical electrodes. The Au core may alter the electrochemical response and Raman signals via enhanced conductivity and local field effects, so its influence should be carefully considered when interpreting the intrinsic behavior of MnO_2.

Version 1:

Reviewer comments:

Reviewer #1

(Remarks to the Author)
The authors carefully addressed all comments. This manuscript is acceptable for publication.

Reviewer #2

(Remarks to the Author)
The authors have clearly devoted substantial effort and time to addressing the reviewers' comments and have performed additional experiments to support their arguments. Their responses are generally logical and provide reasonable clarification of the points raised during the initial review process. The revised manuscript is also noticeably improved in terms of readability and clarity compared with the original submission.

Although the study provides useful spectroscopic insights, the overall mechanistic concept—proton-coupled charge storage in MnO_2 systems—has been discussed in prior literature. Therefore, the conceptual advance of the present work appears to lie mainly in the spectroscopic investigation rather than in the mechanistic framework itself. From the perspective of conceptual novelty, the advance presented here may therefore be somewhat limited, particularly in the context of the level of conceptual innovation typically expected for a journal such as Nature Communications.

Overall, while the study provides useful experimental observations, the level of conceptual advance may not fully meet the threshold typically expected for publication in a high-impact journal such as Nature Communications. In its current form, the manuscript may be better suited for a more specialized journal.

Reviewer #4

(Remarks to the Author)
The author's revisions and modifications are comprehensive, thus it is recommended for publication in Nature Communications.

Open Access This Peer Review File is licensed under a Creative Commons Attribution 4.0 International License, which permits use, sharing, adaptation, distribution and reproduction in any medium or format, as long as you give appropriate credit to the original author(s) and the source, provide a link to the Creative Commons license, and indicate if changes were made.
In cases where reviewers are anonymous, credit should be given to 'Anonymous Referee' and the source.
The images or other third party material in this Peer Review File are included in the article's Creative Commons license, unless indicated otherwise in a credit line to the material. If material is not included in the article's Creative Commons license and your intended use is not permitted by statutory regulation or exceeds the permitted use, you will need to obtain permission directly from the copyright holder.
To view a copy of this license, visit https://creativecommons.org/licenses/by/4.0/

We sincerely thank the Editor and all Reviewers for their careful evaluation of our manuscript and for the constructive, detailed, and insightful comments. Below, we provide a point-by-point response to each reviewer's comments.

Reviewer #1 (Remarks to the Author):

In this manuscript, the authors systematically investigated the role of Cu^{2+} pre-intercalation in modulating the charge storage mechanism of δ-MnO_2 and proposed a transition from cation-dominated to proton-dominated pathways supported by various in situ spectroscopic techniques and theoretical calculations. It represents an interesting study with a rigorous experimental design and substantial supporting data; however, several aspects require further clarification. Here are some points the authors should address.

Reviewer #1, comment 1. The manuscript suggests that the pre-intercalation of Co^{2+} and Mg^{2+} can also induce a proton-dominated charge storage mechanism. Could the authors elaborate on the structural similarities between Co^{2+}/Mg^{2+} and Cu^{2+} pre-intercalation in facilitating this mechanism?

Response:

We have performed additional *in-situ* electrochemical SERS measurements on Co^{2+}- and Mg^{2+}-pre-intercalated MnO_2. Consistent with the electrochemical CV results, these in situ spectroscopic data show that the effects of Co^{2+} and Mg^{2+} pre-intercalation are analogous to Cu^{2+}. The corresponding spectroscopic data have been added to the Supplementary Information (Supplementary Figs. 16-17). On the main text page 7, we now write:

The spectroscopic and electrochemical results suggest that pre-intercalation of Co^{2+} and Mg^{2+}, which replace native K^+ ions, facilitates the insertion of interlayer-confined water and similarly promotes proton-coupled intercalation (Supplementary Figs. 16-17).

We now provide XRD measurements showing successful pre-intercalation of Co^{2+} and Mg^{2+} into the interlayer spacing of MnO_2 (Supplementary Figs. 15), updating on page 6:

"XRD measurements of Co-pi-δ-MnO_2 and Mg-pi-δ-MnO_2 similarly reveal a contraction in interlayer spacing, confirming successful pre-intercalation (Supplementary Fig. 15)."

Reviewer #1, comment 2. While the Au core is rational for SERS enhancement, its potential influence on the charge storage behavior of MnO_2 (e.g., on electronic conduction or the interfacial electric field) requires assessment.

Response:

Our control experiments demonstrate that the Au core is electrochemically inert within the operating potential window and does not contribute to the observed charge storage or spectroscopic signatures. We performed control CV and *in-situ* SERS measurements on bare Au nanoparticles (Supplementary Fig. 20), finding Au oxidation potentials > 1.29 V vs RHE. We do not observe Raman features associated with Au-O species in the potential range of 1.10 to -0.40 V vs RHE. These exclude the contributions of Au core to the electrochemical and spectroscopic signals. These are summarized in Supplementary Fig. 20. In page 7, we write:

"As an additional control, we also present the CV and in-situ SERS results of bare Au nanoparticles, which further confirm that the electrochemical and spectroscopic signals observed in the core-shell electrodes originate predominantly from the MnO_2 shell rather than the Au core (Supplementary Fig. 20)."

To exclude the contribution of the Au core to the capacitance, we compared Au@25 nm Cu-pi-MnO_2 with Au@25 nm MnO_2 at identical Au loadings. As shown in Fig. 3b, Au@25 nm Cu-pi-MnO_2 exhibits higher capacitance than undoped Au@25 nm MnO_2. This enhancement mirrors the trend observed for Cu-pi-δ-MnO_2 relative to undoped δ-MnO_2 (Fig. 1b). On page 7, we write:

"Cu^{2+} pre-intercalation within the 25 nm MnO_2 shell leads to a substantial enhancement in capacitance, reaching a maximum value of approximately 1101 F g^{-1} at 20 A g^{-1}, demonstrating that Cu incorporation promotes charge storage (Fig. 3b)."

We added new GCD data for Au@25 nm Cu-pi-MnO_2 (Supplementary Fig. 21a), showing a charge-discharge behavior similar to Au@2 nm MnO_2, exhibiting a more pronounced voltage plateau indicative of a proton-dominated redox process. CV and *in-situ* Raman spectroscopy further show signatures of a proton-coupled electron transfer (PCET) process in Au@25 nm Cu-pi-MnO_2 (Supplementary Fig. 22). Together, these observations indicate that the enhanced capacitance of Au@25 nm Cu-pi-MnO_2 arises from Cu^{2+}-induced promotion of proton-coupled charge storage. On page 8, we write:

"Consistent with this enhancement, GCD profiles of Au@25 nm Cu-pi-δ-MnO_2 measured in 0.1 M KOH, which closely resemble those of the 2 nm system and exhibit a more pronounced voltage plateau indicative of a proton-dominated redox process (Supplementary Fig. 21a)…Further evidence is provided by CV and in-situ Raman spectroscopy, which show clear signatures of a PCET process in Au@25 nm Cu-pi-δ-MnO_2 (Supplementary Fig. 22).".

Reviewer #1, comment 3. The confined water and its hydrogen-bonded network are key to the proposed mechanism. Could the authors provide evidence that this network is stable after prolonged electrochemical cycling?

Response

To address this point, we provide complementary electrochemical and spectroscopic evidence for the stability of the confined water network. Galvanostatic charge-discharge (GCD) measurements of Au@25 nm Cu-pi-MnO_2 over 2000 charge-discharge cycles (Supplementary Fig. 21a) show a charge-discharge behavior similar to Au@2 nm MnO_2, indicative of a proton-dominated redox process.

Meanwhile, *in-situ* Raman measurements at 0.4 V vs. RHE after 2000 cycles show clear vibrational signatures associated with confined interlayer water (Supplementary Fig. 21b). Together, these results demonstrate that the confined, hydrogen-bonded water network remains stable during long-term cycling. On page 8, we write:

"In addition, spectroscopic results show that clear signatures associated with confined interlayer water persist even after 2000 charge-discharge cycles (Supplementary Fig. 21b)."

Reviewer #1, comment 4. Although it is suggested that Ni^{2+} substitutes Mn sites instead of occupying interlayer sites, this conclusion would be strengthened by direct structural characterization (e.g., XANES) of the Ni^{2+} local coordination environment.

Response

We have removed the Ni-doping data and related discussion, as the local coordination environment of Ni^{2+} cannot be conclusively resolved within the scope of this revision.

Reviewer #1, comment 5. Please check the format problem further. For example, the label for Figure 3d is given as "Au@2 nm MnO_2 NPs", which seems to be a duplicate of Figure 3c. Please check.

Response:

This labeling error has now been corrected.

Reviewer #1, comment 6. Regarding methods, it's not clear what has been utilized from the existing work and what is new. It should be clearly discussed. Some important refs may be cited to enrich reference part of the revised manuscript, such as, Electrochem. Energy Rev. 2023, 6(1), 5; Electrochem. Energy Rev. 2023, 6, 33.

Response:

In the Methods and Conclusion section, we now clarify the rationale behind key experimental design choices that go beyond established protocols. We cite the recommended references and discuss them on page 5:

"A clear understanding of proton- and cation-based charge-storage mechanisms is critically important for other electrochemical energy-storage systems, such as Zn/MnO_2 batteries [28, 29]"

Reviewer #2 (Remarks to the Author):

The charge storage capacitance of delta-MnO_2 pseudocapacitors originates from two mechanisms, the intercalation of cations and protons as well as their adsorption on the surface. The introduction of Cu ions suppresses structural changes of the layered phase and enhances the capacitor performance. In this study, the authors investigated the role of Cu ions by combining electrochemical measurements with in situ surface-enhanced Raman spectroscopy under various conditions such as pH, film thickness, and electrolyte cation species. They demonstrated a correlation between surface structural changes and film performance, namely the emergence of a proton-coupled redox mechanism mediated by interlayer water molecules and the observed enhancement in capacitor performance. The synthesis of the samples and their physicochemical characterization were adequately carried out and explained in a logical manner (Fig. 1). The enhancement of capacitance upon Cu pre-intercalation was also experimentally demonstrated (Figs. 2e and 2f). However, there remain concerns regarding the novelty of the spectroscopic methodology, the interpretation of the interfacial processes, the analysis of the data, and the consistency of the explanations. For these reasons, I do not consider the work to meet the standards

required for publication in Nature Communications. I therefore cannot recommend the manuscript for further consideration for publication in this journal.

Major Points

Use of Raman Enhancement Substrates

Reviewer #2, comment 1. Gold nanoparticles coated with Mn oxide layers of different thicknesses were synthesized and employed as Raman enhancement elements. However, this approach may lead to the observation of surface information based on fundamentally different principles depending on the coating thickness. The SHINERS method (Li et al., Nature, 2010 and subsequent works) has already clarified the relation between oxide shell thickness and Raman enhancement, showing that the signal decays exponentially with increasing thickness. The enhancement is effective only within a few nanometers and is severely attenuated beyond ~10 nm. In this study, Mn oxide coatings of 2 nm and 25 nm were prepared and used. At 2 nm, selective enhancement of interfacial Raman signals can be expected, whereas at 25 nm, little enhancement should remain, and the measurement essentially corresponds to normal Raman spectroscopy. Indeed, in the thin-shell case, detailed potential dependence was observed even in the low-frequency region, while in the thick-shell case, the spectra were weaker, broader, and lacked clear potential dependence. Thus, the former likely provides selective surface information, whereas the latter reflects bulk-averaged information. Direct comparison of the two cases may lead to misinterpretation, and this requires careful consideration. Nevertheless, the 2 nm case seems appropriate for the authors' intended purpose of probing surface behavior in situ condition. If one intends to obtain molecular information from the surface of a 25-nm Mn Oxide film using Raman spectroscopy for comparison purpose with that of 2-nm shell, this can be achieved by employing the SHINERS method, which is applicable even under strong alkaline and electrochemical conditions. In practice, this would involve placing shell-isolated nano-amplifiers, resistant to concentrated alkali, on the Mn oxide surface. Recently, it was reported that the zirconia shell-isolated nano-amplifiers enable electrochemical in situ Raman measurement in 1 M KOH solution.

Response:

We agree that plasmonic Raman enhancement decays exponentially with increasing oxide-shell thickness, as well established by the SHINERS literature, such that effective enhancement is largely confined to the near-surface region within a few nanometers. We designed the comparison between 2 nm and 25 nm MnO_2 shells based on this principle.

Our mechanistic conclusions regarding surface water-mediated PCET are therefore based primarily on the 2 nm-shell samples, for which enhancement of interfacial vibrational modes is expected and is clearly observed. The 25 nm-shell MnO_2 samples serve as a complementary reference to distinguish surface-dominated PCET behavior from bulk-dominated cation-intercalation processes.

Au@25 nm Cu-pi-MnO_2 architecture was designed to probe water molecules located within the MnO_2 interior. Here, Raman enhancement of outermost surface-adsorbed water is negligible. Therefore, the appearance of an O-H stretching feature is attributed to water

confined within the MnO_2 interlayers rather than surface-adsorbed species. This interpretation is further supported by the absence of a corresponding water signal in pristine Au@25 nm MnO_2, providing an internal control.

We now write on page 3:

"Given the rapid decay of Au-core-induced Raman enhancement with increasing MnO_2 shell thickness,[27] this signal is attributed to confined water within the MnO_2 interlayers, with the elevated frequency reflecting disruption of the hydrogen-bond network under nanoconfinement."

On page 7:

"To test this hypothesis, we engineered Au@MnO_2 core-shell structures with varied MnO_2 shell thicknesses to decouple surface-dominated proton-coupled processes from bulk cation intercalation (Fig. 3a)."

Reviewer #2, comment 2. **Assignment of Raman Bands.** The manuscript assigns Raman bands at 727, 504, 461, and 413 cm^{-1} (or 808, 503, 457, 410 cm^{-1}) to MnOOH, but no supporting references or computational evidence are provided. Previous reports consistently assign MnOOH signals around 625, 560, and 530 cm^{-1}, which differ markedly from the present results. While the emergence of new Raman bands concomitant with redox processes in CV is understandable, this does not constitute proof of MnOOH formation. The basis for the current assignment must be clarified. Furthermore, the manuscript states that these Raman bands shift to 492, 447, and 401 cm^{-1} upon using D_2O, attributed to an isotope effect. However, if these bands corresponded to OH-related vibrations, both stretching and bending modes should shift to ~0.71-fold of the original wavenumber. In contrast, the observed shifts correspond to ~0.97-fold, suggesting instead that these are modes such as O-Mn-O bending or lattice vibrations in which hydrogen (or deuterium) contributes minimally. The authors should discuss this possibility explicitly and provide a consistent explanation for the assignments, isotope shifts, and surface structures inferred from the spectra.

Response:

We agree that the Raman bands observed in our study differ from those of crystalline MnOOH reported in the literature. These bands do not correspond to a stable MnOOH phase; rather, they represent a transient MnOOH-like intermediate generated operando during the PCET reaction.

We performed time-resolved operando Raman measurements by applying 0.0 V (vs. RHE) for 5 min and then instantaneously returning to open-circuit potential while continuously acquiring spectra. In all five repeated cycles, the MnO_2 features reappeared immediately upon potential removal, demonstrating that the MnOOH-like species forms only under applied bias (Supplementary Fig. 19). We included these observations on page 7:

"Notably, the MnOOH species formed during this process is a transient and reversible intermediate that converts back to MnO_2 upon removal of the applied potential (Supplementary Fig. 19)."

To further substantiate the vibrational assignments and strengthen the mechanistic interpretation, we performed isotopic labeling experiments on Au@2 nm MnO_2 using D_2O. The observed isotope-dependent frequency shifts provide direct evidence for the proton- and water-related origin of the newly emerging Raman bands, enabling their reliable assignment (Supplementary Fig. 18). At ~0.7 V, water adsorption on the MnO_2 surface becomes evident, and the water-related bands observed at ~3646, ~3485, and ~3412 cm^{-1} in H_2O exhibit pronounced Stark shifts under the interfacial electric field. Upon replacing H_2O with D_2O, these features systematically red-shift to ~2685, ~2585, and ~2533 cm^{-1}, consistent with O-H to O-D isotopic substitution, confirming their assignment to interfacial water species strongly interacting with the MnO_2 surface.

As the potential is decreased below ~0.6 V, the characteristic Mn-O vibrational modes of MnO_2 diminish, indicating the onset of a redox reaction between MnO_2 and interfacial water ($MnO_2 + H_2O + e^- \rightarrow MnOOH + OH^-$). Concomitantly, new vibrational features emerge. In particular, a band at ~807 cm^{-1}, which red-shifts to ~607 cm^{-1} in D_2O, is consistent with the O-H bending mode of an MnOOH-like species. Additional bands at ~502, ~457, and ~410 cm^{-1}, assigned to Mn-O vibrations of MnOOH, exhibit modest but systematic red shifts to ~488, ~432, and ~396 cm^{-1} in D_2O. These isotopic shifts arise from the adsorption of water molecules on the Mn-O framework of the MnOOH surface, forming a hydrogen-bonded configuration (H-O-H···O=Mn-O-H), which is perturbed upon isotopic substitution to D-O-D···O=Mn-O-D. Because all lattice oxygen atoms are bonded to hydrogen, substitution with deuterium leads to corresponding shifts in all Mn-O vibrational modes.

These deuterium-labeling experiments have been included in Supplementary Fig. 18, together with a detailed description of the vibrational peak assignment and analysis. On page 7, we now state:

"*CV and in-situ Raman spectroscopy show a proton-coupled electron transfer (PCET) process in the ultrathin 2 nm MnO_2 system (Figs. 3e,f), as evidenced by the emergence of characteristic Mn-O vibrations (~502, ~457, and ~410 cm^{-1}), O-H bending (~807 cm^{-1}), and O-H stretching (~3577 cm^{-1}) of MnOOH, together with bands associated with interfacial water (~3412, ~3485, and ~3646 cm^{-1}). The vibrational assignments are further corroborated by isotopic (D_2O) Raman experiments (Supplementary Fig. 18).*"

Reviewer #2, comment 3. **Experimental Parameters and Comparisons.** The study varies several parameters simultaneously (electrolyte concentration, MnO_2 film thickness, presence/absence of Cu, cation species), but comparisons based on multiple changing parameters do not allow for clear conclusions. Only one parameter should be varied at a time. While a broad range of observations is presented, the discussion becomes complex and does not arrive at a comprehensive understanding.

Response:

In this work, we performed the studies following this order:

First, the comparison between Au@25 nm MnO_2 and Au@25 nm Cu-pi-MnO_2 was designed to explore the role of Cu incorporation.

Second, we varied MnO_2 shell thickness to decouple surface-dominated PCET from bulk cation-intercalation behavior.

Third, electrolyte concentration was varied to probe how interfacial ion chemistry regulates proton intercalation. These experiments show competition between OH^- and water molecules for active sites, which suppresses PCET at high OH^- concentrations. To exclude cation-specific effects arising from electrolyte changes, additional control experiments using different cations were conducted, confirming that the observed trends originate from OH^-–water competition.

We have now revised the relevant sections of the Results and Discussion (pages 4, 7, and 9) to explicitly state the purpose of each comparison, and updated the Conclusions section to emphasize the integrated, mechanistically driven experimental design.

Minor Points

Reviewer #2, comment 4. The synthesis of gold nanoparticles is described using the traditional Frens method with Na citrate reported in Nature 1973. However, the original procedure uses a gold salt concentration of 0.01%; this should be checked for consistency.

Response:
We now correct the gold salt concentration to 0.01 wt% $HAuCl_4$.

Reviewer #2, comment 5. The reported maximum capacitance values should be compared against benchmark values from previous studies on similar materials; otherwise, their significance remains unclear.

Response:

We now added explicit benchmark comparisons with representative studies on similar MnO_2-based materials in the revised performance section.

Reviewer #2, comment 6. The authors suggest that Cu ions substitute for K ions. However, the EDX mapping shown in Fig. 1g and Supplementary Fig. 4 does not support Cu distribution in the spine-like structures visible at the lower center of the image. Is there direct evidence that Cu ions are incorporated into the crystal interior rather than merely adsorbed at the surface other than XRD data?

Response (Reviewer #2 and Reviewer #4):

In addition to XRD, we examined Cu incorporation using ICP-OES and XPS. δ-MnO_2 is a layered material in which interlayer K^+ ions act as structural pillars to maintain charge neutrality. Bulk ICP-OES analysis (Table S1) shows a lower K^+ content, from $K_{0.125}MnO_2$ to $K_{0.011}Cu_{0.089}MnO_2$, indicating that Cu incorporation proceeds via replacement of interlayer K^+ rather than surface adsorption, consistent with prior reports on cation-exchanged birnessite systems (*Langmuir* 2015, 31, 12807-12813; RSC Adv. 2024, 14, 10191–10198; Nat. Commun. 2017, 8, 14424).

Replacement of monovalent K^+ by divalent Cu^{2+} requires charge compensation. Consistently, XPS shows partial Mn reduction together with an increased population of Mn-OH/H_2O-related species, indicating redox and coordination reorganization that

stabilizes Cu^{2+} within the layered host; this interpretation is further supported by DFT calculations, which suggest preferential interlayer incorporation of hydrated Cu^{2+} (Fig.1f).

HAADF, SAED, and HRTEM show decreased interlayer spacing from 0.73 to 0.68 nm upon Cu^{2+} pre-insertion (Fig. 1c-e), in alignment with XRD. Surface adsorption is unlikely to change the interlayer spacing.

Reviewer #2, comment 7. In Fig. 1b, it would be informative to include capacitance data for MnO_2 without Cu ions in 4 M KOH electrolyte. This would allow for a direct comparison of the effects of electrolyte concentration and Cu incorporation.

Response:

Following this comment, we have added the capacitance data for pristine δ-MnO_2 measured in 4 M KOH to Fig. 1b, showing that higher electrolyte concentration generally promotes charge-storage capability. On page 3, we write:

Cu^{2+} pre-intercalation markedly enhances the capacitance, yielding ~560 F g^{-1} at 20 A g^{-1} in 1 M KOH, compared to ~160 F g^{-1} for pristine δ-MnO_2 (Fig. 1b). Increasing the electrolyte concentration to 4 M KOH further improves the capacitance of both systems, with Cu-pi-MnO_2 reaching ~650 F g^{-1} even at a high current density of 70 A g^{-1}.[24-26]

Overall Assessment. While the manuscript demonstrates careful experimental work and highlights an important problem, the spectroscopic assignments and data interpretation lack sufficient rigor and novelty for Nature Communications. Significant revisions and more conclusive evidence would be required before further consideration.

Reviewer #3 (Remarks to the Author):

This work investigates the mechanistic origins of the enhanced capacitance in δ-MnO_2 with pre-intercalated Cu^{2+}. Transition metal pre-intercalation promotes the retention of interlayer-confined water, and this supports proton-coupled charge storage. Similar mechanistic transition is also evident in δ-MnO_2 systems pre-intercalated with other transition metal ions, such as Co^{2+} and Mg^{2+}. This work offers a route to design high-performance MnO_2 electrodes. I recommend its publication after major revision

Reviewer #3, comment 1. In this work, Au@δ-MnO_2 and Au@Cu-pi-δ-MnO_2 core-shell nanostructures were prepared for SERS. The introduction of Au core will change the electrical conductivity and transmission path of cation to proton. They would influence charge storage. Related experiments and discussion could be included in this work.

Response:

Our control experiments and comparative analyses indicate that, while the Au core enhances Raman sensitivity, it does not alter the intrinsic redox or proton-coupled charge storage mechanism of the MnO_2 shell. We performed control CV and *in-situ* SERS measurements on bare Au nanoparticles (Supplementary Fig. 20), finding Au oxidation potentials > 1.29 V vs RHE. We do not observe Raman features associated with Au-O

species in the potential range of 1.10 to -0.40 V vs RHE. These exclude the contributions of Au core to the electrochemical and spectroscopic signals. These are summarized in Supplementary Fig. 20. In page 7, we write:

"As an additional control, we also present the CV and in-situ SERS results of bare Au nanoparticles, which further confirm that the electrochemical and spectroscopic signals observed in the core-shell electrodes originate predominantly from the MnO_2 shell rather than the Au core (Supplementary Fig. 20)."

To exclude the contribution of the Au core to the capacitance, we compared Au@25 nm Cu-pi-MnO_2 with Au@25 nm MnO_2 at identical Au loadings. As shown in Fig. 3b, Au@25 nm Cu-pi-MnO_2 exhibits higher capacitance than undoped Au@25 nm MnO_2. This enhancement mirrors the trend observed for Cu-pi-δ-MnO_2 relative to undoped δ-MnO_2 (Fig. 1b). On page 7, we write:

"Cu^{2+} pre-intercalation within the 25 nm MnO_2 shell leads to a substantial enhancement in capacitance, reaching a maximum value of approximately 1101 F g^{-1} at 20 A g^{-1}, demonstrating that Cu incorporation promotes charge storage (Fig. 3b)."

We added new GCD data for Au@25 nm Cu-pi-MnO_2 (Supplementary Fig. 21a), showing a charge-discharge behavior similar to Au@2 nm MnO_2, exhibiting a more pronounced voltage plateau indicative of a proton-dominated redox process. CV and *in-situ* Raman spectroscopy further show signatures of a proton-coupled electron transfer (PCET) process in Au@25 nm Cu-pi-MnO_2 (Supplementary Fig. 22). Together, these observations indicate that the enhanced capacitance of Au@25 nm Cu-pi-MnO_2 arises from Cu^{2+}-induced promotion of proton-coupled charge storage. On page 8, we write:

"Consistent with this enhancement, GCD profiles of Au@25 nm Cu-pi-δ-MnO_2 measured in 0.1 M KOH, which closely resemble those of the 2 nm system and exhibit a more pronounced voltage plateau indicative of a proton-dominated redox process (Supplementary Fig. 21a)…Further evidence is provided by CV and in-situ Raman spectroscopy, which show clear signatures of a PCET process in Au@25 nm Cu-pi-δ-MnO_2 (Supplementary Fig. 22)."

Reviewer #3, comment 2. In some reference, the MnO_2 without pre-intercalated metal ions also display proton-coupled charge storage. Authors could further clarify the conclusion of this work.

Response:

We agree with the reviewer that we should not exclude the proton participation in pristine MnO_2 reported in other studies. Our results indicate that such proton-related contributions are not dominant under the specific conditions examined here.

We now clarify on page 7:

" Based on our observations and prior reports that pristine birnessite-type MnO_2 can exhibit enhanced capacitance via proton-associated surface or near-surface faradaic

reactions,[7,30] we attribute the capacitance enhancement of Cu-pi-δ-MnO_2 to promoted proton-coupled charge storage."

Reviewer #3, comment 3. In this work, SERS was used to investigate proton-coupled charge storage. More characterizations could be carried out to understand the proton-coupled charge storage.

Response:
We have strengthened our conclusions by adding new GCD measurements, which show that Au@25 nm Cu-pi-MnO_2 (Supplementary Fig. 21a) exhibits pronounced voltage plateaus compared with Au@25 nm MnO_2, closely resembling the behavior of proton-dominated Au@2 nm MnO_2. Spectroscopic results further show that clear signatures of confined interlayer water persist even after 2000 charge-discharge cycles (Supplementary Fig. 21b). In addition, new capacitance measurements demonstrate significantly enhanced performance for Au@25 nm Cu-pi-MnO_2 (Fig. 3b). Together with CV results and *in-situ* Raman evidence of a proton-dominated redox pathway (Supplementary Fig. 22), these data consistently indicate that Cu^{2+} pre-intercalation promotes proton-coupled charge storage.

Reviewer #3, comment 4. The size of δ-MnO_2 would also influence charge storage. Related experiments and discussion could be included in this work.

Response:

We agree with the reviewer that the size (or thickness) of δ-MnO_2 strongly influences charge-storage behavior. This effect is explicitly captured by our core-shell design: ultrathin MnO_2 favors surface-dominated proton-coupled processes, whereas thicker MnO_2 primarily exhibits bulk cation-intercalation behavior, as discussed in the revised manuscript (page 7).

This thickness-controlled architecture is intended as a mechanistic proof-of-concept rather than a scalable optimization strategy, as the large-scale synthesis of ultrathin MnO_2 with well-defined thickness remains technically challenging. Nevertheless, it enables direct interrogation of how reducing MnO_2 thickness promotes surface-confined, reversible charge-storage pathways.

We have clarified this point in the revised manuscript (page 7):

"*To test this hypothesis, we engineered Au@MnO_2 core-shell structures with varied MnO_2 shell thicknesses to decouple surface-dominated proton-coupled processes from bulk cation intercalation (Fig. 3a). This design serves as a mechanistic proof-of-concept, since the scalable synthesis of ultrathin MnO_2 remains technically challenging.*"

Reviewer #3, comment 5. To illustrate the feasibility of pre-intercalated Cu^{2+}, large-capacity full batteries could be assembled and measured.

Response:
We have performed preliminary aqueous Zn-ion battery tests, the results of which are

shown in Fig. R3-1, following this protocol: the active mass loading of the cathodes (on stainless-steel foil) was 2.1 mg cm^{-2}, with a composition of MnO_2 : carbon black : CMC : SBR = 70 : 20 : 2.5 : 7.5. An acidic 2 M $ZnSO_4$ aqueous electrolyte was used with a glass fiber separator (GF/A grade). Cycling tests were carried out at a current density of 1 C (308 mA g^{-1}) within a potential window of 0.8-1.9 V.

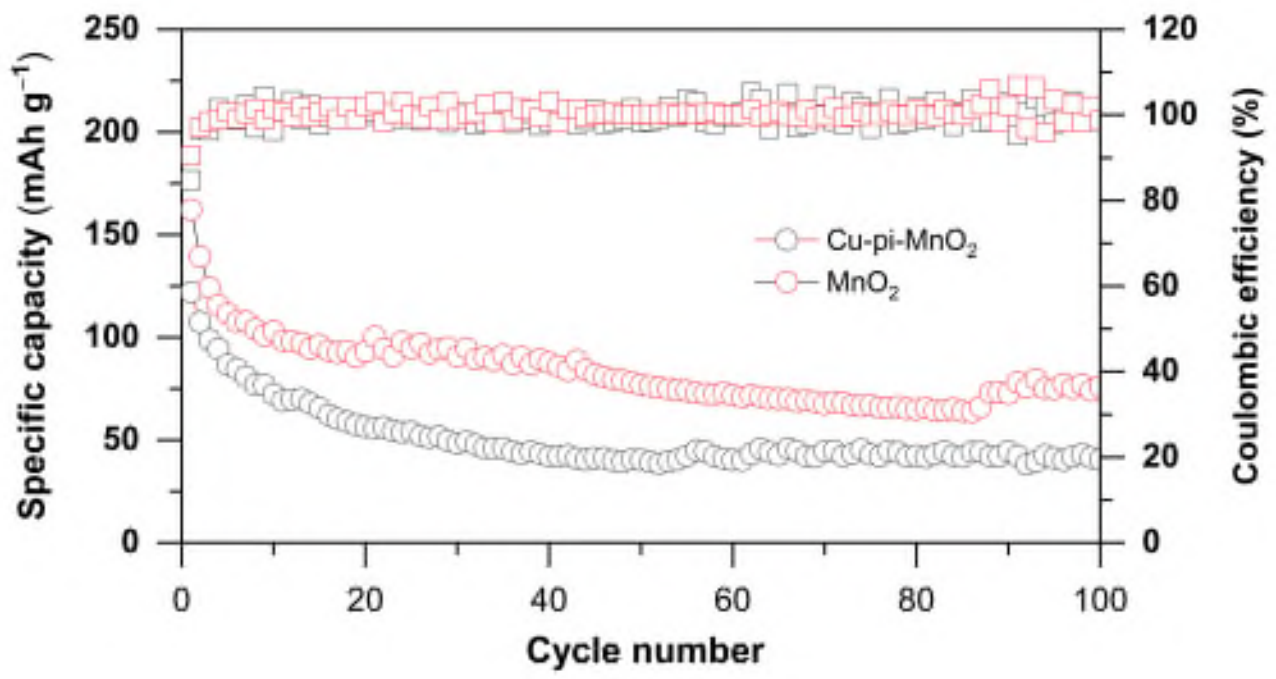

Figure R3-1| Aqueous Zn-ion battery performance test of Cu-pi-MnO_2 and MnO_2

Reviewer #4 (Remarks to the Author):

The authors investigate the regulation of the charge-storage mechanism in δ-MnO_2 through metal-ion pre-intercalation. The study presents a conceptually interesting idea and attempts to correlate structural, electrochemical, and spectroscopic observations to the behavior of interlayer water and proton transport. However, the current experimental evidence is insufficient to support the proposed mechanism, and critical structural information remains unclear. There are still several fundamental issues and mechanistic details that need to be resolved; therefore, the manuscript is not yet suitable for publication in Nature Communications.

Reviewer #4, comment 1. Regarding the “water-retention effect,” the authors mainly rely on Raman peak shifts without providing quantitative or direct evidence to verify the presence and variation of interlayer water.

Response:

We agree that Raman spectroscopy does not provide a quantitative measure of the absolute amount of interlayer water. However, under operando electrochemical conditions, spectroscopic techniques currently offer the most direct means to probe the presence and dynamic evolution of interfacial and confined water. To further substantiate our Raman observations, we have added complementary electrochemical evidence that is fully consistent with a water-mediated, proton-dominated charge-storage process.

Newly added GCD measurements show that Au@25 nm Cu-pi-MnO_2 (Supplementary Fig. 21a) exhibits pronounced voltage plateaus compared with Au@25 nm MnO_2 (Fig. 3c), closely resembling the proton-dominated behavior of Au@2 nm MnO_2 (Fig. 3d). Consistently, CV measurements show that with continued cycling, the electrochemical response of Au@25 nm Cu-pi-MnO_2 (Supplementary Fig. 22) progressively approaches that of Au@2 nm MnO_2 (Fig. 3e), while remaining clearly distinct from Au@25 nm MnO_2

(Fig. 3g). This convergence in electrochemical fingerprints provides functional evidence for enhanced proton participation associated with water retention.

Reviewer #4, comment 2. The discussion of Ni-ion doping is mainly based on references concerning α-MnO_2, whereas the material studied here is δ-MnO_2 with a different framework structure. The authors should clarify how such structural differences affect the behavior of Cu^{2+}, and further distinguish between the mechanisms of lattice doping and interlayer intercalation, as well as explain why Cu^{2+} tends to remain interlayered rather than incorporated into the lattice.

Response:

We have removed the Ni-doping data and related discussion, as the local coordination environment of Ni^{2+} cannot be conclusively resolved within the scope of this revision.

For Cu-pi-δ-MnO_2, XRD shows a clear contraction of the interlayer spacing, while EDS, XPS, and ICP-OES analyses (Table S1) show a pronounced decrease in K content, from $K_{0.125}MnO_2$ for δ-MnO_2 to $K_{0.011}Cu_{0.089}MnO_2$ for Cu-pi-δ-MnO_2, indicating replacement of interlayer K^+ by Cu^{2+}. Consistently, DFT calculations indicate that interlayer Cu^{2+} is energetically favored through electrostatic stabilization and coordination with interlayer water. Given the layered framework of δ-MnO_2, lattice substitution would require substantial distortion of the MnO_6 network and is therefore unfavorable. These points are discussed in detail in the main text (page 3).

We address these two comments together:

Reviewer #4, comment 3. The high specific capacitance value obtained after "subtracting the Au contribution" may be overestimated, as interfacial coupling effects are generally not linearly separable. Such a correction method could misrepresent the intrinsic performance of the active material.

Reviewer #4, comment 7. The Au@MnO_2 core-shell structure (~1 μg active material) differs markedly from practical electrodes. The Au core may alter the electrochemical response and Raman signals via enhanced conductivity and local field effects, so its influence should be carefully considered when interpreting the intrinsic behavior of MnO_2.

Response:

We have revised our analysis to ensure full transparency. We now present the raw, uncorrected capacitance data (without subtracting the Au contribution) in the main text. To quantitatively assess the influence of Au and to account for differences in Au content at similar MnO_2 loadings, we measured the capacitance of bare Au nanoparticles in each system. These data are provided in Table S2. On page 7, we write:

" To account for the different Au contents at identical MnO_2 loadings, the corresponding capacitance responses of the Au nanoparticles in both systems are provided in Table S2."

We performed control CV and *in-situ* SERS measurements on bare Au nanoparticles (Supplementary Fig. 20), finding Au oxidation potentials > 1.29 V vs RHE. We do not observe Raman features associated with Au-O species in the potential range of 1.10 to -0.40 V vs RHE. These exclude the contributions of Au core to the electrochemical and spectroscopic signals. These are summarized in Supplementary Fig. 20. In page 7, we write:

"As an additional control, we also present the CV and in-situ SERS results of bare Au nanoparticles, which further confirm that the electrochemical and spectroscopic signals observed in the core-shell electrodes originate predominantly from the MnO_2 shell rather than the Au core (Supplementary Fig. 20)."

To exclude the contribution of the Au core to the capacitance, we compared Au@25 nm Cu-pi-MnO_2 with Au@25 nm MnO_2 at identical Au loadings. As shown in Fig. 3b, Au@25 nm Cu-pi-MnO_2 exhibits higher capacitance than undoped Au@25 nm MnO_2. This enhancement mirrors the trend observed for Cu-pi-δ-MnO_2 relative to undoped δ-MnO_2 (Fig. 1b). On page 7, we write:

"Cu^{2+} pre-intercalation within the 25 nm MnO_2 shell leads to a substantial enhancement in capacitance, reaching a maximum value of approximately 1101 F g^{-1} at 20 A g^{-1}, demonstrating that Cu incorporation promotes charge storage (Fig. 3b)."

We added new GCD data for Au@25 nm Cu-pi-MnO_2 (Supplementary Fig. 21a), showing a charge-discharge behavior similar to Au@2 nm MnO_2, exhibiting a more pronounced voltage plateau indicative of a proton-dominated redox process. CV and *in-situ* Raman spectroscopy further show signatures of a proton-coupled electron transfer (PCET) process in Au@25 nm Cu-pi-MnO_2 (Supplementary Fig. 22). Together, these observations indicate that the enhanced capacitance of Au@25 nm Cu-pi-MnO_2 arises from Cu^{2+}-induced promotion of proton-coupled charge storage. On page 8, we write:

"Consistent with this enhancement, GCD profiles of Au@25 nm Cu-pi-δ-MnO_2 measured in 0.1 M KOH, which closely resemble those of the 2 nm system and exhibit a more pronounced voltage plateau indicative of a proton-dominated redox process (Supplementary Fig. 21a)…Further evidence is provided by CV and in-situ Raman spectroscopy, which show clear signatures of a PCET process in Au@25 nm Cu-pi-δ-MnO_2 (Supplementary Fig. 22).".

Reviewer #4, comment 4. The current characterization results only confirm the presence of Cu in the samples but are insufficient to demonstrate that Cu ions are indeed located within the interlayer region and remain stable during cycling. It is recommended to examine the electrochemical performance of Cu-pi-MnO_2 electrodes without the Au core to verify whether the proposed mechanism holds in a practical configuration. (Reviewer #4)

Response:

In addition to XRD, we examined Cu incorporation using ICP-OES and XPS. δ-MnO_2 is a layered material in which interlayer K^+ ions act as structural pillars to maintain charge neutrality. Bulk ICP-OES analysis (Table S1) shows a lower K^+ content, from $K_{0.125}MnO_2$ to $K_{0.011}Cu_{0.089}MnO_2$, indicating that Cu incorporation proceeds via replacement of interlayer K^+ rather than surface adsorption, consistent with prior reports on cation-

exchanged birnessite systems (*Langmuir* 2015, 31, 12807-12813; RSC Adv. 2024, 14, 10191-10198; Nat. Commun. 2017, 8, 14424).

Replacement of monovalent K^+ by divalent Cu^{2+} requires charge compensation. Consistently, XPS shows partial Mn reduction together with an increased population of Mn-OH/H_2O-related species, indicating redox and coordination reorganization that stabilizes Cu^{2+} within the layered host; this interpretation is further supported by DFT calculations, which suggest preferential interlayer incorporation of hydrated Cu^{2+} (Fig.1f).

HAADF, SAED, and HRTEM show decreased interlayer spacing from 0.73 to 0.68 nm upon Cu^{2+} pre-insertion (Fig. 1c-e), in alignment with XRD. A surface adsorption is unlikely to change the interlayer spacing.

Finally, electrochemical measurements performed on Cu-pi-MnO_2 electrodes prepared without the Au core show the same enhanced capacitance as Au-core samples, confirming that the proposed mechanism is intrinsic to the Cu-pre-intercalated MnO_2 framework and independent of the Au core. We now provide practical battery performance using Cu-pi-MnO_2 without Au core (see the response to Reviewer #3, comment 5).

Reviewer #4, comment 5. Since Cu^{2+} carries a higher charge than K^+, charge compensation through valence adjustment or anion incorporation should occur; however, this aspect has not been addressed or discussed in the manuscript.

Response:

We now discuss this charge compensation mechanism in Supplementary Fig. 6 and in the main text (page 3):

"X-ray photoelectron spectroscopy (XPS) analysis (Supplementary Figs. 5-6) indicates that Mn^{4+} remains dominant, with partial Mn reduction upon Cu^{2+} pre-intercalation. Concurrently, the O 1s spectrum shows an increased population of Mn-OH/H_2O species. These changes together compensate for the additional charge introduced by Cu^{2+} pre-intercalation."

Reviewer #4, comment 6. The manuscript presents electrochemical data for Co^{2+}- and Mg^{2+}-intercalated samples. Given the significant differences in ionic radius, charge density, and coordination characteristics among these cations, additional structural and spectroscopic analyses are required to determine whether these ions act via similar mechanisms.

Response

We have performed additional *in-situ* electrochemical SERS measurements on Co^{2+}- and Mg^{2+}-pre-intercalated MnO_2. Consistent with the electrochemical CV results, these in situ spectroscopic data show that the effects of Co^{2+} and Mg^{2+} pre-intercalation are analogous to Cu^{2+}. The corresponding spectroscopic data have been added to the Supplementary Information (Supplementary Figs. 16-17). On the main text page 7, we now write:

The spectroscopic and electrochemical results suggest that pre-intercalation of $Co^{2+}$and Mg^{2+}, which replace native K^+ ions, facilitates the insertion of interlayer-confined water and similarly promotes proton-coupled intercalation (Supplementary Figs. 16-17).

We now provide XRD measurements showing successful pre-intercalation of Co^{2+} and Mg^{2+} into the interlayer spacing of MnO_2 (Supplementary Figs. 15), updating on page 6:

"XRD measurements of Co-pi-δ-MnO_2 and Mg-pi-δ-MnO_2 similarly reveal a contraction in interlayer spacing, confirming successful pre-intercalation(Supplementary Fig. 15)."
